# Adaptation to High Ethanol Reveals Complex Evolutionary Pathways

**DOI:** 10.1371/journal.pgen.1005635

**Published:** 2015-11-06

**Authors:** Karin Voordeckers, Jacek Kominek, Anupam Das, Adriana Espinosa-Cantú, Dries De Maeyer, Ahmed Arslan, Michiel Van Pee, Elisa van der Zande, Wim Meert, Yudi Yang, Bo Zhu, Kathleen Marchal, Alexander DeLuna, Vera Van Noort, Rob Jelier, Kevin J. Verstrepen

**Affiliations:** 1 VIB Laboratory for Systems Biology, Leuven, Belgium; 2 CMPG Laboratory for Genetics and Genomics, KU Leuven, Leuven, Belgium; 3 CMPG Laboratory of Predictive Genetics and Multicellular Systems, KU Leuven, Leuven, Belgium; 4 Laboratorio Nacional de Genómica para la Biodiversidad, Centro de Investigación y de Estudios Avanzados del IPN, Irapuato, Guanajuato, Mexico; 5 CMPG Department of Microbial and Molecular Systems, KU Leuven, Leuven, Belgium; 6 Department of Information Technology (INTEC, iMINDS), University of Ghent, Ghent, Belgium; 7 CMPG Laboratory of Computational Systems Biology, KU Leuven, Leuven, Belgium; 8 Department of Plant Biotechnology and Bioinformatics, University of Ghent, Ghent, Belgium; University of Michigan, UNITED STATES

## Abstract

Tolerance to high levels of ethanol is an ecologically and industrially relevant phenotype of microbes, but the molecular mechanisms underlying this complex trait remain largely unknown. Here, we use long-term experimental evolution of isogenic yeast populations of different initial ploidy to study adaptation to increasing levels of ethanol. Whole-genome sequencing of more than 30 evolved populations and over 100 adapted clones isolated throughout this two-year evolution experiment revealed how a complex interplay of *de novo* single nucleotide mutations, copy number variation, ploidy changes, mutator phenotypes, and clonal interference led to a significant increase in ethanol tolerance. Although the specific mutations differ between different evolved lineages, application of a novel computational pipeline, PheNetic, revealed that many mutations target functional modules involved in stress response, cell cycle regulation, DNA repair and respiration. Measuring the fitness effects of selected mutations introduced in non-evolved ethanol-sensitive cells revealed several adaptive mutations that had previously not been implicated in ethanol tolerance, including mutations in *PRT1*, *VPS7*0 and *MEX67*. Interestingly, variation in *VPS70* was recently identified as a QTL for ethanol tolerance in an industrial bio-ethanol strain. Taken together, our results show how, in contrast to adaptation to some other stresses, adaptation to a continuous complex and severe stress involves interplay of different evolutionary mechanisms. In addition, our study reveals functional modules involved in ethanol resistance and identifies several mutations that could help to improve the ethanol tolerance of industrial yeasts.

## Author Summary

Organisms can evolve resistance to specific stress factors, which allows them to thrive in environments where non-adapted organisms fail to grow. However, the molecular mechanisms that underlie adaptation to complex stress factors that interfere with basic cellular processes are poorly understood. In this study, we reveal how yeast populations adapt to high ethanol concentrations, an ecologically and industrially relevant stress that is still poorly understood. We exposed six independent populations of genetically identical yeast cells to gradually increasing ethanol levels, and we monitored the changes in their DNA sequence over a two-year period. Together with novel computational analyses, we could identify the mutational dynamics and molecular mechanisms underlying increased ethanol resistance. Our results show how adaptation to high ethanol is complex and can be reached through different mutational pathways. Together, our study offers a detailed picture of how populations adapt to a complex continuous stress and identifies several mutations that increase ethanol resistance, which opens new routes to obtain superior biofuel yeast strains.

## Introduction

The ability to survive and proliferate in high levels of ethanol is an ecologically important and industrially relevant trait of yeast cells. The ethanol produced by yeast cells slows down growth of competing microbes, but at higher concentrations, it causes stress for the yeast cells themselves. Different yeast strains show significant differences in their ability to grow in the presence of ethanol, with the more ethanol-tolerant ones likely having a fitness advantage over non-tolerant strains [1–3]. Moreover, ethanol tolerance is a key trait of industrial yeasts that often encounter very high ethanol concentrations, for example during beer and wine making and industrial bio-ethanol production.

Because of its industrial importance, there is great interest in fully understanding the genetic underpinnings of ethanol tolerance in microbes. Different experimental approaches have been used, including screening of (deletion) mutants for increased ethanol tolerance, transcriptome analysis of ethanol stressed cells and QTL analyses aimed at identifying mutations that cause differences in ethanol tolerance between different yeast strains [4–10]. Together, these studies have linked multiple different genetic loci to ethanol tolerance and identified hundreds of genes, involved in a multitude of cellular processes [11–14]. While it becomes increasingly clear that ethanol is a complex stress that acts on several different processes including increasing fluidity and permeability of cellular membranes, changing activity and solubility of membrane-bound and cytosolic proteins and interfering with the proton motive force (for review, see [15–17], the exact molecular mechanisms and genetic architecture underlying ethanol tolerance are still largely unknown.

Experimental evolution to study adaptation to increased ethanol levels could provide more insight into the molecular mechanisms underlying ethanol tolerance since such experiments could reveal different mutational paths that make a sensitive strain more tolerant. Only a handful of studies have looked at adaptation to ethanol in originally non-ethanol tolerant microbes exposed to gradually increasing levels of ethanol [6,18–22]. These have mostly focused on the physiological adaptations found in the evolved cells and have not performed an extensive analysis of the mechanisms and genetic changes underlying this adaptation. Hence, a comprehensive analysis of the type and number of mutations a non-ethanol tolerant strain can (or needs to) acquire to become more ethanol tolerant is still lacking.

Experimental evolution has proven to be a valuable tool to investigate the different mechanisms and pathways important for cells to adapt to specific selective conditions. Seminal papers have increased our understanding of the molecular basis of adaptation to specific stresses, such as heat stress, nutrient limitation and antibiotic treatment [23–28]. Recent advances in DNA sequencing technologies allow affordable and fast sequencing of complete genomes of clones and populations. While sequencing clones yields information on individual lineages within the experiment, population data provides information on the heterogeneity of adaptation. Additionally, sequencing samples isolated at different time points during the evolution experiment makes it possible to capture evolution in action. This has provided valuable information on the rate and types of mutations underlying adaptation, the genetic basis of ‘novel’ phenotypes and the existence of parallel pathways to establish comparable phenotypic outcomes [29–32].

For example, a common strategy observed in populations evolving under nutrient limitation is the amplification of genetic regions encoding transporters responsible for the uptake of the limiting nutrient [24,33]. Other studies using multiple replicate populations have discovered a high degree of parallelism in the adaptive solutions found by different populations. However, in other cases, the evolutionary pathways can be more complex. For example, clonal interference, the competition between lineages carrying different beneficial mutations, is a commonly observed phenomenon in evolution of asexually propagating populations that can increase complexity of mutational dynamics as well as impede the spread of beneficial mutations in a population. Clonal interference is typically expected to be prevalent in large populations that are adapting to a complex stress, where multiple adaptive mutations can occur [32,34,35].

To unravel the molecular mechanisms of adaptation to a specific condition, most studies have used isogenic replicate populations, with all cells having the same initial genome size. Genome size can significantly change during evolution; with both small-scale changes (chromosomal deletions and amplifications) and large-scale changes (increase or decrease in ploidy). Moreover, ploidy shifts have been reported in the evolutionary history of many organisms, including *Saccharomyces cerevisiae* and as a response to selective pressure [36–38]. Conversely, genome size has also been reported to affect evolution rate: polyploidy has been suggested to increase adaptability [39,40]. Multiplying the amount of DNA increases the genetic material available for evolution to tinker with and can alter gene expression [41–43]. These polyploid genomes can be unstable, resulting in loss of chromosomes and thus aneuploid cells [44–46]. Although studies have looked at adaptation of lineages of different ploidy, none have followed the mutational dynamics in these evolving populations over time in detail [36,47–49].

In this study, we use experimental evolution to dissect the adaptive mechanisms underlying ethanol tolerance in yeast. Six isogenic *Saccharomyces cerevisiae* populations of different ploidy (haploid, diploid and tetraploid) were asexually propagated in a turbidostat over a two-year period, with ethanol levels gradually increasing during the experiment. This step-wise increase in exogenous ethanol levels resulted in a constant selective pressure for our cells. Whole-genome sequencing of evolved populations and isolated, ethanol-tolerant clones at different times during the experiment allowed us to paint a detailed picture of the mutational dynamics in the different populations. Several common themes in the type of adaptations and evolutionary mechanisms emerge, with all lines showing extensive clonal interference as well as copy number variations. Additionally, the haploid and tetraploid lines showed rapid convergence towards a diploid state. Despite these common themes, we find multiple lineage-specific adaptations, with little overlap in the mutated genes between the different populations. By applying a novel computational pipeline to identify affected pathways, we were able to reveal overlap between the functional modules affected in the different adapted populations, with both novel and previously established pathways and genes contributing to ethanol tolerance. Importantly, introduction of specific mutated alleles present in adapted populations into the ancestral strain significantly increased its ethanol tolerance, demonstrating the adaptive nature of these mutations and the potential of using experimental evolution to unravel and improve a complex phenotype.

## Results

### Evolved cells are more ethanol tolerant

**Fig 1:**
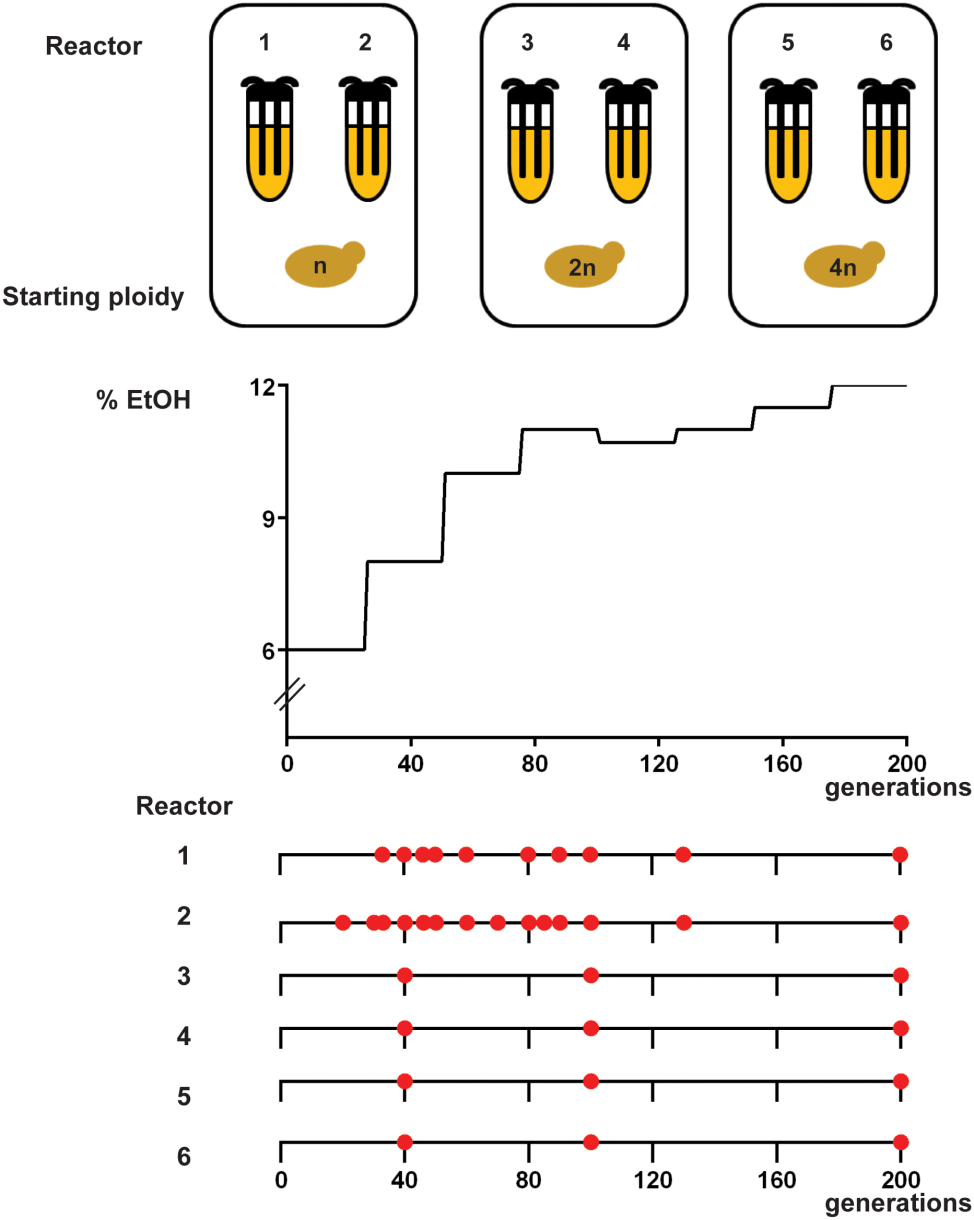
Experimental setup. (A) Experimental evolution of prototrophic, isogenic populations of different ploidy (haploid (VK111), diploid (VK145) and tetraploid (VK202)) for increased ethanol tolerance was performed in a turbidostat. Every 25 generations, the ethanol concentration in the media was increased in a stepwise manner (starting at 6% (v/v) and reaching 12% at 200 generations). Increasing the ethanol concentration from 10% to 11% dramatically reduced growth rate of evolving cells. Therefore, instead of increasing ethanol levels, we first reduced the ethanol level to 10.7% after 100 generations. (B) Red circles represent sampling points (indicated as number of generations) for which whole-genome sequencing was performed. For each circle, heterogeneous populations as well as three evolved, ethanol tolerant clones were sequenced. Sequencing of the population sample of reactor 4 at 200 generations failed, so this data is omitted from the manuscript. For generation 80 of reactor 1, only population data is available.

To study the mutational dynamics underlying increased ethanol tolerance, six prototrophic, isogenic *S*. *cerevisiae* strains of different ploidy (two haploid, two diploid and two tetraploid lines—with each line of the same initial ploidy started from the same preculture (VK111, VK145 and VK202 respectively, see also S4 Table) were subjected to increasing levels of ethanol. Specifically, ethanol levels were gradually increased from 6% (v/v) to 12% over a two-year period in a continuous turbidostat with glucose (4% (w/v)) as a carbon source. Samples were taken at regular time intervals and subjected to whole-genome sequencing analysis. Chloramphenical was added to the medium of our long-term evolution experiment to prevent bacterial growth. Chloramphenicol does not affect yeast growth (S1 Fig). In addition to sequencing each of the 6 evolving populations, we also sequenced the genomes of three clones isolated from each of the population samples, resulting in a total of 34 population samples and 102 clonal samples that were sequenced. Fig 1 depicts the experimental set-up as well as the time points (number of generations) for which whole-genome sequencing was performed.

**Fig 2:**
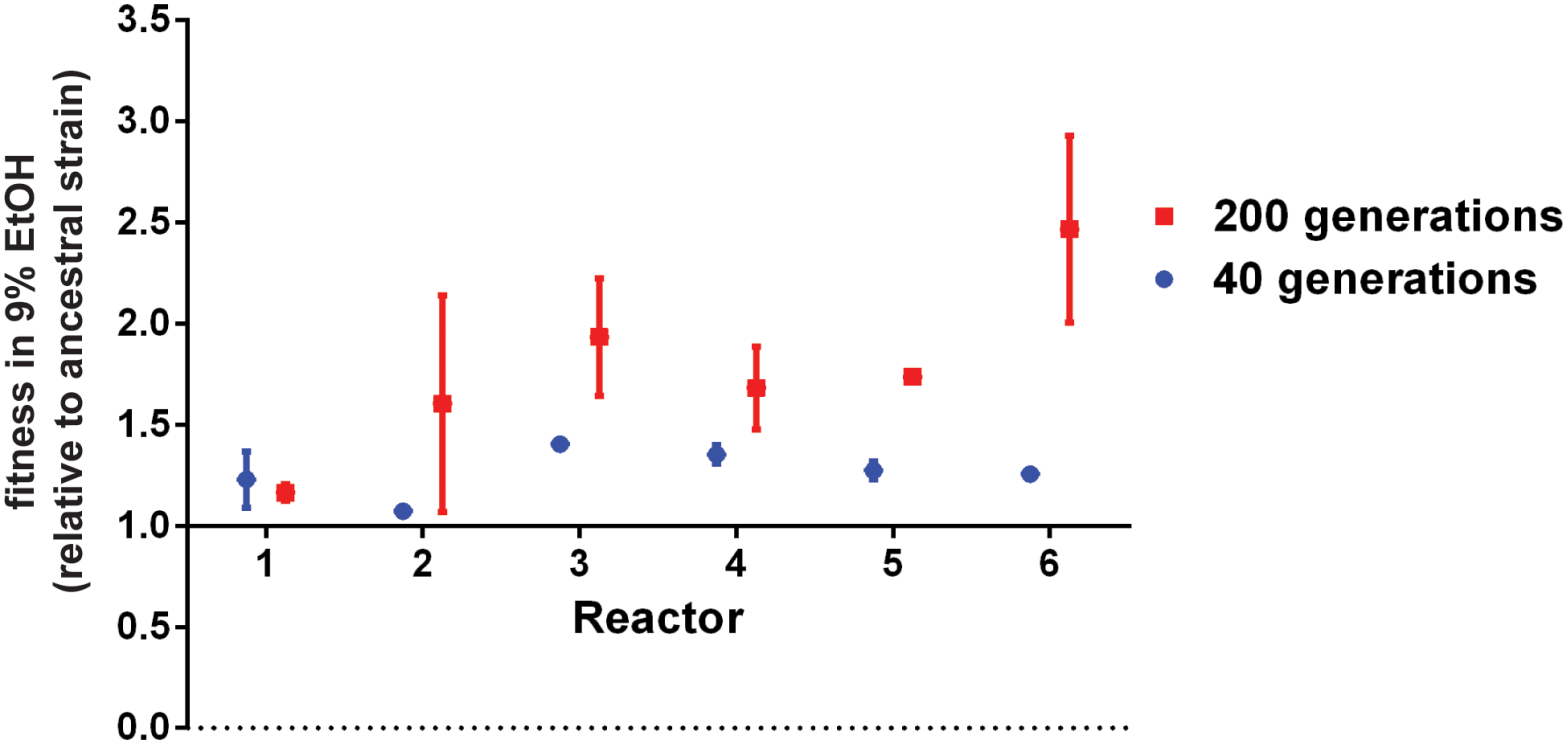
Evolved populations are more ethanol tolerant. Evolved populations from different reactors show increased fitness in EtOH. Fitness was determined for population samples of each reactor after 40 generations (blue) and 200 generations (red). Fitness is expressed relative to the ancestral strain of each reactor (haploid for reactor 1–2; diploid for reactor 3–4 and tetraploid for reactor 5–6). Data represent the average of three independent measurements, error bars represent standard deviation. Populations with mutator phenotypes showed higher standard deviations. This might be due to the large supply of mutations generated by these mutators, which could affect mutation-selection balance.

We determined the relative fitness of the evolved populations, isolated at 40 and 200 generations, and generally observed increases in fitness in high (9% v/v) EtOH (Fig 2 & Materials and Methods). In general, clones taken from the same population at the same time point had similar fitness; with some notable exceptions suggesting considerable heterogeneity within populations (S2 Fig). These fitness measurements were performed in 9% ethanol because the ancestral reference strains did not grow at higher ethanol levels. It is important to note that the actual increase in fitness of our evolved strains in higher ethanol levels (above 9% (v/v)) is much larger than what can be appreciated from Fig 2. Spotting of our evolved lineages on agar plates with increasing levels of ethanol indicates that these adapted strains can grow on concentrations up to 11% (v/v) EtOH, with the haploid control ancestral strain showing almost no growth under these conditions (i.e. an infinite increase in fitness compared to the ancestral haploid strain) (S3 Fig). Ancestral diploid and tetraploid strains show growth on 11% EtOH, although they are still outperformed by their evolved clones under these conditions.

### Diploidization of haploid cells is a fast way of increasing ethanol tolerance

**Fig 3:**
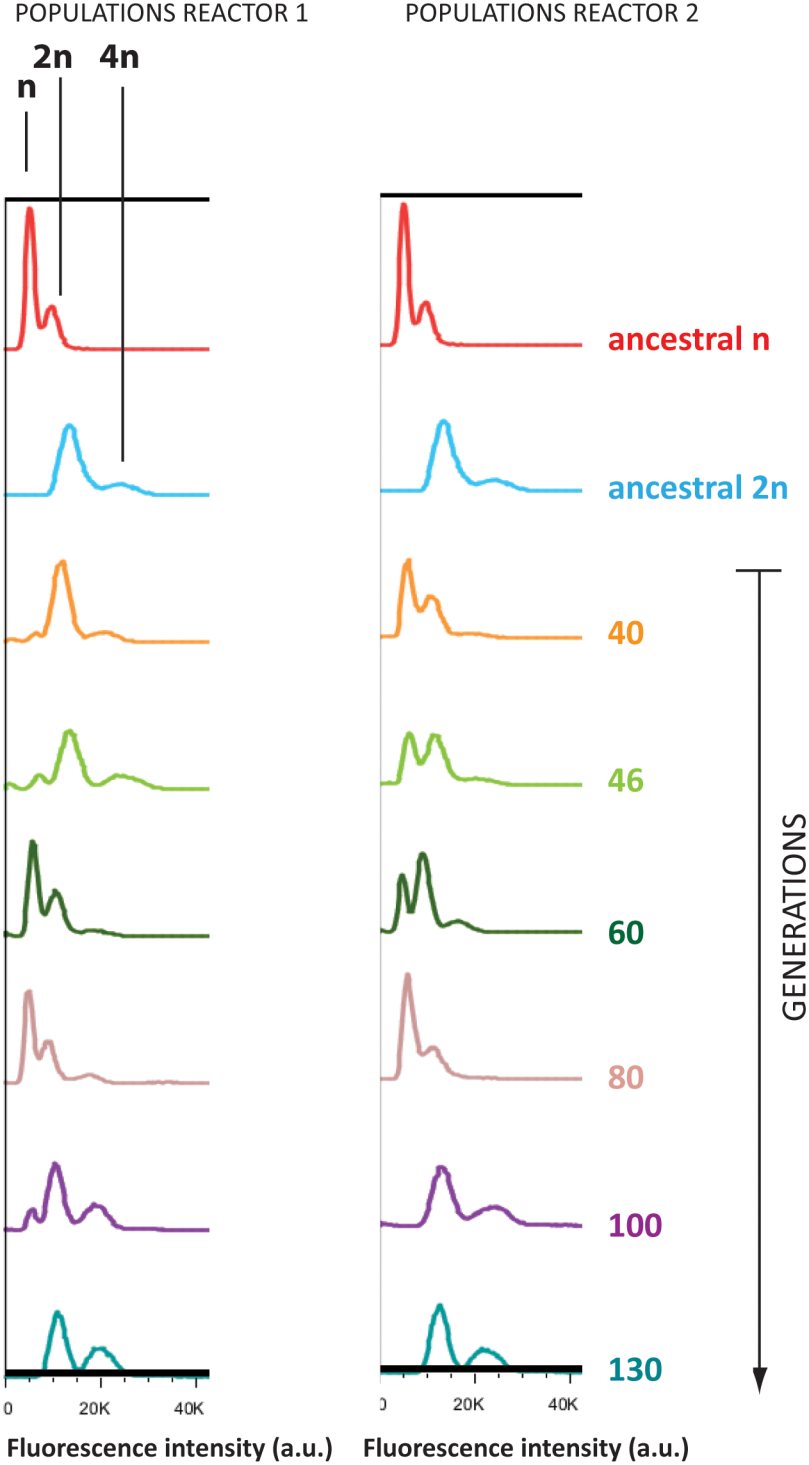
Haploid lineages diploidized during adaptation to EtOH. Flow cytometry analysis of DNA content (stained by propidium iodide) of evolved populations, compared to ancestral haploid (red) and diploid (blue). For the clonal ancestral samples, two peaks are observed, corresponding to the G1 and G2 phase of the cell cycle. Evolved populations sometimes display three peaks, indicative of both haploid and diploid subpopulations.

Propidium iodide staining and flow cytometry analysis of evolved populations showed that diploid cells appeared relatively quickly in the originally haploid populations (Fig 3; PI staining profiles of ancestral strains can be found in S4 Fig; ploidy of sequenced clones can be found in S1 File). This was observed independently in both reactors started from the same isogenic haploid population. It should be noted that around generation 40 in reactor 1, some diploids are already present in the originally haploid population. By generation 60, haploid cells took over the population again, possibly by acquiring a mutation that made them more fit than these new diploids. In both reactors, diploid cells have taken over the entire population by 130 generations. These diploid variants are still of mating type α, the same as the initial haploid ancestral strain, indicating that they did not arise through mating type switching and subsequent mating. Our results indicate that the diploids in our turbidostat are likely the result of failed cytokinesis. The relatively rapid evolutionary sweep of the new diploid variants points to a significant selective advantage. Competition experiments confirmed that the fitness of a diploid cell is indeed significantly higher than that of an isogenic haploid strain in 9% EtOH (p = 0.0063; unpaired t-test), whereas there is no significant fitness difference in the absence of ethanol (p = 0.469; unpaired t-test) (S5 Fig). Interestingly, our tetraploid starting populations also converged to a diploid state relatively fast during the evolution experiment. The parallel diploidization observed in two reactors with haploid ancestral cells and two reactors with tetraploid ancestral cells might suggest that becoming diploid is one of the main and/or one of the more easily accessible routes leading to increased ethanol tolerance.

### Whole-genome sequencing of evolved populations and clones reveals a complex pattern of adaptation

To identify the mutational pathways during adaptation to ethanol, we performed whole-genome sequencing of populations of evolving cells throughout the two-year experiment. Each population sample was sequenced to 500 fold coverage on average, and this for multiple time points (34 samples for all reactors combined, see Fig 1). In addition to this whole-population sequencing strategy, we also sequenced for each time point the genomes of three adapted clones to 80 fold coverage on average, resulting in 102 clonal samples sequenced in total. Details on the sequencing and analysis pipelines can be found in **Materials and Methods**.

### Whole genome sequencing of adapted clones reveals mutators and extensive aneuploidies

After 2 years, yielding around 200 generations, evolved clones (excluding clones from reactor 2 and 6, which acquired a mutator phenotype, see below) contained on average 23 SNPs compared to the ancestral strain (S1 File). This number is higher than what would be expected based on measured rates of spontaneous mutations [50], and could reflect an increased mutation rate under the stressful conditions imposed by the high ethanol levels in our set-up. Across all reactors and clones sequenced, we identified a total of 8932 different sites mutated. The largest fraction are SNPs (6424 out of 8932; 72%); Indels are found mostly in non-coding regions (1830 out of 2508; 73%), whereas SNPs are mostly found inside genes (4971 out of 6424; 77%). Most of these coding SNPs are non-synonymous (3672 out of 4971; 74%). A full list of all mutations (SNPs and Indels) in all sequenced clones for the different reactors and time points can be found in the S1 File.

**Fig 4:**
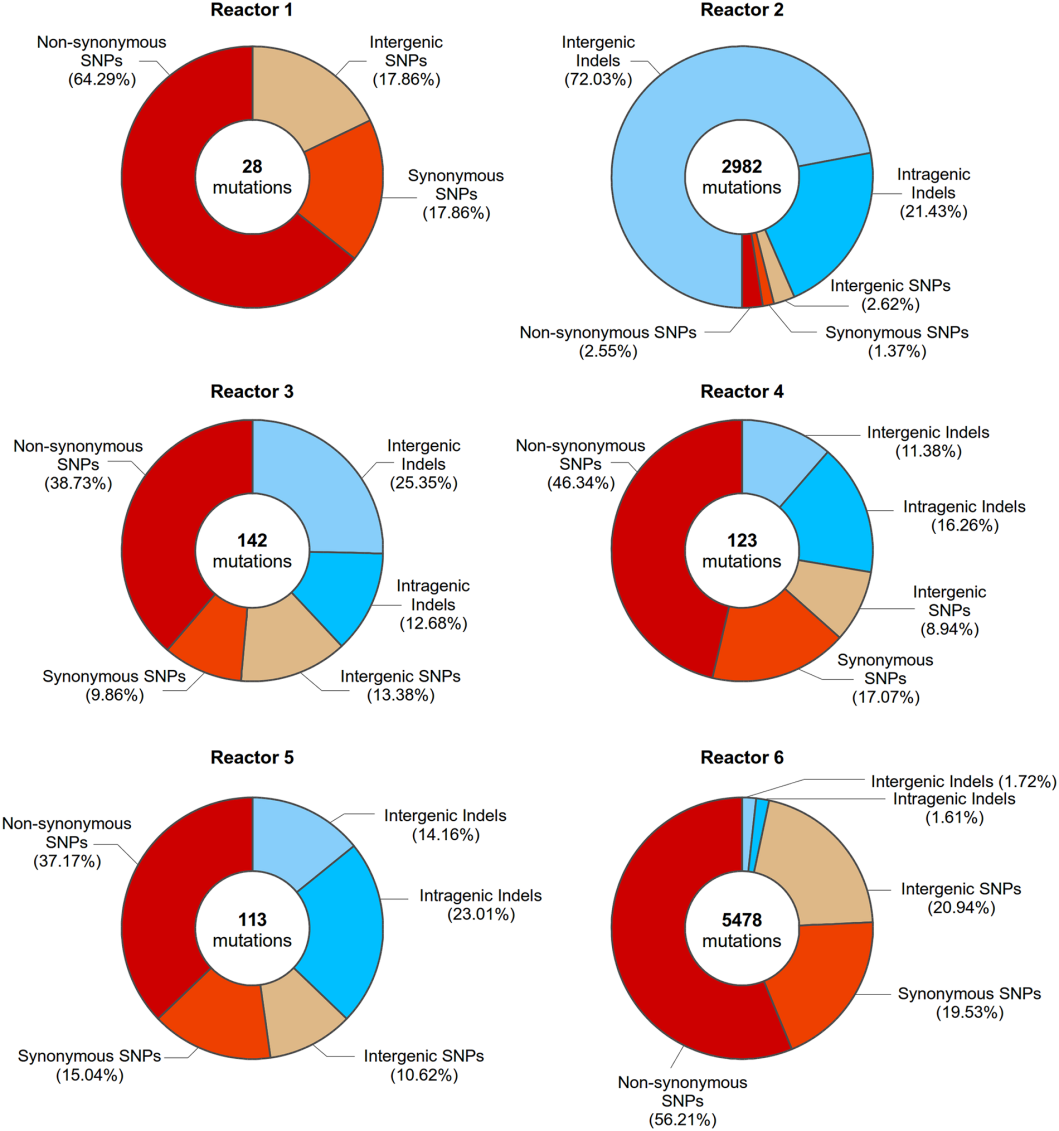
Mutations present in evolved clones isolated from all reactors at 200 generations. Circle diagrams depict the number and types of mutations identified in evolved clones at 200 generations of reactor 1, reactor 2, reactor 3, reactor 4, reactor 5 and reactor 6. Data for individual clones isolated at different time points can be found in S1 and S2 Files.

In two reactors (reactor 2 and reactor 6), we noticed a marked increase in the number of mutations found in individual clones (see Fig 4, S1 and S2 Files). Specifically, clones from reactor 2 contain a significantly higher number of indels (about 929 on average per genome for clones isolated after 200 generations, compared to 21 on average per genome for clones isolated after 200 generations in other reactors), whereas clones from reactor 6 display an increase in the number of SNPs (about 1765 per genome for clones isolated after 200 generations, compared to an average of 31 per clone for other reactors).This high number of mutations points to a so-called “mutator” phenotype typically present in cells with lower DNA replication fidelity and/or DNA repair. Interestingly, such mutators are frequently observed during evolution experiments, probably because mutators are more likely to acquire (combinations of) adaptive mutations faster [23,51–54].

Interestingly, the increased mutation rate in reactor 2 is first observed around generation 70–80, coinciding with the appearance of a 21 bp insertion in the *MSH2* gene– a key player in DNA mismatch repair. Mutations in *MSH2* have been previously reported to confer a mutator phenotype [55,56], and mutations in MutS, the ortholog of *MSH2* in *E*. *coli*, were identified in a long term evolution experiment and also result in a mutator phenotype [23]. Indeed, deletion of this gene, as well as re-creating this insertion in an otherwise wild type background drastically increases mutation rate (S6 Fig). The *MSH2* mutation eventually reaches a frequency of 100% at the end of the evolution experiment (200 generations). We currently do not know the exact cause of the elevated mutation rate observed in reactor 6.

**Fig 5:**
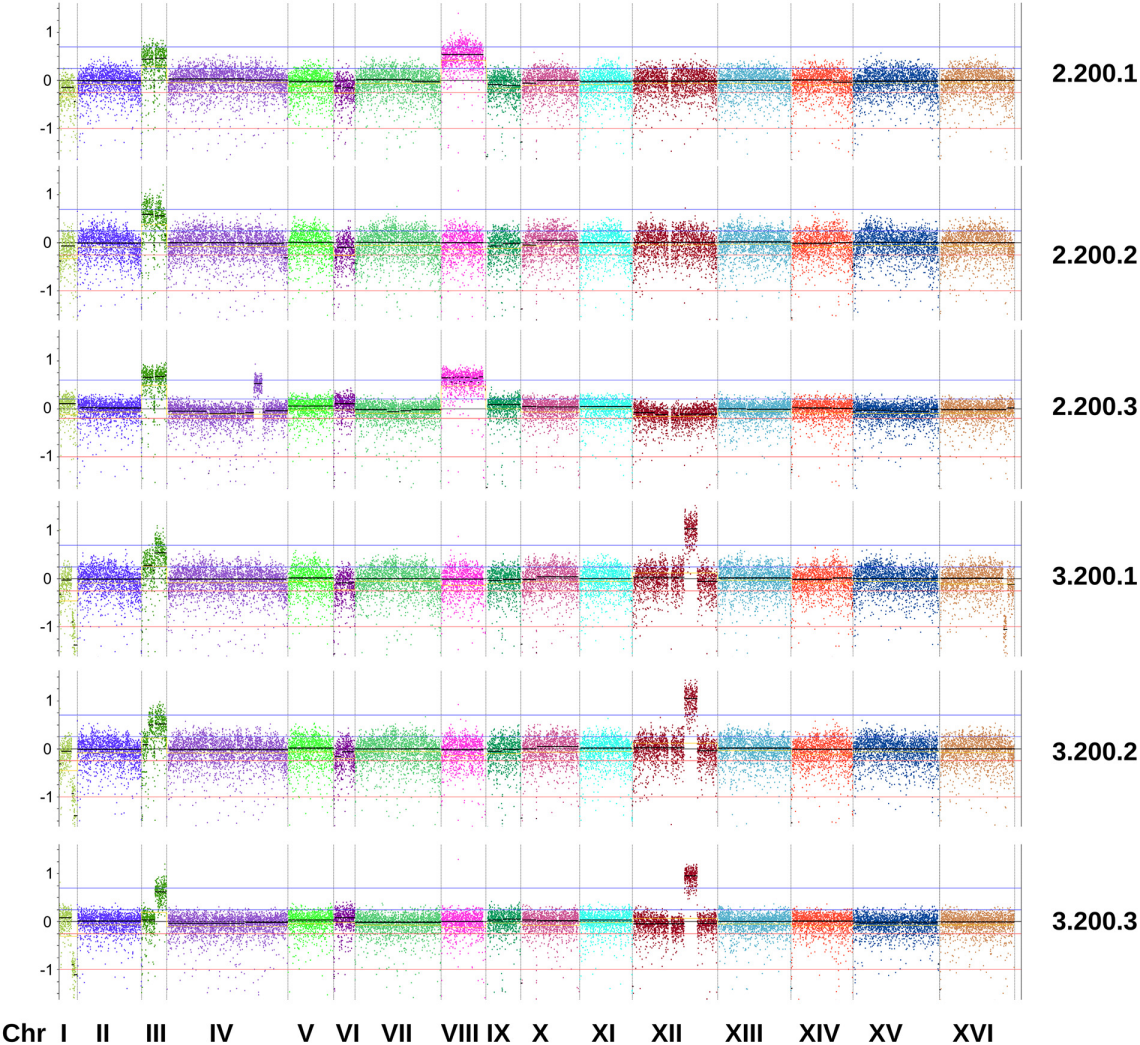
Copy number variation in evolved clones of reactor 2 and 3 isolated at 200 generations. Genome view of yeast chromosomes and CNV patterns from a sliding window analysis. The y-axis represents log2 ratios of the coverage observed across 500bp genomic windows to the coverage expected in a diploid genome without CNVs. Area of the plot located between the red lines (from -0.23 to -1) marks putative CNV loss events, whereas region between the blue lines (from 0.23 to 0.58) marks putative CNV gain events. Data for other reactors is shown in S3 File. It should be noted that the amplified region of chromosome XII observed in some of our clones does not correspond to the ribosomal DNA genes.

Apart from the convergence towards diploidy (see above), we also detected extensive copy number variation (CNVs) in our evolved clones, comprising both duplicated and deleted chromosomal regions (see Fig 5 and S3 File). Other studies have observed similar copy number variation during adaptation to stress, including heat stress and specific nutrient limitations [24,28,33]. Interestingly, acquisition of an extra copy of chromosome III appeared to be a common feature for most of our evolved clones (S3 File). Some (parts of) other chromosomes, including chromosome XII and chromosome IV, are also duplicated in several of our evolved clones. These frequent occurrences indicate that these specific aneuploidies could be adaptive under ethanol conditions, although the exact mechanistic basis remains to be elucidated and further experiments are needed to validate the (potential) adaptive role of these observed aneuploidies.

### Whole-genome sequencing of evolved populations reveals extensive clonal interference

While the sequencing of clones yielded valuable information on individual lineages within the evolving populations; sequencing of evolving populations yielded more information on the complex mutational paths and dynamics between sub-populations within each evolving population. A list of SNPs and Indels identified in the evolved populations, together with their allele frequency in the population at each sampling point, can be found in S4 File. We observe distinct pattern of mutations appearing and disappearing over time in each of the six reactors. Some of these mutations remain in the population, eventually reaching high levels or even complete fixation (i.e. presence in 100% of all cells in the population). Other mutations only persist for a short time, until lineages carrying these mutations are outcompeted by others, so-called clonal interference. In total, we identified 1637 mutations across all populations and time points. 117 of these mutations are no longer present in the final time points sequenced, and 101 mutations drop more than 10% in frequency after reaching their maximum frequency, indicative of clonal interference. Interestingly, we also identified some overlap between the mutations found in different independently evolving populations (i.e. in different reactors). Specifically, we find 20 genes that are mutated twice in different generations and populations, 3 genes mutated 3 times, 2 genes mutated 4 times, 2 genes mutated 5 times and 1 gene 6 times (see also S1 Table). This significantly differs from what would be expected by chance (see Materials and Methods and S1 Table for p-values, exact binomial test). Repeatedly hit genes are, amongst others, involved in stress response, cell cycle and heme biosynthesis.

**Fig 6:**
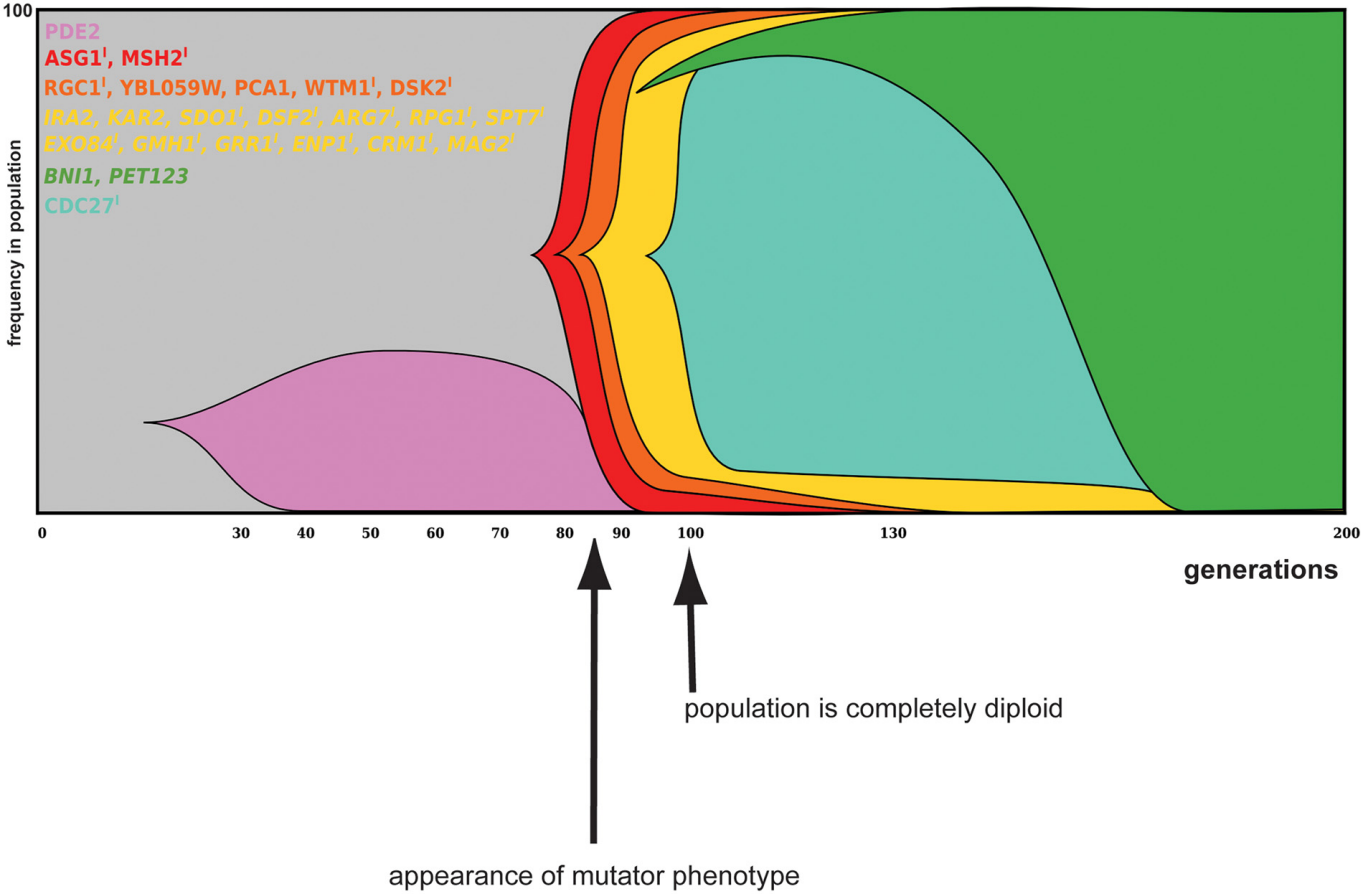
Dynamics and linkage of mutations in evolved populations of reactor 2. Mutations (reaching a frequency of least 20% in the evolved population samples) and corresponding frequencies were identified from population sequencing data. Muller diagram represents the hierarchical clustering of these mutations, with each color block representing a specific group of linked mutations. Indels are designated with ^I^, whereas heterozygous mutations are in italics. Mutations present as heterozygous mutations in all clones of a specific time point and present at a frequency of 50% in the population, are depicted as a frequency of 100% in the population, since it is expected that all cells in the population contain this mutation. After 80 generations, a mutator phenotype appeared in this reactor (indicated by arrow under graph), which coincides with the rise in frequency of an indel in the mismatch repair gene *MSH2*. Frequencies of haplotypes can be found in S2 Table. Dynamics and linkage for reactor 1 is shown in S7 Fig.

The higher number of sequenced samples from reactors 1 and 2 allowed us to further analyze these population sequences and group mutations based on correlations in the changes in their respective frequencies (based on the pipeline developed by [32]; see also Materials and Methods). This yields a more detailed picture of the different co-evolving sub-populations present in these reactors, which is depicted in the Muller diagrams of Fig 6 and S7 Fig, see also S2 Table for haplotype frequencies.

In both reactors, selective sweeps mostly consists of groups of mutations that move through the population together. While these reactors were inoculated with the same strain, the type and dynamics of mutations observed during adaptation appear very different. However, both evolving populations of reactor 1 and reactor 2 are characterized by strong clonal interference. In reactor 1, 4 different subpopulations are present around generation 90, each carrying different mutations. By generation 130, these lineages have been outcompeted by another lineage that almost completely dominates the population by 200 generations (S7 Fig). In reactor 2, a lineage carrying a mutation in *PDE2* (encoding a high-affinity cAMP phosphodiesterase) is driven to extinction by a subpopulation carrying indels in *ASG1* and *MSH2*. *ASG1* is a transcriptional regulator involved in the stress response and has been found mutated in evolved populations from different reactors (including reactor 1, see also Fig 6, S7 Fig and S4 File). Another example of clonal interference is observed in the later generations of this reactor: a subpopulation carrying a mutation in *CDC27* (encoding a subunit of the anaphase promoting complex/cyclosome) is driven to extinction by a subpopulation carrying mutations in *BNI1* (important for nucleation of actin filaments) and *PET123* (encoding a mitochondrial ribosomal protein).

### Diverse pathways involved in adaptation to ethanol

The results discussed above revealed extensive variability in the type and number of mutations present in each evolving population. While this could suggest the presence of several, different mutational pathways (and thus lack of parallel evolution), mutations in different genes might affect identical or similar pathways, implying that the physiological adaptation to high ethanol might in fact be more similar than what is immediately apparent from the individual mutations. It should also be noted that some of the mutations identified in our evolved lineages might represent adaptations to the device used, or other aspects of the selection regime, rather than ethanol per se.

To gain insight into the affected biological pathways and investigate the possible similarities in adaptation to increased ethanol, different computational approaches were used (see also S1 Text). First, a term-enrichment analysis was performed (for enriched clusters, see Table 1 and S5 File). For this analyses, we excluded mutations obtained from the populations with a mutator phenotype (reactor 2 and 6) because of their low signal to noise ratio. These enrichment methods have been used as one of the standard functional analysis tools and gave us a first insight into potential adaptive pathways present in our evolved lineages.

**Table 1:** Top functional clusters identified amongst genes hit by mutations in the evolution experiment. An enrichment score cutoff value of 1.3, equivalent to a p-value of 0.05 for term enrichment was used [57].

Cluster ranking	Cluster name	Enrichment score
1	protoporphyrinogen metabolism	1.71
2	Cell cycle	1.39

**Fig 7:**
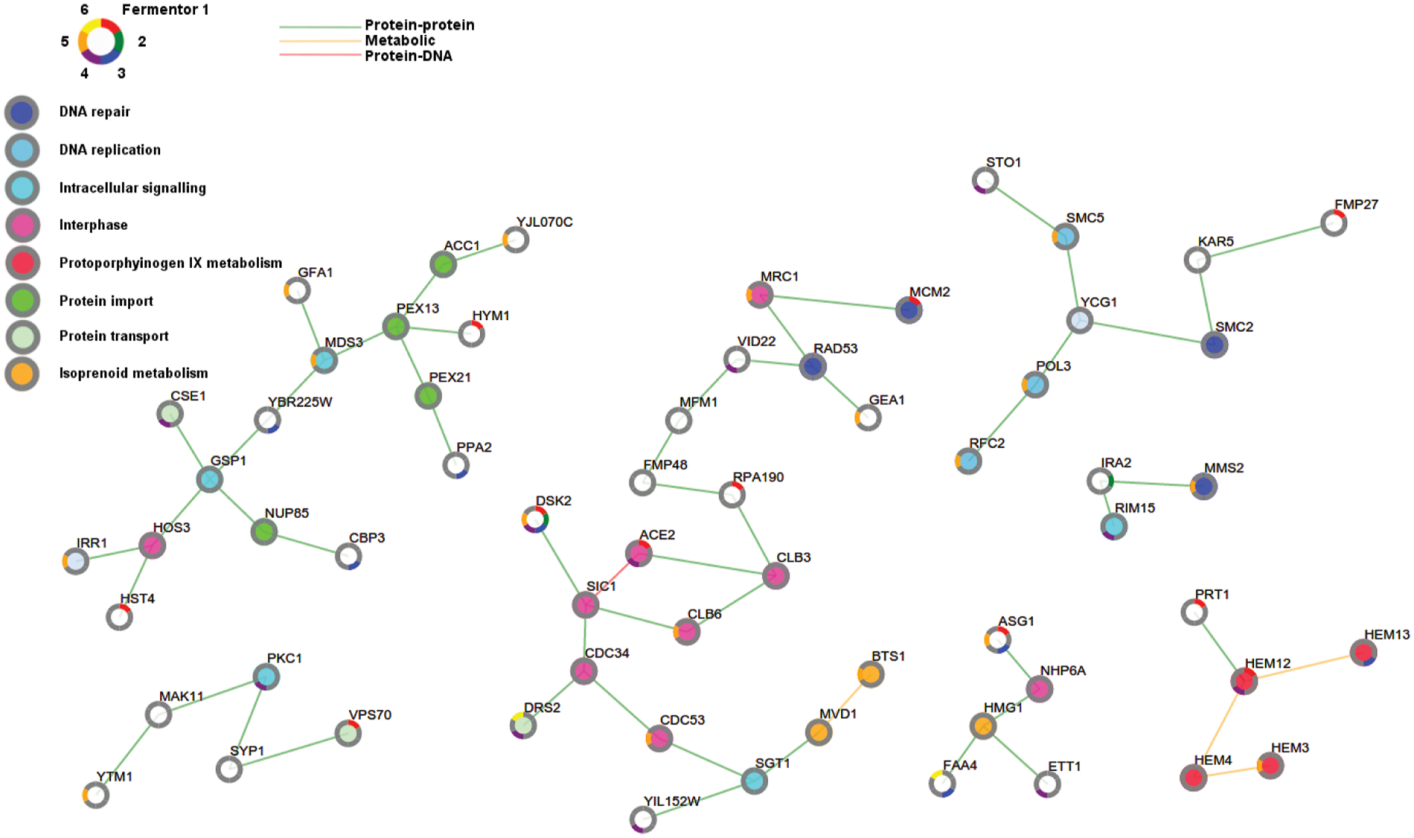
Adaptive pathways are involved in cell cycle, DNA repair and protoporphyrinogen metabolism. Shown is the sub-network that prioritizes putative adaptive mutations by applying PheNetic on all selected mutations, excluding those originating from the populations with a mutator phenotype i.e. reactor 2 and 6. The nodes in the network correspond to genes and/or their associated gene products. Node borders are colored according to the reactors containing the populations in which these genes were mutated. Nodes are colored according to gene function, for each gene the most enriched term is visualized (grey indicates no enrichment). Cell cycle related processes have been subdivided into DNA replication and interphase. The edge colors indicate different interaction types. Orange lines represent metabolic interactions, green lines represent protein-protein interactions, red lines represent protein-DNA interactions. Sub-networks extracted by separately analyzing the mutated genes observed in each of the different populations (reactors) are shown in S8–S13 Figs.

In a second step, we used a sub-network-based selection method [58,59] (see Materials and Methods) first developed for *E*. *coli* expression data. Here, we adapted and extended this method to select the subnetwork from the global yeast interaction network that best connected the mutated genes in the most parsimonious way. This method also identifies the intermediary genes involved in signaling mechanisms, which are not necessarily mutated in our evolved lineages but mediate the cellular response. We excluded mutations obtained from the populations with a mutator phenotype (reactor 2 and 6) because of their low signal to noise ratio. This analysis identifies genes frequently mutated in the different populations (*DSK2*, *ASG1*), as well as genes that are closely connected on the interaction graph (*HEM3*, *HEM12*,…). This latter set reflects parallelism at the pathway level in the different reactors. 25% of genes from inside our identified networks have been previously linked to ethanol tolerance (broadly defined as fermentation/growth capacity under conditions with EtOH, as reported in literature), whereas such as a connection was found for only 9% of genes outside of the enriched networks. From Fig 7, it is clear that sometimes multiple mutations occurring in the same pathway originated in the same reactor rather than across different reactors. This could indicate not only a level of parallelism between the different reactors but also between the sub-populations in the same reactor. To confirm this, we also applied the sub-network selection method on the mutations obtained for each of the populations separately. This confirms that within single populations the same pathways seem to be affected as those that are consistently affected between the populations (including cell cycle, DNA repair and protoporphyrinogen metabolism, see also below). All resulting sub-networks as well as the enriched genes (including p-values) can be found in S6 File, Fig 7 and S8–S13 Figs.

To increase mechanistic insight, we performed several additional analyses, such as interactome analyses of genes containing non-synonymous mutations in our different population samples as well as analyses of the effect of these mutations on protein functional domains (see also S1 Text, S3 Table and S7 File for more details). Together, these analyses helped us identify the different pathways that were affected in our evolved lineages. Pathways identified as affected across different reactors by these different analyses are discussed in more detail in the next paragraphs.

#### Cell cycle and DNA replication

Our analyses suggest that the cell cycle and DNA replication are affected in our evolved lineages (Fig 7). Ethanol has been previously reported to delay cell cycle progression, probably through depolarization of the actin cytoskeleton [60]. This could be relevant for adaptation in the sense that slower growth could protect cells from stress, perhaps by inducing genes involved in the general stress response [61–63].

#### Respiration

PheNetic analysis shows that protoporphyrinogen metabolism is affected in our evolved populations (Fig 7). Since this is important for heme biosynthesis, this could indicate that respiration is affected downstream of mutations. Non-synonymous SNPs in functional domains or modified sites are expected to have more effect on the cell than mutations outside of these regions. We find such mutations in proteins involved in regulation and in enzymes of heme biosynthesis and proteins that need heme groups for proper functioning, that are themselves involved in respiration (S3 Table and S7 File). These analyses point to a role for changes in respiration in adaptation to ethanol.

Together, our results show evidence for parallel evolution, with the same processes (cell cycle and DNA replication) affected by different mutations, and also highlight the plethora of processes involved in increased ethanol tolerance in our adapted lineages.

### Several high-frequency mutations increase ethanol tolerance of non-evolved strain

The high number of mutations (both SNPs and Indels) precluded an exhaustive analysis of all mutations present in our tolerant clones and populations and their effect on ethanol tolerance. One commonly used approach to investigate putative beneficial mutations is backcrossing of evolved clones to their ancestor, which does not contain any mutation. However, since each of the evolved populations proved unable to form any spores, this strategy was not accessible to us. Hence, we focused on SNPs reaching high frequencies in our 200 generation population samples. In total, nine SNPs were selected for further study (Table 2). Several of the mutated genes belong to or are linked to the processes affected across and/or within specific reactors (heme metabolism, protein transport and cell cycle, see also Table 2).

**Table 2:** SNPs in evolved lineages selected for introduction in non-ethanol tolerant strain. SNPs reaching high frequencies in the different evolved lineages were introduced in a haploid, non-ethanol tolerant strain. Gene functions were obtained from SGD, http://www.yeastgenome.org.

Location	SNP type	Nucleotide change	Function of mutated gene (SGD)	Enriched process involved in
***Reactor 1***				
ChrIV:1489310	Intergenic	A > T		NA
*HST4*	Non-synonymous	G262C	Histone deacetylase, involved in cell cycle progression, SCFA metabolism	Cell cycle*
*VPS70*	Non-synonymous	C595A	Vacuolar protein sorting, unknown function	Protein transport*
***Reactor 2***				
*YBL059W*	Non-synonymous	G479T	Unknown function	NA
*PCA1*	Non-synonymous	C1583T	P-type cation-transporting ATPase	Heme biosynthesis?* (potential role in iron homeostasis)
***Reactor 3***				
ChrXII:747403	Intergenic	C > T		NA
*HEM13*	Non-synonymous	G700C	Heme biosynthesis	Protoporphyrinogen metabolism/heme biosynthesis*
***Reactor 5***				
*PRT1*	Non-synonymous	A1384G	eIF3 subunit, essential for protein synthesis	NA
***Reactor 6***				
*MEX67*	Non-synonymous	G456A	Component of nuclear pore, mRNA export	NA

*Enriched process identified by Phenetic analyses across all reactors

**Fig 8:**
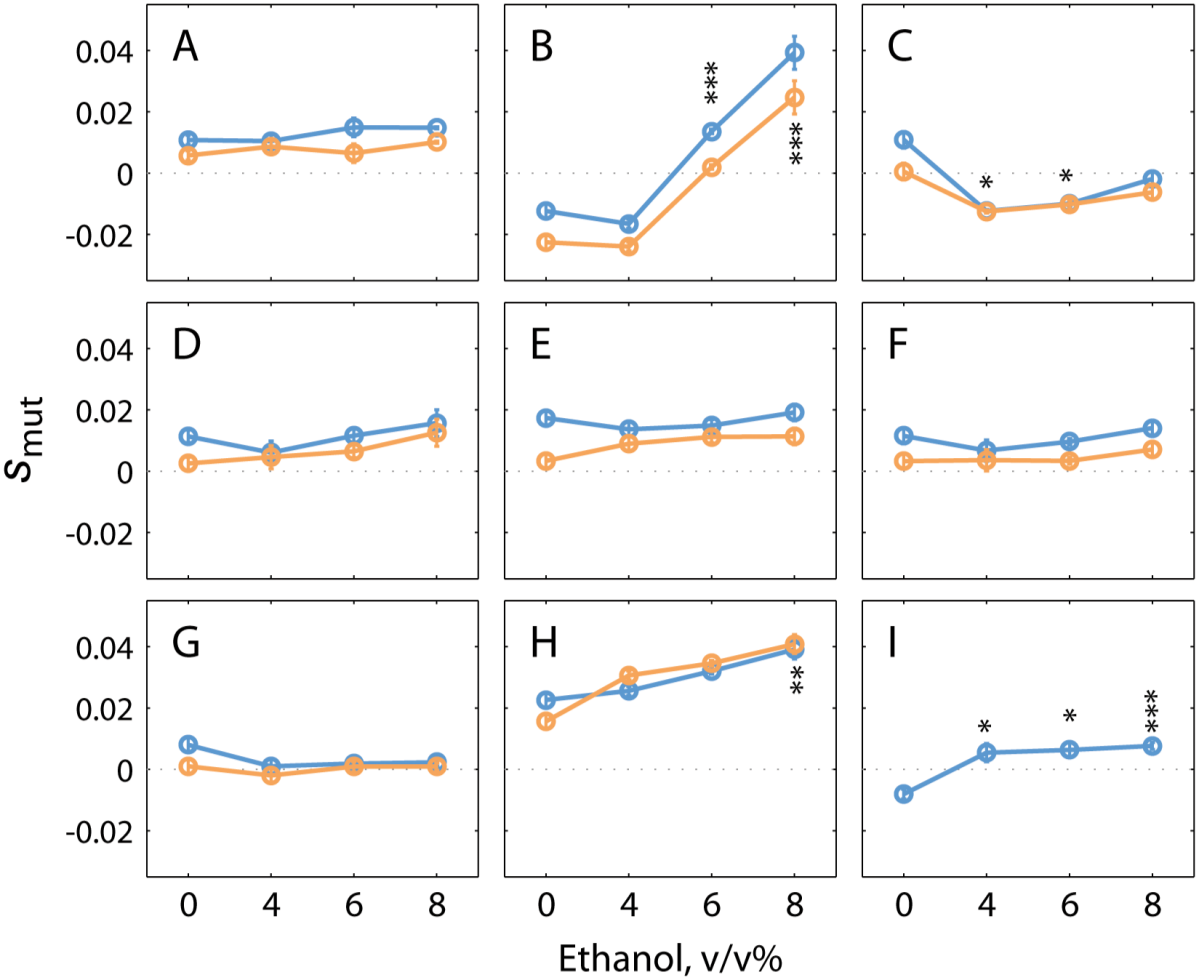
Single mutations present in evolved populations can increase ethanol tolerance of a non-adapted strain. Plots show the average selection coefficient (*s*_mut_) as a function of ethanol concentration for (**A**) *pca1* ^*C1583T*^, (**B**) *prt1* ^*A1384G*^, (**C**) *ybl059w* ^*G479T*^, (**D**) *intergenic ChrIV A1489310T*, (**E**) *hem13* ^*G700C*^, (**F**) *intergenic ChrXII C747403T*, (**G**) *hst4* ^*G262C*^, (**H**) *vps70* ^*C595A*^, and (**I**) *mex67* ^*G456A*^. Superscripts denote the exact nucleotide change in each of the mutants tested. YECitrine-tagged mutants were competed with the mCherry-tagged parental strain (orange dots); dye-swap experiments were carried out by competing the mCherry-tagged mutants with the YECitrine parental strain (blue dots), except for (**I**). Error bars show the S.E.M. from three experimental replicates. Asterisk show P-values from the one-way ANOVA tests of the mean differences in 4–8% ethanol compared to fitness in 0% ethanol: * *p* < 0.05; ** *p* < 0.01; ***p < 0.005. P values can be found in S7 Table.

These nine SNPs were subsequently introduced into the ancestral haploid strain. The effect of these mutations was assessed by high-throughput competition experiments [64], in 0, 4, 6 and 8% (v/v) EtOH conditions, with glucose as a carbon source (Fig 8, S14 and S15 Figs and S6 Table). Several mutants show a clear increase in fitness, with the fitness effect often depending on the concentration of ethanol in the medium. Most mutants show slightly increased fitness in medium without added ethanol, with further increases in relative fitness with increasing exogeneous ethanol concentrations. Mutants in *PRT1* and *MEX67* are less fit than the WT in non-ethanol conditions, but show increased fitness in higher ethanol levels. *MEX67* is a poly (A) RNA binding protein involved in nuclear mRNA export. *PRT1* encodes the eIF3b subunit of the eukaryotic translation initiation factor 3 (eIF3). The increased ethanol tolerance associated with these mutations hints at translation processes as targets of ethanol. Interestingly, a recent study in *E*. *coli* showed that ethanol negatively impacts transcription as well as translation [6]. However, if and how mutations in *MEX67* and *PRT1* might mitigate this effect and contribute to ethanol tolerance remains unknown.

Of all mutations introduced into the ancestral strain, *vps70* ^*C595A*^ provided the largest fitness increase. *VPS70* is putatively involved in sorting of vacuolar carboxypeptidase Y to the vacuole [65]. A mutation in *VPS70* has been recently identified by members of our team as a determinant of ethanol tolerance in a Brazilian bioethanol strain [66]. Interestingly, the *VPS70* mutation in our evolved ethanol-tolerant lineages alters the same amino acid as the mutation present in the industrial ethanol tolerant strain (Goovaerts A. and Thevelein J.M., personal communication). The fact that one of the mutations identified in our evolved lineages was also found in an industrially used bio-ethanol strain underscores the potential of our approach to find biologically relevant mutations for increased ethanol tolerance. Other members of the *VPS* family have been previously implicated in ethanol tolerance as well, but also here the exact molecular mechanism through which they could increase ethanol tolerance is still unclear [10,12,67]. To our knowledge, none of the other genes investigated in this study have been previously implicated in ethanol tolerance nor have these mutations been found in natural or industrial yeasts so far. This implies that these mutations could be prime candidates to improve the ethanol tolerance and production of existing industrial yeasts [68].

## Discussion

Many fundamental questions on the dynamics and genetics of adaptation to a complex and severe stress such as ethanol are still unanswered. Which processes contribute to ethanol tolerance? Which type and number of mutations are needed and/or sufficient, and how do they penetrate and fix within populations? To what extent are these mutations and the pathways they affect predictable? To address these questions, we performed high-coverage whole-genome sequencing of clones and populations isolated throughout a two-year evolution experiment and characterized their adaptation to increasing ethanol levels. This allowed us to paint a detailed picture of the mutational dynamics in our evolving lineages.

Our study demonstrates how many different evolutionary mechanisms all come together to provide adaptation to a severe and complex stress. Specifically, we find that adaptation to high ethanol levels involves changes in ploidy, copy number variation and the appearance of mutator phenotypes, with evolving populations showing strong clonal interference. Although these mechanisms have been observed in other studies (see, for example, [24,27,32,69–71], different mechanisms are often observed and/or studied separately. More importantly, in traditional evolution studies, populations are exposed to a fixed concentration of antibiotic or limiting nutrient. Under these conditions, selection pressure is reduced or even eliminated when cells become resistant. In our experimental set-up, ethanol levels were stepwise increased over time so that cells were evolving under constant selection. High concentrations of ethanol also actively kill non-tolerant cells, so that cells not adapted to the higher ethanol concentrations die and/or are washed out of the turbidostat. Taken together, our study combines aspects of traditional evolution studies with principles used in morbidostat experiments [27]. This set-up is likely a better representation of what happens when a population gradually penetrates and adapts to a new niche. Under such severe and continuous stress conditions (near-morbidostats), many cells fail to survive. This also implies that the traditional way of measuring evolutionary time by counting the number of generations may become problematic as cell death and mutations contributing to survival instead of replication become increasingly important. This phenomenon can also result in extremely quick sweeps, because a mutation cannot only penetrate a population because it increases the replication rate, but also because it protects against death. Moreover, as cell replication slows down, the number of mutations that is not associated with DNA replication but rather with DNA damage and repair likely increases. It seems plausible that these mechanisms contribute to the very quick genetic sweeps that we observe in some reactors, most notably the quick sweep of diploidized cells in reactors 1 and 2. This could also explain why the number of mutations that we observe in our two-year experiment might seem high for only 200 generations when compared to experiments where cells are growing at higher rates and where 200 generations are often reached within only one or two months of evolution. While it is difficult to think of a better universal measure of evolutionary time, it is important to realize that in some cases, a combination of physical time and generations may be a more appropriate way to assess evolution.

We find extensive variation in genome size in our evolved cells; with aneuploidies in a large number of our evolved clones as well as convergence toward a diploid state in our initially haploid and tetraploid populations. Becoming diploid thus appears to be a frequently used strategy by cells of different ploidy which effectively increases ethanol tolerance. Convergence to diploidy has been observed in other laboratory evolution experiments [25,36,72]. In contrast to these studies, we could demonstrate a clear fitness advantage of our diploid strains in ethanol. Although further research is needed to fully understand this fitness advantage, changes in gene expression and/or cell size could be important factors contributing to the increased fitness of diploid cells [41,73,74]. Unfortunately, this convergent evolution prevented us from performing a detailed analysis on the difference in adaptive strategies employed by haploids, diploids and tetraploids to increase ethanol tolerance.

Aneuploidy and copy-number variation are increasingly recognized as common themes in rapid adaptation [24,28,69,70]. It is believed that changes in the copy number of chromosomes or chromosomal fragments provide a relatively easily accessible way to change expression levels of specific key genes [75,76], and these CNVs can provide large, usually condition-specific, fitness effects [77]. However, such large-scale changes in the genome likely also have some unwanted, detrimental side effects, such as imbalance between gene products and genome destabilization [78–80]. Evolving populations are believed to gradually replace adaptive CNVs with more specific mutations that show fewer pleiotropic effects [28]. Notably, several of our evolved clones, isolated from different reactors, carry an extra copy of chromosome III and/or ChrXII; pointing towards a potential adaptive benefit of this specific aneuploidy. Interestingly, we find that clones isolated at later time points have a specific, smaller region of chromosome XII amplified (see also S3 File); indicating a more refined solution. GO enrichment and network analyses of the repeatedly amplified region of ChrXII (position 657500–818000) hints at cell wall formation as one of the key processes affected by these amplifications. Previous studies have indeed shown that cell wall stability is a key factor involved in ethanol tolerance [18].

Apart from diploidization of our evolving lineages, another example of parallelism at the phenotypic level is the appearance of a mutator phenotype in two of our six evolving populations. The sweep of the *MSH2* mutation in reactor 2 is likely caused by a so-called hitchhiking event, with the high mutation rates in the *MSH2* mutant leading to the appearance of one or several beneficial mutations that drive the selective sweep.

Parallelism at the genotypic level is less clear: we find few mutations and mutated genes shared between the different evolved lineages. Interestingly, nine (*ACE2*, *POL3*, *PUF4*, *GFA1*, *UTH1*, *JID1*, *RIM15*, *ATG11* and *VPS74*) out of the 28 genes (32%) that were found hit in multiple reactors have been previously implicated in ethanol tolerance. Applying different types of network and enrichment analyses revealed functional modules affected in several of the adapted populations. These pathways include response to stress, intracellular signal transduction, cell cycle and pathways related to membrane composition and organization (such as isoprenoid metabolism, glycerophospholipid catabolism and fatty-acyl-coA metabolism). For some of these pathways, further work is needed to clarify their exact involvement in ethanol tolerance.

To investigate the phenotypic effect of mutations present in our evolved lineages, we performed high-throughput fitness measurements in different ethanol concentrations. While our evolved lineages contained multiple mutations, single mutations reaching high frequency in the evolved populations could already significantly increase ethanol tolerance when introduced into the ancestral, non-evolved haploid strain. Moreover, several of these single mutations selected for further study also showed a (modest) fitness benefit in conditions with no ethanol, with greater increases as ethanol levels rise. The number of mutations identified in this study was too large to investigate the fitness effect of each individual mutation. However, our strategy to select mutations that reached fixation in the evolved populations clearly proved successful, identifying as many as 4 mutations (out of 9 tested) that confer a fitness advantage in high ethanol environments. Mutations in genes linked to processes identified as affected across our different reactors—such as protein transport (*VPS70*)–indeed increased fitness in EtOH. This underscores the potential of PheNetic, a sub-network based selection method for identifying adaptive mutations. Five of the mutations tested did not significantly increase fitness, although they reached high frequency in our adapted populations. This is indicative of hitch-hiking with other, beneficial mutations; or possible epistatic interactions with other mutations.

Why then would not all feral yeasts show high ethanol tolerance, if it appears so easy to attain? Firstly, it seems plausible that not all yeasts are confronted with selection for high ethanol tolerance. Furthermore, it is important to note that we have not tested the fitness of the mutants under many different conditions that mimic the natural habitats of yeasts. It seems likely that some of the mutations identified in this study would result in lower fitness in other environments [81,82]. Moreover, we have not investigated the effect of combined mutations. While it is possible that combining different mutations could increase ethanol tolerance even further, it also seems likely that some mutations and/or specific combinations of mutations could lead to reduced fitness in different low and/or high ethanol environments. Indeed, while our clones have increased fitness in EtOH, we observe that fitness of several of our evolved clones (containing multiple other mutations apart from the ones investigated in this study) decreased in medium without exogenous EtOH (see S16 Fig). These results are indicative of antagonistic pleiotropy: the specific mutations present in our evolved clones increase fitness in one condition (high ethanol, which was selected for), whereas they reduce fitness in other environments.

Ethanol resistance is an important trait for the survival of feral yeasts in nature because the ethanol produced inhibits growth of competing microorganisms, while it serves as a carbon source in later stages of growth, when all fermentable sugars are depleted (the so-called make-accumulate-consume strategy [83–85]). Our results suggest that adaptation to high ethanol is complex and can be reached through different mutational pathways. Apart from yielding insight into the evolutionary mechanisms leading to such complex and ecologically important phenotypes, our study is also of considerable industrial importance. Several of the mutations identified in this study may be useful to increase the ethanol tolerance of industrial strains used for the production of alcoholic beverages or biofuels.

## Materials and Methods

### Strains used in this study

Starting strains for the evolution experiment are all derived from the haploid prototrophic S288c strain FY5 [86]. To prevent clumping of cells during the evolution experiment, the flocculation genes *FLO1*, *FLO10* and *FLO11* were deleted in this strain using deletion cassettes based on pUG6, conferring resistance to G-418 disulfate [87]. Markers were removed through the Cre/LoxP technique using pSH65 [88]. Mating type switching of this strain was then performed, using plasmid pSB283, to create isogenic diploid and tetraploid strains. Fluorescent versions of strains (YECitrine or mCherry tagged) were constructed by integrating fluorescent markers at an intergenic, neutral region of chromosome II. A full list of strains used, with their complete genotype, can be found in S4 Table. Primers used for strain construction and verification can be found in S5 Table.

### Long-term selection

Populations were founded in 400 mL ethanol containing media. Media contained 10 g/L yeast extract, 20g/L bactopeptone, 4% (w/v) glucose, 0.001% (v/v) Rhodorsil, Antifoam Silicone 46R, chloramphenicol (50 μg/mL) and increasing concentrations of ethanol. Populations were maintained at an average population size of 10^10^ cells. After 25 generations, the level of EtOH in the media was increased each time (starting at 6% (v/v) and reaching 12% at 200 generations).

Turbidostat cultures were maintained using Sixfors reactors (Infors) at 30°C, pH was kept constant at 5.0 with continuous mixing at 250 rpm in aerobic conditions. At regular times, a population sample was obtained from each of the cultures for further analyses and stored in glycerol at -80°C. For DNA extraction purposes, a population cell pellet was also frozen down at -80°C.

### Fitness determination

Fitness for all evolved strains was determined in rich medium (YP, 2% (w/v) glucose) with 9% (v/v) ethanol, by competing strains against a YECitrine labeled ancestral strain. Cultures were pre-grown in YPD 6% ethanol. After 12h, wells of a 96 deep well plate were inoculated with equal numbers of labeled reference and unlabeled strains (~ 10^6^ cells of each) and allowed to grow for around 10 generations. Outer wells only contained medium and acted as a buffer to prevent ethanol evaporation. Additionally, plates were closed with an adhesive seal and plastic lid, and parafilm was used to prevent ethanol evaporation. Cultures were regularly transferred to new medium to prevent nutrient depletion. The ratio of the two competitors was quantified at the initial and final time points by flow cytometry. Data analysis was done in FlowJo version 10. Measurements were corrected for the small percentage of labeled, non-fluorescent cells that occurred even when the reference strain was cultured separately as well as for the cost of YFP expression in the labeled reference strain. For each fitness measurement, three independent replicates were performed. The selective advantage, s, of each strain was calculated as s = (ln(U_f_/R_f_)-ln (U_i_/R_i_))/T where U and R are the numbers of unlabeled and reference strain respectively, the subscripts refer to final and initial populations and T is the number of generations that reference cells have proliferated during the competition. The fitness of the unlabeled WT strain was designated 1, fitness of the evolved strains as 1+s.

### Determination of cell ploidy

DNA content of evolved populations and evolved clones was determined by staining cells with propidiumiodide (PI) and analyzing 50 000 cells by flow cytometry on a BD Influx. The ancestral haploid and diploid strains used in the evolution experiment were used for calibration.

### Whole genome sequencing

For evolved populations, genomic DNA was directly extracted from pellets that were frozen at the time of sample taking.

Evolved clones were selected from the different population samples by streaking glycerol stocks from the corresponding population samples on YPD plates. Swabs from each population were subsequently grown in YPD 6% EtOH and dilutions were plated on YPD plates with different ethanol concentrations (ranging from 8% to 10%; with a 0.5% stepwise increase in ethanol concentrations). From these plates, ethanol tolerant clones were selected and genomic DNA of these clones was extracted.

Genomic DNA was prepared using the Qiagen genomic tip kit. Final DNA concentrations were measured using Qubit. Paired-end sequencing libraries with a mean insert size of 500 bp were prepared and libraries were run on an Illumina HiSeq2000 (EMBL GeneCore facility, Heidelberg). Average sequencing coverage for clone and population sample is 80X and 500X respectively.

#### Variant calling, filtration and annotation

Adaptors removal and reads quality control were done by Trim Galore! (http://www.bioinformatics.babraham.ac.uk/projects/trim_galore/) with options (-q 30—length 50). The clean reads were then mapped reference *S*. *cerevisiae* genome (S288C, version genebank64) using Burrows Wheeler Alignment allowing maximum 50bp gaps [89]. To identify mutations we employed the BROAD Institute Genome Analysis Toolkit (GATK, version 3.1) [90]. We began by performing local realignment of reads around Indels, in order to eliminate false positives due to misalignment of reads, which was followed by a base recalibration step. We then performed SNP and Indel calling according to the GATK best practice recommendations. Population samples and single clone datasets were analyzed independently.

For population data, Indel calling was performed using the UnifiedGenotyper tool, and the identified variants were filtered based on the recommended criteria (QD < 2.0, FS > 200.0, ReadPosRankSum < -20 and InbreedingCoef < -0.8). For the SNPs, we used the UnifiedGenotyper to perform multi-sample SNP calling on all the population samples together. The resulting multi-sample callset was then used as a prior to call SNPs in all individual samples. The called SNPs were then subject to filtering (QD < 2.0, FS > 60, ReadPosRankSum < -8.0, MQ < 40, MQRankSum < -12.5). Furthermore, we filtered out SNPs that were called inside regions containing Indels. We also generated a list of all known repetitive regions and low complexity regions using the program RepeatMasker (http://www.repeatmasker.org/cgi-bin/WEBRepeatMasker), which were masked out from the assemblies. Finally, variants present in the ancestral strain were filtered out from all samples.

A similar approach was used to analyze the clonal samples. Ploidy level of each isolated clone was determined by PI staining and variant calling was performed with UnifiedGenotyper for haploid clones or HaplotypeCaller for diploid clones. Subsequently, SNPs and Indels were filtered based on the GATK best practice recommendations as mentioned above. The inbreeding coefficient filter was excluded in the clonal sample filtering for Indels, as it is a population-level metric. Annotation and effect prediction of all identified variants was performed using snpEff [91].

Final processing of the data was performed using a R script, which involved filtering out variants called within the sub-telomeric regions (15 kbps from the chromosome ends) and—for population samples—variants whose frequency did not change by more than 10% during the experiment. We used the infrastructure of the VSC—Flemish Supercomputer Center for these analyses. For improved performance, CPU multithreading (-nct) capabilities of GATK were utilized, providing up to 20 cores and maximum memory capacity of 64 GB per process.

#### Identification of Copy Number Variations (CNV)

CNVs in the samples were identified using Nexus Copy Number software, version 7.5 (http://www.biodiscovery.com/software/nexus-copy-number). Reads were accumulated in 500-base bins along all chromosomes, rejecting bins with less than 10 reads. Log2 ratios of copy numbers were then estimated from the read-depth counts in these intervals. We used the following calling parameters: minimum number of probes per segment of 5, significance threshold of p-value = 1 × 10^-9, percentage of removed outliers of 5% and the limits for copy gain and loss of+0.25 and -0.25, respectively.

### Statistical analyses of genes hit multiple times

The p-values probabilities of genes being hit by a mutation a specific number of times were calculated using the binomial distribution. The number of draws was set to the total number of mutations found inside coding regions (i.e. 817); the number of successes was set to the number of times a specific gene was hit by a mutation (2 to 6); the probability of success was set to the specific ratio of the length of each gene hit multiple times (in nucleotides) over the entire coding content of the *S*. *cerevisiae* genome (9080922 nucleotides).

### Haplotype reconstruction

Haplotype reconstruction was based on the approach described by [32]. Prior to the actual reconstruction, variants identified in the sequencing data were subject to further processing by excluding variants that were multi-allelic, variants that exhibited mixed zygosity in the isolated clones (i.e. homozygous in one clone and heterozygous in another clone) and variants that didn't reach the frequency of 0.2 at any point during the experiment. Haplotypes (or 'mutational cohorts') present in the remaining variants were next reconstructed using a Matlab script (kindly provided by the Desai lab) on a local machine running Matlab 2013a. Briefly, variants present in our dataset were clustered into haplotypes based on the Euclidean distance between their frequencies at specific time-points of the experiment. Afterward, frequencies of individual variants assigned to a specific haplotype were averaged to obtain the frequency of the haplotype itself. Frequencies of the identified haplotypes at specific time points (see S2 Table) were then used as source data to draw their approximate Muller diagram representations with Inkscape 0.48.4.

### Enrichment and network analysis

Functionally meaningful terms enriched in the list of genes hit in our evolution experiment were identified using DAVID Tools [57,92]. An enrichment score cutoff value of 1.3 was used, equivalent to a p-value of 0.05 for term enrichment, as recommended by the authors [57].

### Phenetic analyses

Intergenic mutations were discarded for the analysis. The interaction network used as input for PheNetic was composed of interactomics data obtained from KEGG [93] for metabolic interactions, String for protein-protein interactions [94] and Yeastract for protein-DNA interactions [95]. The total interaction network contains 6592 genes and 135266 interactions. This interaction network was converted to a probabilistic network using the distribution of the out-degrees of the terminal nodes of the network edges. By doing so, edges connecting nodes with a low out-degree will receive a high probability while edges connecting nodes with a high out-degree receive a low probability. Using lists of mutated genes as input, PheNetic will now infer that sub-network of the probabilistic network that best connects the mutated genes in the list over the probabilistic network. As the probabilistic network penalizes hub nodes, the inferred sub-network will therefore preferentially connect the mutated genes through the least connected parts of the network. This results in selecting the most specific parts of the network that can be associated with the mutated genes.

PheNetic was used to connect the mutated genes over the interaction network [58] with the following parameters: 100-best paths with a maximum path length of 4 were sampled between the different mutations in combination with a search tree cutoff of 0.01. As the size of the selected sub-network by PheNetic is dependent on both a cost parameter and the number of mutated genes in the input, different costs were used for the sub-network inference from different sizes of mutated gene lists. For the sub-network inference between all the mutated genes from the non-mutator reactors a cost of 0.25 was used, for the non-mutator reactors (1,3,4,5) a cost of 0.05 was used as they all have a similar amount of mutated genes, and for the mutator reactors (2 and 6) a cost of 0.5 was used. For more details, see S1 Text.

### Network visualization and enrichment

The resulting networks were visualized using Cytoscape and a functional enrichment using the biological process terms of Gene Ontology in combination with the annotation of SGD of the sub-networks was performed using the Bingo plugin, version 2.44 [96]. The resulting enrichment results are listed in S6 File.

### Construction of mutant strains

Selected mutations identified from the whole-genome sequencing data were introduced into the ancestral genetic background using the following protocol. First, a selectable marker conferring resistance to hygromycine was introduced near the genomic location of interest through homologous recombination. Part of this locus was then amplified together with the selectable marker using a forward primer containing the desired mutation. The resulting PCR product was then transformed into the ancestral strain; and presence of the mutation was verified by Sanger sequencing. Primers used can be found in S5 Table.

### High-throughput competitive fitness measurements

YECitrine- or mCherry-tagged site-directed mutant strains were competed with the parental mCherry or YECitrine tagged strains, respectively, as described [64]. In brief, saturated cultures of mutant and parental strains were mixed in equivalent volumes and inoculated onto 150 μl of YNB-low fluorescent medium in 96-well microtiter plates (Corning 3585). Micro-cultures grew without shaking and were serial-diluted every 24 hrs for approximately 28 generations (7 days) in a fully automated robotic system (Tecan Freedom EVO200) that integrates a plate carrousel (Liconic STX110), a plate reader (Tecan Infinite M1000), a 96-channel pipetting head, an orbital plate shaker, and a robotic manipulator arm. The equipment was maintained in an environmental room at constant temperature (30°C) and relative humidity (70%). Fluorescence signal (mCherry: Ex 587 nm/5 nm and Em 610 nm/5 nm; YECitrine: Ex 514 nm/5 nm and Em 529 nm/5 nm) and absorbance at 600 nm were monitored every hour during the entire experiment. The YECitrine- or mCherry-tagged parental strains were competed to each other for normalization and monitored individually to determine background fluorescence signal. Fluorescence and absorbance output data was analyzed in Matlab as described [64] to obtain an average selection coefficient, *s*_mut_, with its S.E.M. from three experimental replicates.

### Accession numbers

All sequencing data are available from the NCBI Sequence Read Archive: Bioproject, accession number PRJNA292495.

## Supporting Information

S1 Fig Chloramphenicol does not affect growth of ancestral strains. Tenfold serial dilutions of ancestral haploid, diploid and tetraploid strains (starting OD600nm = 1.0) were spotted on agar plates. (A) growth in YPD and YPD containing 50 μg/ml chloramphenicol after 48 hours. (B) growth in YPD 6% ethanol and YPD 6% ethanol containing 50 μg/ml chloramphenicol after 96 hours. (C) growth in YPD 8% ethanol and YPD 8% ethanol containing 50 μg/ml chloramphenicol after 96 hours.
(TIF)

S2 Fig Evolved clones are more ethanol tolerant. Evolved clones from different reactors show increased fitness in EtOH. Data represent means of three biological replicates, error bars represent standard deviations. Clones from the same time point (number of generations) are depicted in the same color. (A) Fitness of sequenced, evolved clones of reactor 1, determined in 9% EtOH. Fitness is expressed relative to the fitness of the haploid ancestral strain. Fitness of the ancestral, isogenic haploid and diploid strains is also depicted in this figure. A diploid strain is more fit than an isogenic haploid strain in 9% EtOH. (B) Fitness of sequenced, evolved clones of reactor 2, determined in 9% EtOH. Fitness is expressed relative to the fitness of the haploid ancestral strain. (C) Fitness of sequenced, evolved clones of reactor 3 (blue) and reactor 4 (red) after 200 generations, determined in 9% EtOH. Fitness is expressed relative to the fitness of the diploid ancestral strain. (D) Fitness of sequenced, evolved clones of reactor 5 (blue) and reactor 6 (red) after 200 generations, determined in 9% EtOH. Fitness is expressed relative to the fitness of the tetraploid ancestral strain. Fitness of one of the evolved clones of reactor 5 could not be determined.
(TIF)

S3 Fig Evolved clones are more ethanol tolerant than ancestral strains. Tenfold serial dilutions of evolved clones (starting OD_600nm_ = 1.0) isolated after 200 generations from the different reactors were spotted on YPD (2%) agar plates containing 0 (left panel) or 11% (right panel) EtOH (v/v) to assess their ethanol tolerance. Plates were carefully sealed with parafilm to prevent ethanol evaporation and incubated for 3 or 10 days at 30°C (for 0 and 11% EtOH respectively). Different strains were randomized for spotting on plates, the different panels shown are taken from the same plate. Evolved clones from reactor 2 do not grow in 11% EtOH on agar plates, although they show high fitness in 9% ethanol in liquid medium (S2 Fig). This could reflect differences in the mutations required to tolerate 9% vs 11% ethanol, and/or differences between growth on liquid and solid medium.
(TIF)

S4 Fig Flow cytometry analyses of DNA content of haploid, diploid and tetraploid strains. Flow cytometry analysis of DNA content (stained by propidium iodide) of ancestral haploid (red), diploid (blue) and tetraploid (orange) strains.
(TIF)

S5 Fig Isogenic diploids are more fit than haploids in 9% EtOH. Fitness of isogenic haploid, diploid and tetraploid strains was determined in 0 and 9% EtOH. Fitness is expressed relative to the fitness of the haploid strain under a specific condition. A diploid strain is significantly more fit than a haploid strain in 9% EtOH.
(TIF)

S6 Fig Mutations in the mismatch repair gene *MSH2* drastically increase mutation rate. *Msh2* ^*C1630 CACGTAATGACGCCAAGGAGTT*^ (labeled *msh2 indel* in figure) and *msh2Δ* strains show elevated mutation rate compared to wild type strain. Fluctuation assays were used to determine mutation rates; error bars represent 95% confidence intervals.
(TIF)

S7 Fig Dynamics and linkage of mutations in evolved populations of reactor 1. Mutations (reaching a frequency of at least 20%) and corresponding frequencies were identified from population sequencing data. Muller diagram represents the hierarchical clustering of these mutations, with each color block representing a specific group of linked mutations. Indels are designated with ^I^, whereas heterozygous mutations are in italics. Mutations present as heterozygous mutations in all clones of a specific time point and present at a frequency of 50% in the population, are depicted as a frequency of 100%. Frequencies of haplotypes can be found in S2 Table.
(TIF)

S8 Fig Subnetwork selected by Phenetic for reactor 1. The nodes in the network correspond to the genes and/or their associated gene products. Nodes not belonging to any of the enriched terms are colored grey. Mutated genes are indicated by yellow node borders. The different colors for the edges indicate different interaction types. Orange lines represent metabolic interactions, green lines represent protein-protein interactions, and red lines represent protein-DNA interactions. Nodes are colored according to gene function, for each gene the most enriched term is visualized. Genes associated with cell cycle are purple, stress response red, NAD metabolism yellow, heme biosynthesis green and translation initiation cyan.
(TIF)

S9 Fig Subnetwork selected by Phenetic for reactor 2. The nodes in the network correspond to the genes and/or their associated gene products. Nodes not belonging to any of the enriched terms are colored grey. Mutated genes are indicated by yellow node borders. The different colors for the edges indicate different interaction types. Orange lines represent metabolic interactions, green lines represent protein-protein interactions, and red lines represent protein-DNA interactions. Nodes are colored according to gene function, for each gene the most enriched term is visualized. Genes associated with DNA replication are purple, alcohol metabolism red and exocytosis blue.
(TIF)

S10 Fig Subnetwork selected by Phenetic for reactor 3. The nodes in the network correspond to the genes and/or their associated gene products. Nodes not belonging to any of the enriched terms are colored grey. Mutated genes are indicated by yellow node borders. The different colors for the edges indicate different interaction types. Orange lines represent metabolic interactions, green lines represent protein-protein interactions, and red lines represent protein-DNA interactions. Nodes are colored according to gene function, for each gene the most enriched term is visualized. Genes associated with endocytosis are orange, fatty acid transport red, and mitochondrion organization green.
(TIF)

S11 Fig Subnetwork selected by Phenetic for reactor 4. The nodes in the network correspond to the genes and/or their associated gene products. Nodes not belonging to any of the enriched terms are colored grey. Mutated genes are indicated by yellow node borders. The different colors for the edges indicate different interaction types. Orange lines represent metabolic interactions, green lines represent protein-protein interactions, and red lines represent protein-DNA interactions. Nodes are colored according to gene function, for each gene the most enriched term is visualized. Genes associated with alcohol metabolism are red, cell cycle purple, heme biosynthesis green, intracellular signaling cyan, phospholipid catabolism orange and protein catabolism yellow.
(TIF)

S12 Fig Subnetwork selected by Phenetic for reactor 5. The nodes in the network correspond to the genes and/or their associated gene products. Nodes not belonging to any of the enriched terms are colored grey. Mutated genes are indicated by yellow node borders. The different colors for the edges indicate different interaction types. Orange lines represent metabolic interactions, green lines represent protein-protein interactions, and red lines represent protein-DNA interactions. Nodes are colored according to gene function, for each gene the most enriched term is visualized. Genes associated with alcohol biosynthesis are red, cell cycle purple, heme biosynthesis green, isoprenoid metabolism cyan and response to stress orange.
(TIF)

S13 Fig Subnetwork selected by Phenetic for reactor 6. The nodes in the network correspond to the genes and/or their associated gene products. Nodes not belonging to any of the enriched terms are colored grey. Mutated genes are indicated by yellow node borders. The different colors for the edges indicate different interaction types. Orange lines represent metabolic interactions, green lines represent protein-protein interactions, and red lines represent protein-DNA interactions. Nodes are colored according to gene function, for each gene the most enriched term is visualized. Genes associated with cell cycle are purple, chromatin modification green, membrane organization orange and signal transduction cyan.
(TIF)

S14 Fig Competition-based high-resolution fitness profiling of site-directed mutants. Plots show the relative fluorescence of different mutants at different concentrations of ethanol in the growth medium as a function of time (generations). Raw fluorescence measurements correspond to background-corrected YECitrine signal relative to mCherry signal, ln(*YC*/*mCh*). Dye-swap experiments were carried out by competing the YECitrine-tagged mutants with the mCherry-tagged parental strain (orange symbols) or *vice versa* (blue symbols) in three experimental replicates each. The linear least-squares fit of each experiment is shown (Matlab robustfit function). Mutant strains are, from top to bottom: *pca1* ^*C1583T*^, *prt1* ^*A1384G*^, *ybl059w* ^*G479T*^, *intergenic ChrIV A1489310T*, *hem13* ^*G700C*^, *intergenic ChrXII C747403T*, *hst4* ^*G262C*^, *vps70* ^*C595A*^, and *mex67* ^*G456A*^.
(TIF)

S15 Fig Competition assays provide high-resolution replicable fitness data. Scatter plots show the pair-wise correlation of raw slopes (raw *s*) of three experimental replicates (rep1, rep2, rep3) at different ethanol concentrations (0, 4, 6 and 8 (v/v) %).
(TIF)

S16 Fig Evolved clones are less fit in environment without ethanol. Evolved clones of reactor 2 isolated after 200 generations show increased fitness in EtOH, but decreased fitness in medium without ethanol. Data represent means of three biological replicates, error bars represent standard deviations. Fitness is expressed relative to the fitness of the haploid ancestral strain, measured under the same conditions.
(TIF)

S1 Text Supplementary text contains Supplementary Methods. (DOC)

S1 File List of SNPs and Indels identified in evolved clones by whole-genome sequencing (XLS)

S2 File Type and number of mutations found in evolved clones at different time points (PDF)

S3 File CNV observed in all evolved clones. Copy number variations identified in evolved clones from different reactors, across the length of all yeast chromosomes (drawn to scale). Blue and red bars indicate genomic segments where we detected significant evidence of copy number gain or loss, respectively. Timepoints of experiment and isolate numbers are indicated on the left. Upper part of the plot indicates the frequency of specific CNVs in the indicated chromosomal regions, expressed as the percentage of isolated clones that harboured them. Bottom part shows the CNV patterns in all individual clones.
(PDF)

S4 File List of SNPs and Indels identified in evolved populations by whole-genome sequencing. (XLS)

S5 File Term-enrichment analysis of mutated genes. (XLS)

S6 File Phenetic data. (XLS)

S7 File List of functional domains mutated in evolved lineages. (XLS)

S1 Table Genes hit multiple times across reactors and populations. This table lists all genes hit multiple times in different reactors and populations (genes hit at different positions; mutations identical at the nucleotide level are excluded from this analyses).
(DOC)

S2 Table Haplotype frequencies of reactor 1 and 2. (DOCX)

S3 Table Interactome analyses. Specific pathways hit in the different evolved populations, with FDR values. Mutated genes are the number of genes of this specific pathway mutated in the specific reactor, genes present in genome is the total number of genes in the genome for a particular pathway. As reactors 2 and 6 are mutators for Indels and SNPs respectively, we analyzed enrichments separately for SNPs and Indels to be able to compare between populations.
(DOCX)

S4 Table List of strains used in this study. (DOC)

S5 Table List of primers used in this study. (DOCX)

S6 Table Fitness data of strains used in Fig 8. (DOC)

S7 Table Statistical analysis of fitness data of site-directed mutant strains. (DOC)
