# Supplementary figures and images for: Adaptation to High Ethanol Reveals Complex Evolutionary Pathways
Source: PLoS Genet. 2015 Nov 6;11(11):e1005635. doi: 10.1371/journal.pgen.1005635 (PMC4636377; doi:10.1371/journal.pgen.1005635)

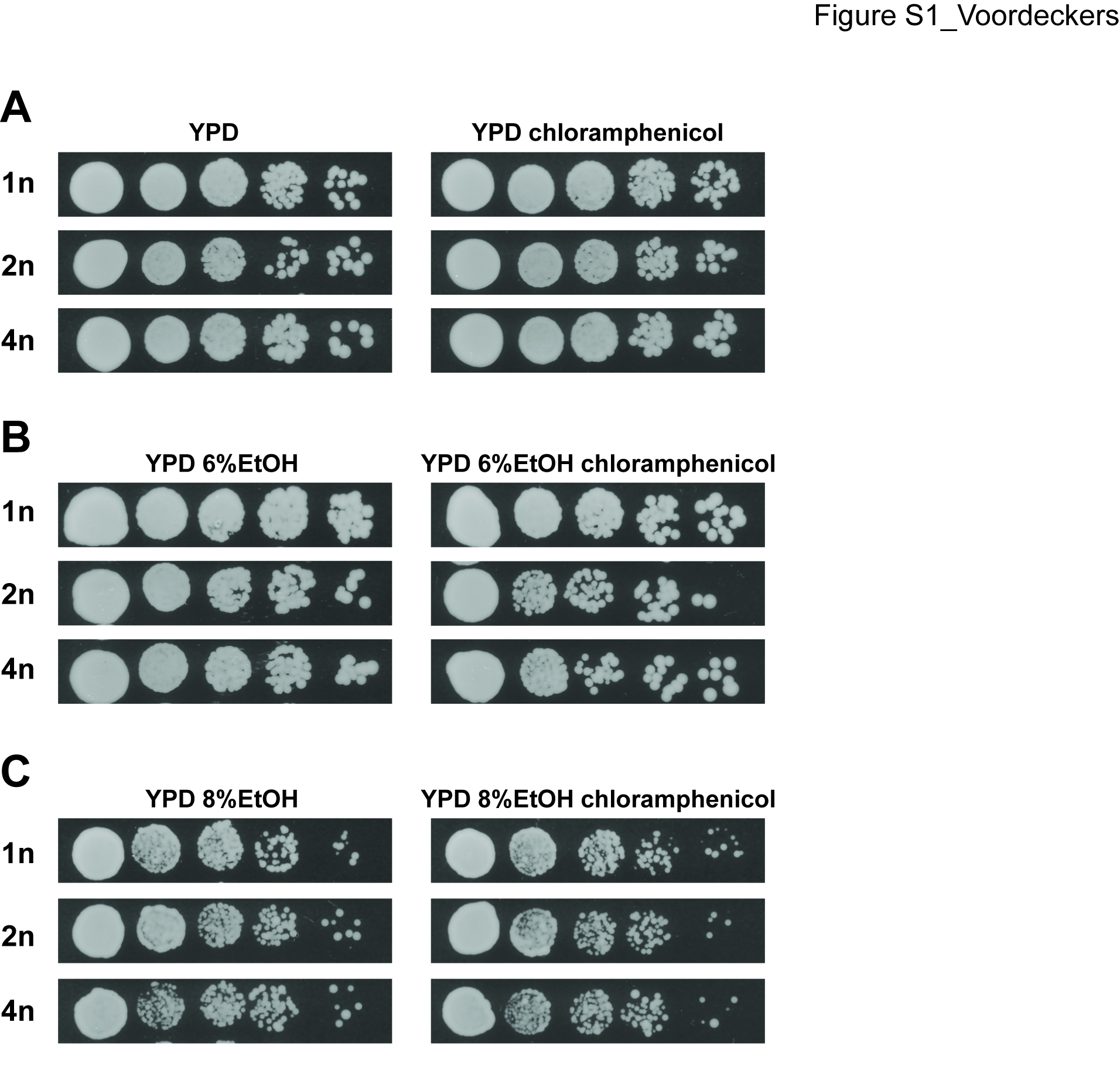

Supplement: S1 Fig — Tenfold serial dilutions of ancestral haploid, diploid and tetraploid strains (starting OD600nm = 1.0) were spotted on agar plates. (A) growth in YPD and YPD containing 50 μg/ml chloramphenicol after 48 hours. (B) growth in YPD 6% ethanol and YPD 6% ethanol containing 50 μg/ml chloramphenicol after 96 hours. (C) growth in YPD 8% ethanol and YPD 8% ethanol containing 50 μg/ml chloramphenicol after 96 hours. (TIF) [file pgen.1005635.s001.tif]

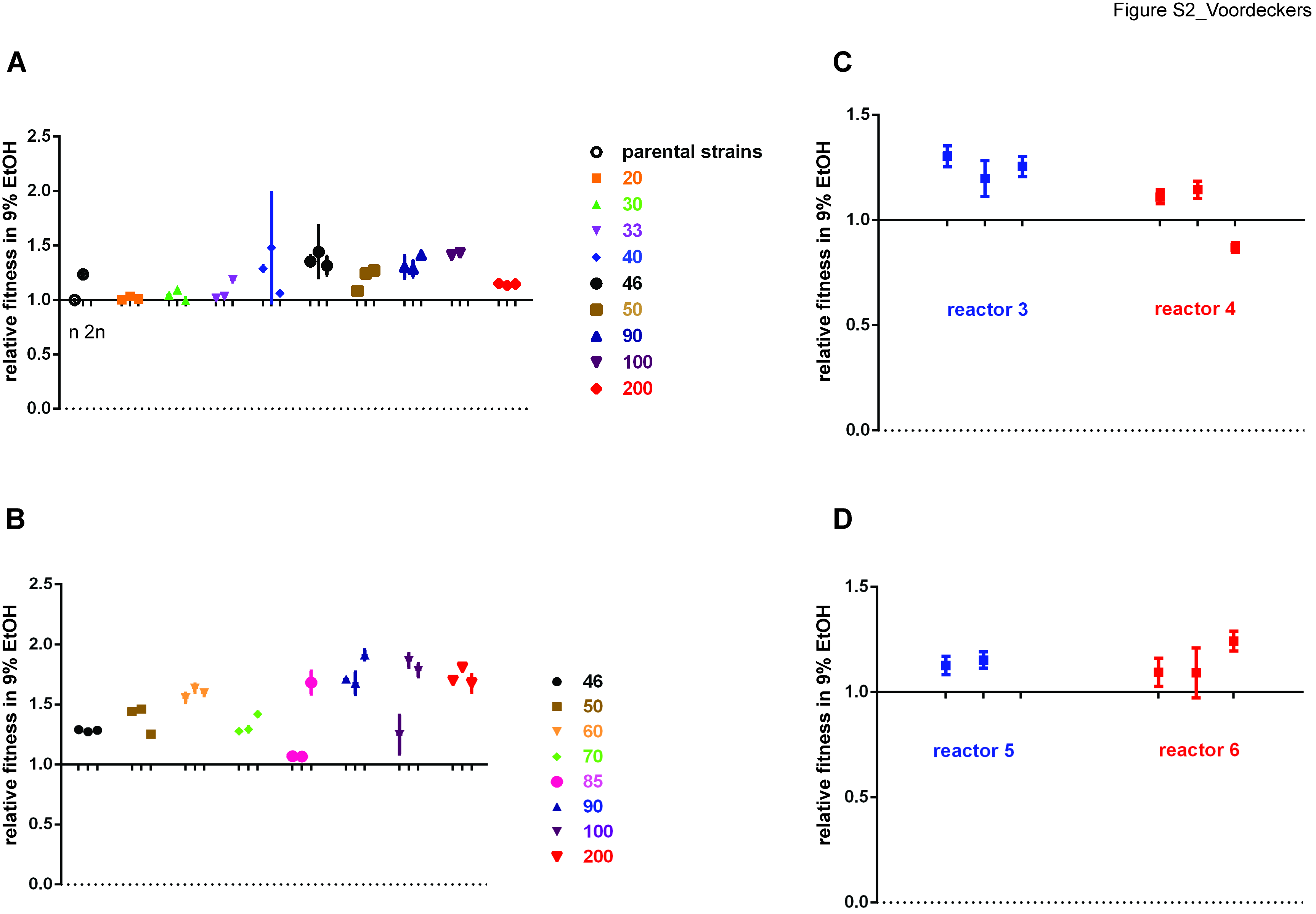

Supplement: S2 Fig — Evolved clones from different reactors show increased fitness in EtOH. Data represent means of three biological replicates, error bars represent standard deviations. Clones from the same time point (number of generations) are depicted in the same color. (A) Fitness of sequenced, evolved clones of reactor 1, determined in 9% EtOH. Fitness is expressed relative to the fitness of the haploid ancestral strain. Fitness of the ancestral, isogenic haploid and diploid strains is also depicted in this figure. A diploid strain is more fit than an isogenic haploid strain in 9% EtOH. (B) Fitness of sequenced, evolved clones of reactor 2, determined in 9% EtOH. Fitness is expressed relative to the fitness of the haploid ancestral strain. (C) Fitness of sequenced, evolved clones of reactor 3 (blue) and reactor 4 (red) after 200 generations, determined in 9% EtOH. Fitness is expressed relative to the fitness of the diploid ancestral strain. (D) Fitness of sequenced, evolved clones of reactor 5 (blue) and reactor 6 (red) after 200 generations, determined in 9% EtOH. Fitness is expressed relative to the fitness of the tetraploid ancestral strain. Fitness of one of the evolved clones of reactor 5 could not be determined. (TIF) [file pgen.1005635.s002.tif]

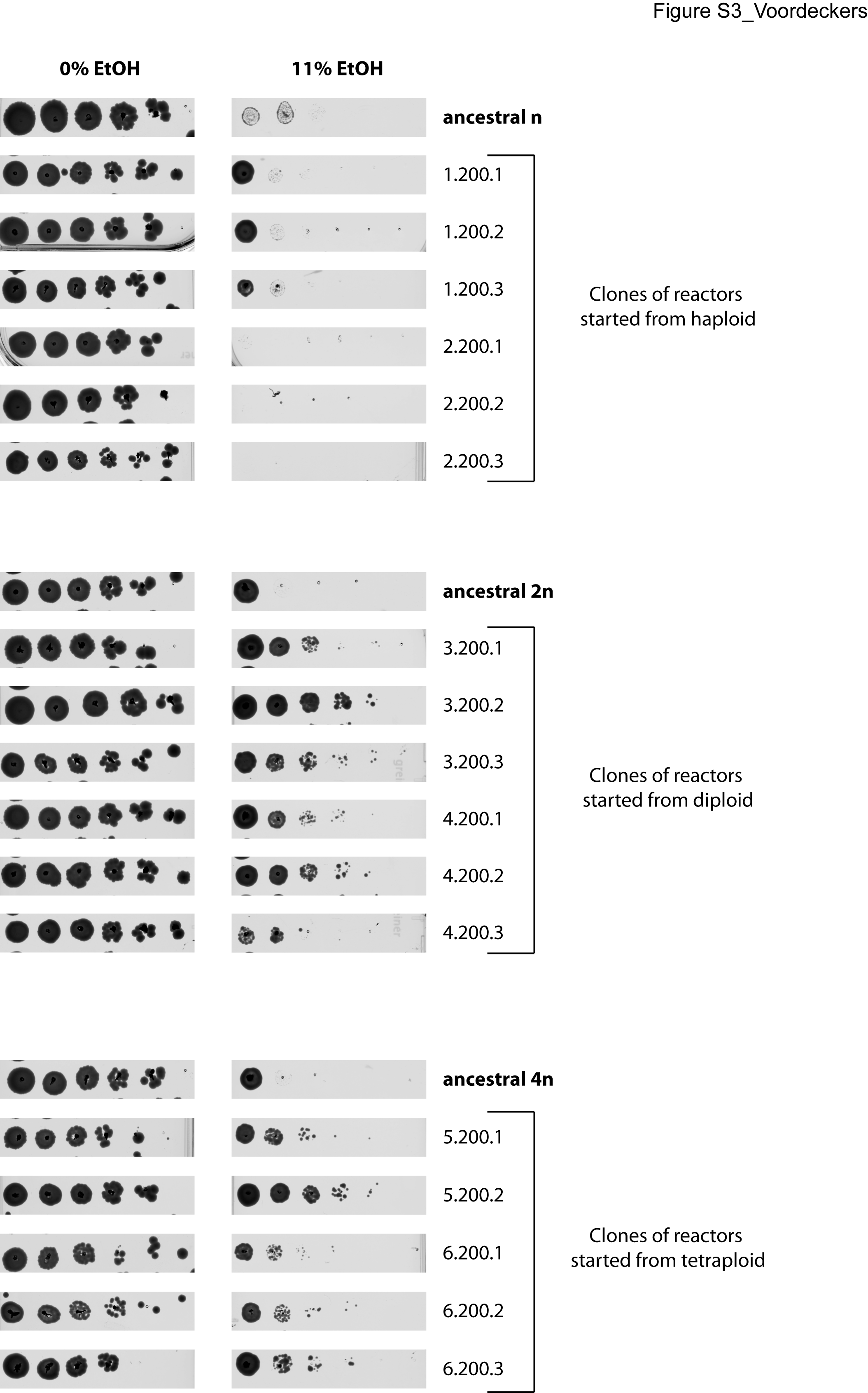

Supplement: S3 Fig — Tenfold serial dilutions of evolved clones (starting OD600nm = 1.0) isolated after 200 generations from the different reactors were spotted on YPD (2%) agar plates containing 0 (left panel) or 11% (right panel) EtOH (v/v) to assess their ethanol tolerance. Plates were carefully sealed with parafilm to prevent ethanol evaporation and incubated for 3 or 10 days at 30°C (for 0 and 11% EtOH respectively). Different strains were randomized for spotting on plates, the different panels shown are taken from the same plate. Evolved clones from reactor 2 do not grow in 11% EtOH on agar plates, although they show high fitness in 9% ethanol in liquid medium (S2 Fig). This could reflect differences in the mutations required to tolerate 9% vs 11% ethanol, and/or differences between growth on liquid and solid medium. (TIF) [file pgen.1005635.s003.tif]

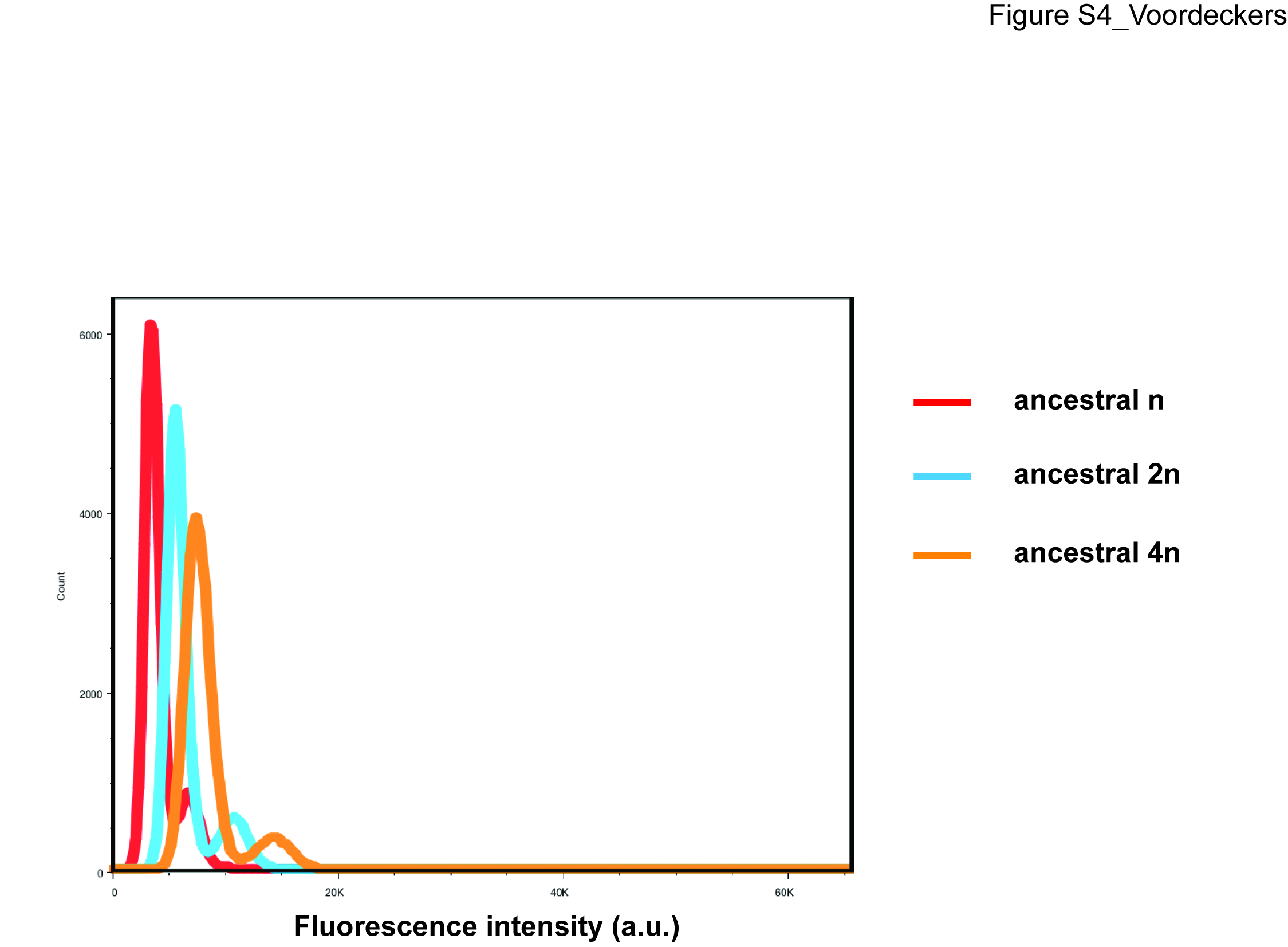

Supplement: S4 Fig — Flow cytometry analysis of DNA content (stained by propidium iodide) of ancestral haploid (red), diploid (blue) and tetraploid (orange) strains. (TIF) [file pgen.1005635.s004.tif]

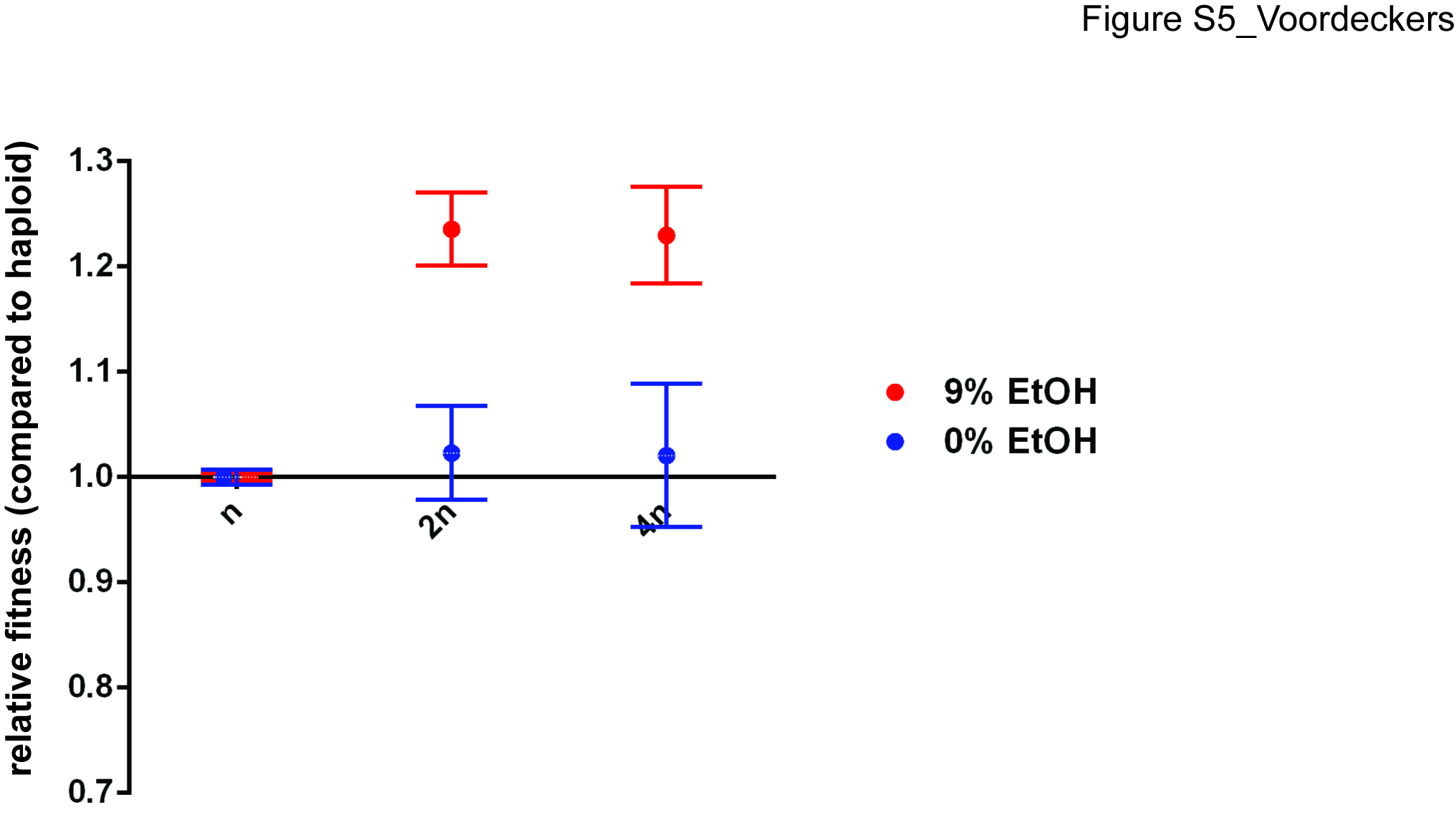

Supplement: S5 Fig — Fitness of isogenic haploid, diploid and tetraploid strains was determined in 0 and 9% EtOH. Fitness is expressed relative to the fitness of the haploid strain under a specific condition. A diploid strain is significantly more fit than a haploid strain in 9% EtOH. (TIF) [file pgen.1005635.s005.tif]

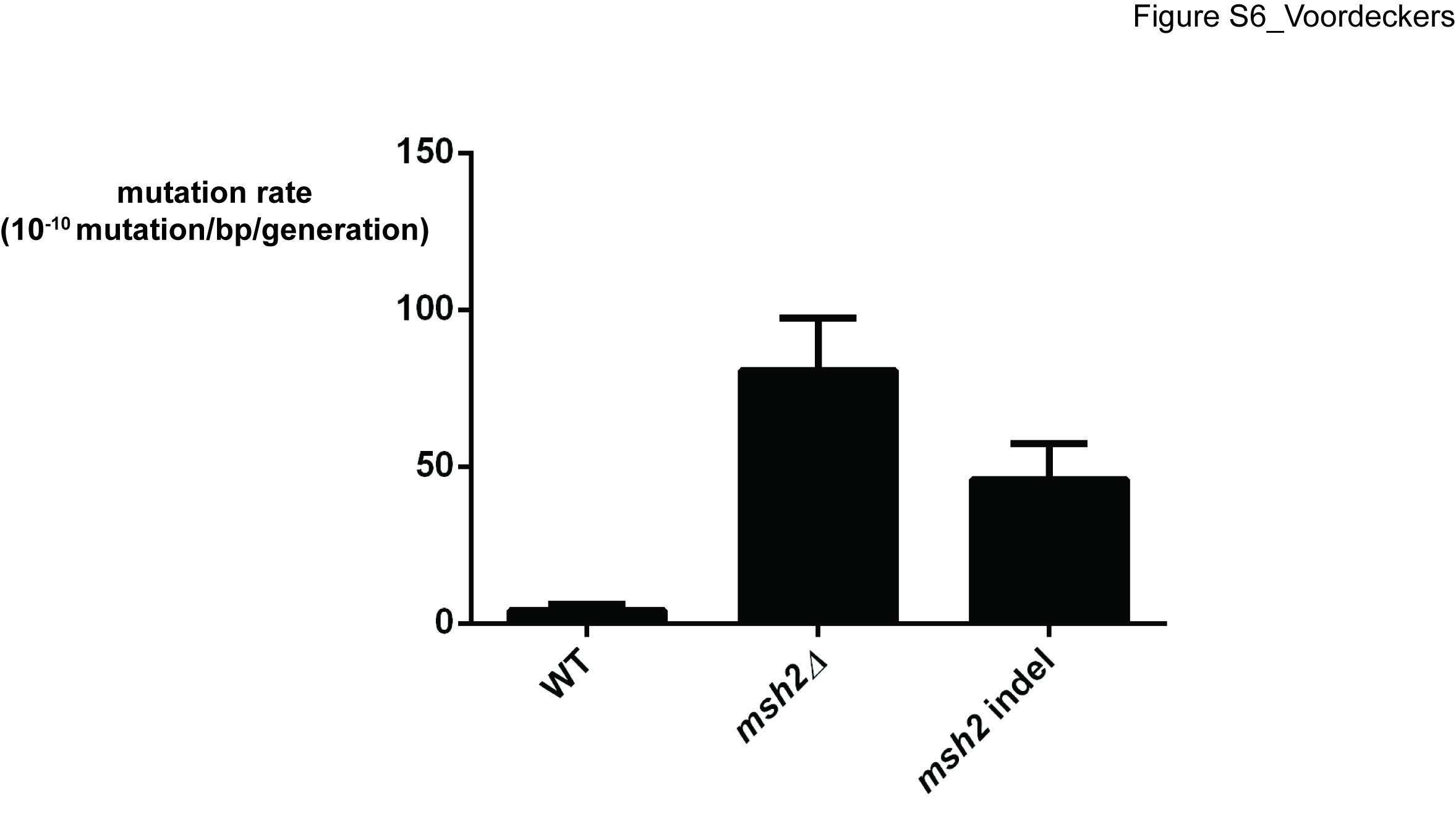

Supplement: S6 Fig — Msh2 C1630 CACGTAATGACGCCAAGGAGTT (labeled msh2 indel in figure) and msh2Δ strains show elevated mutation rate compared to wild type strain. Fluctuation assays were used to determine mutation rates; error bars represent 95% confidence intervals. (TIF) [file pgen.1005635.s006.tif]

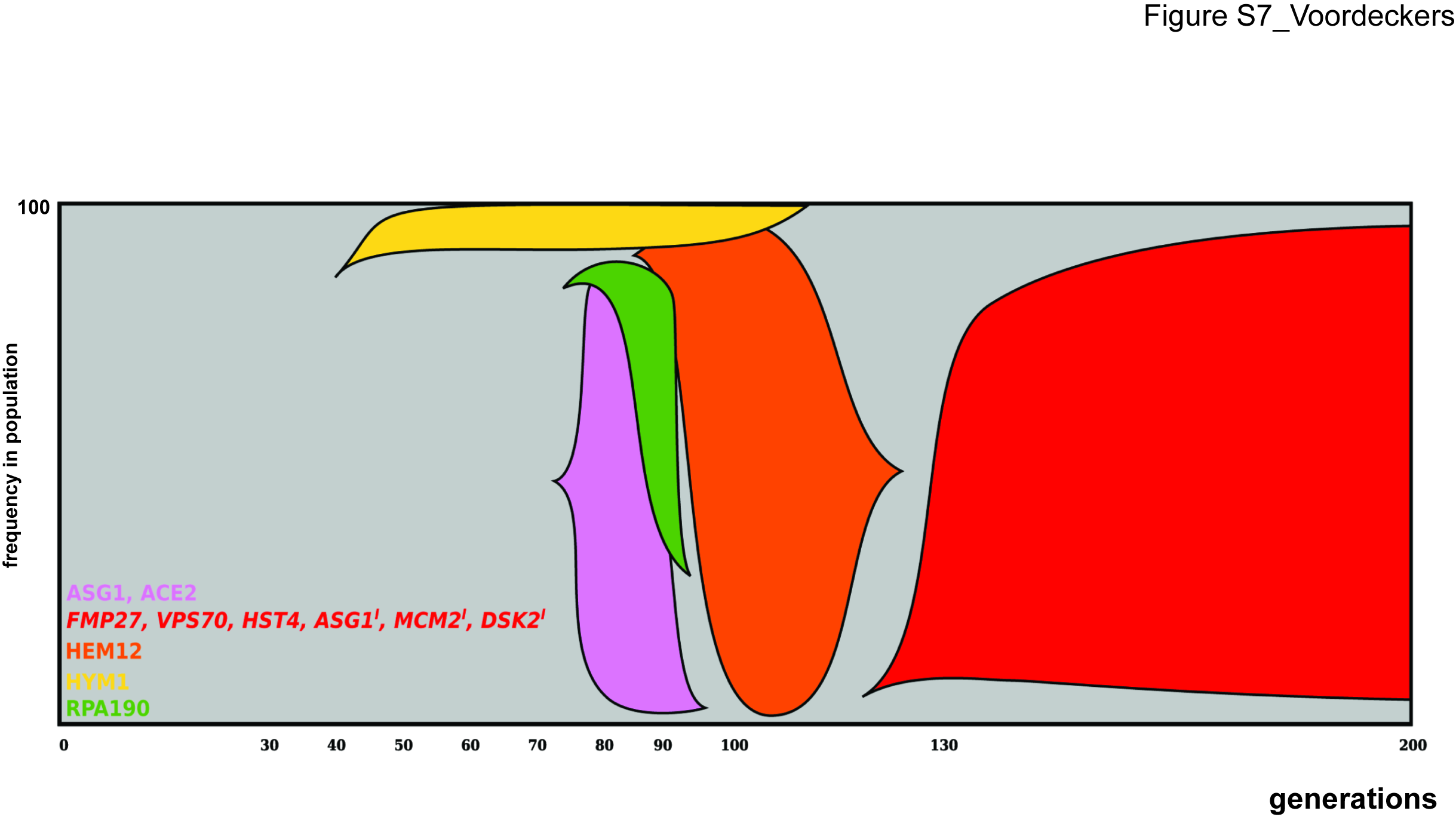

Supplement: S7 Fig — Mutations (reaching a frequency of at least 20%) and corresponding frequencies were identified from population sequencing data. Muller diagram represents the hierarchical clustering of these mutations, with each color block representing a specific group of linked mutations. Indels are designated with I, whereas heterozygous mutations are in italics. Mutations present as heterozygous mutations in all clones of a specific time point and present at a frequency of 50% in the population, are depicted as a frequency of 100%. Frequencies of haplotypes can be found in S2 Table. (TIF) [file pgen.1005635.s007.tif]

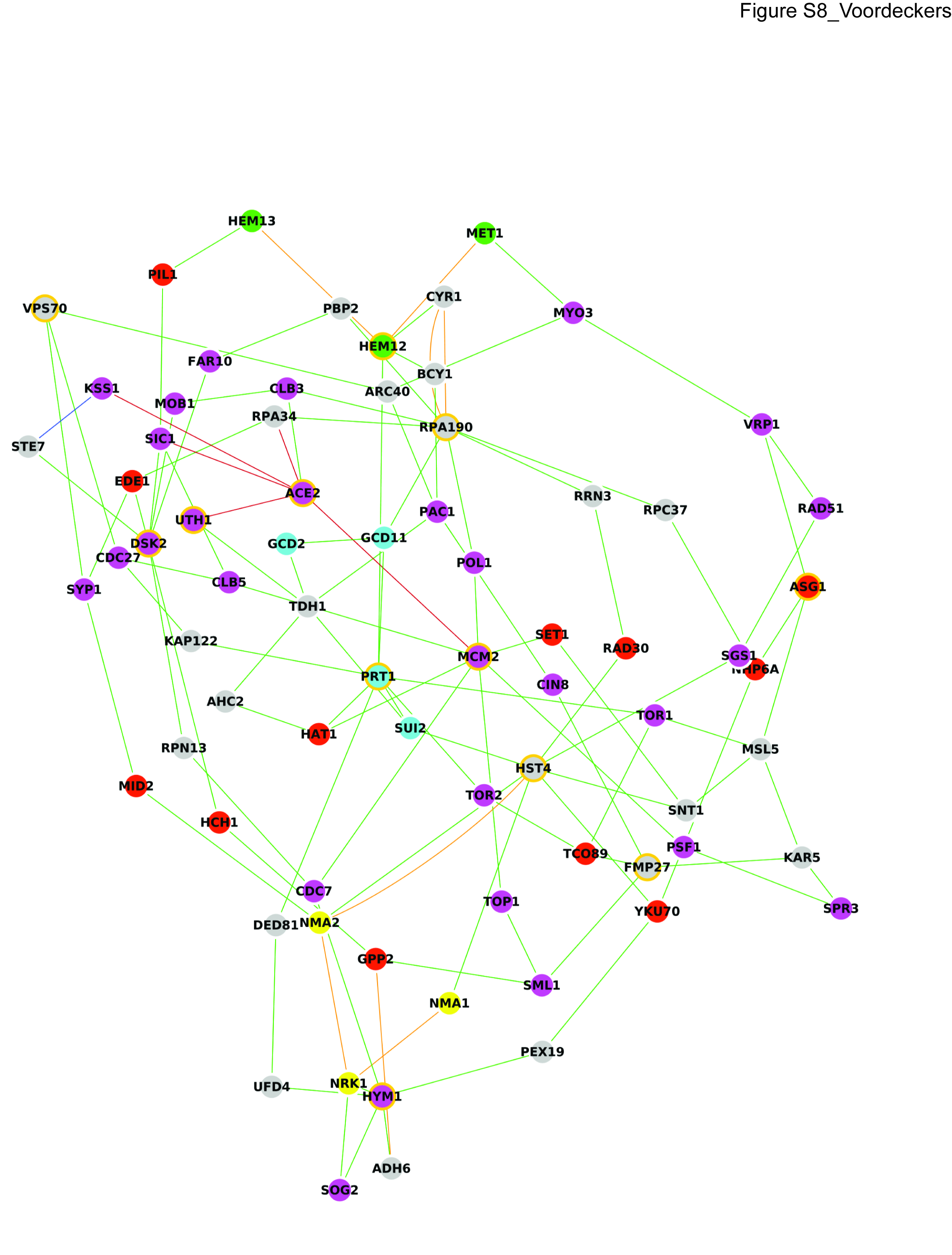

Supplement: S8 Fig — The nodes in the network correspond to the genes and/or their associated gene products. Nodes not belonging to any of the enriched terms are colored grey. Mutated genes are indicated by yellow node borders. The different colors for the edges indicate different interaction types. Orange lines represent metabolic interactions, green lines represent protein-protein interactions, and red lines represent protein-DNA interactions. Nodes are colored according to gene function, for each gene the most enriched term is visualized. Genes associated with cell cycle are purple, stress response red, NAD metabolism yellow, heme biosynthesis green and translation initiation cyan. (TIF) [file pgen.1005635.s008.tif]

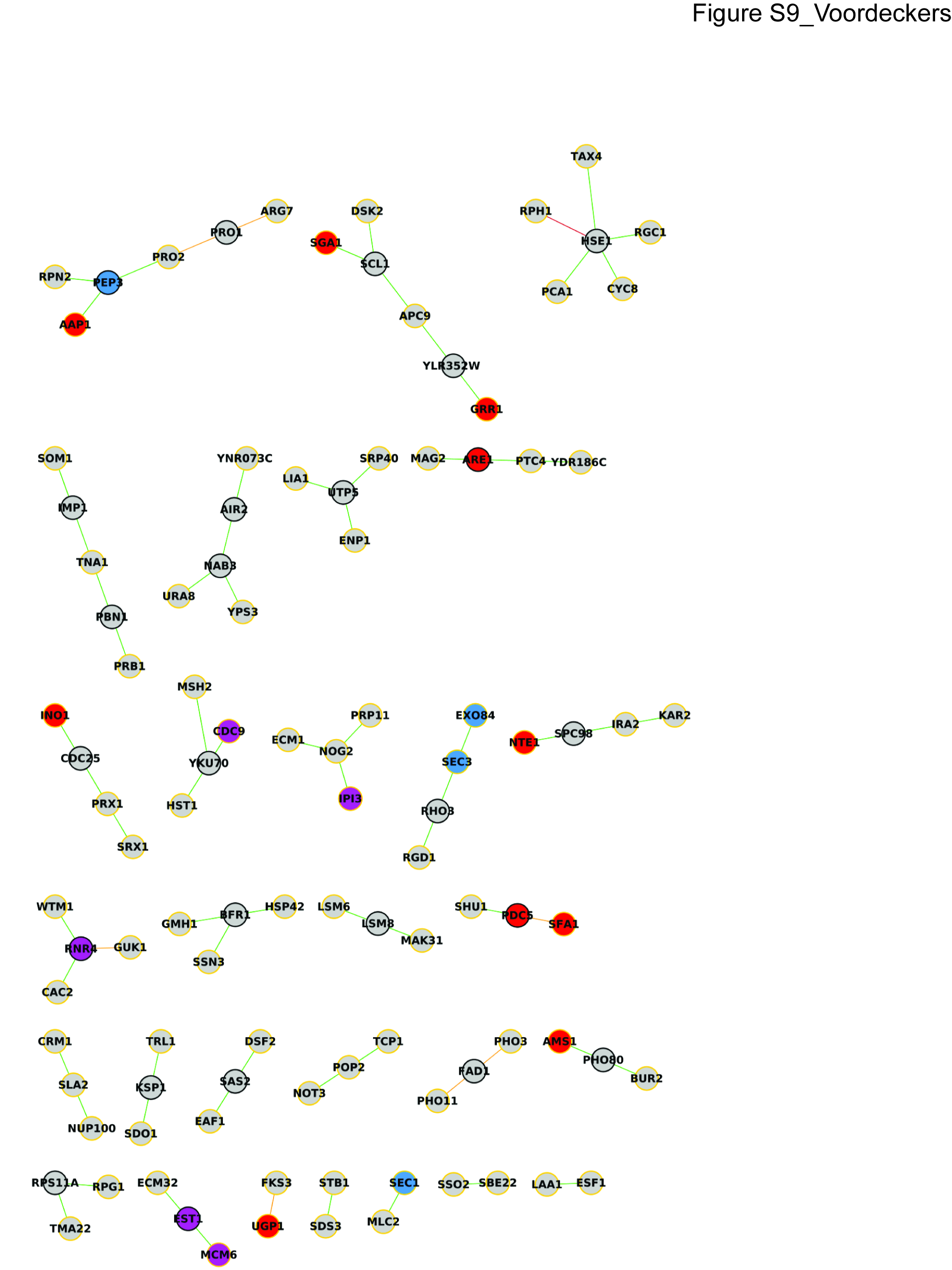

Supplement: S9 Fig — The nodes in the network correspond to the genes and/or their associated gene products. Nodes not belonging to any of the enriched terms are colored grey. Mutated genes are indicated by yellow node borders. The different colors for the edges indicate different interaction types. Orange lines represent metabolic interactions, green lines represent protein-protein interactions, and red lines represent protein-DNA interactions. Nodes are colored according to gene function, for each gene the most enriched term is visualized. Genes associated with DNA replication are purple, alcohol metabolism red and exocytosis blue. (TIF) [file pgen.1005635.s009.tif]

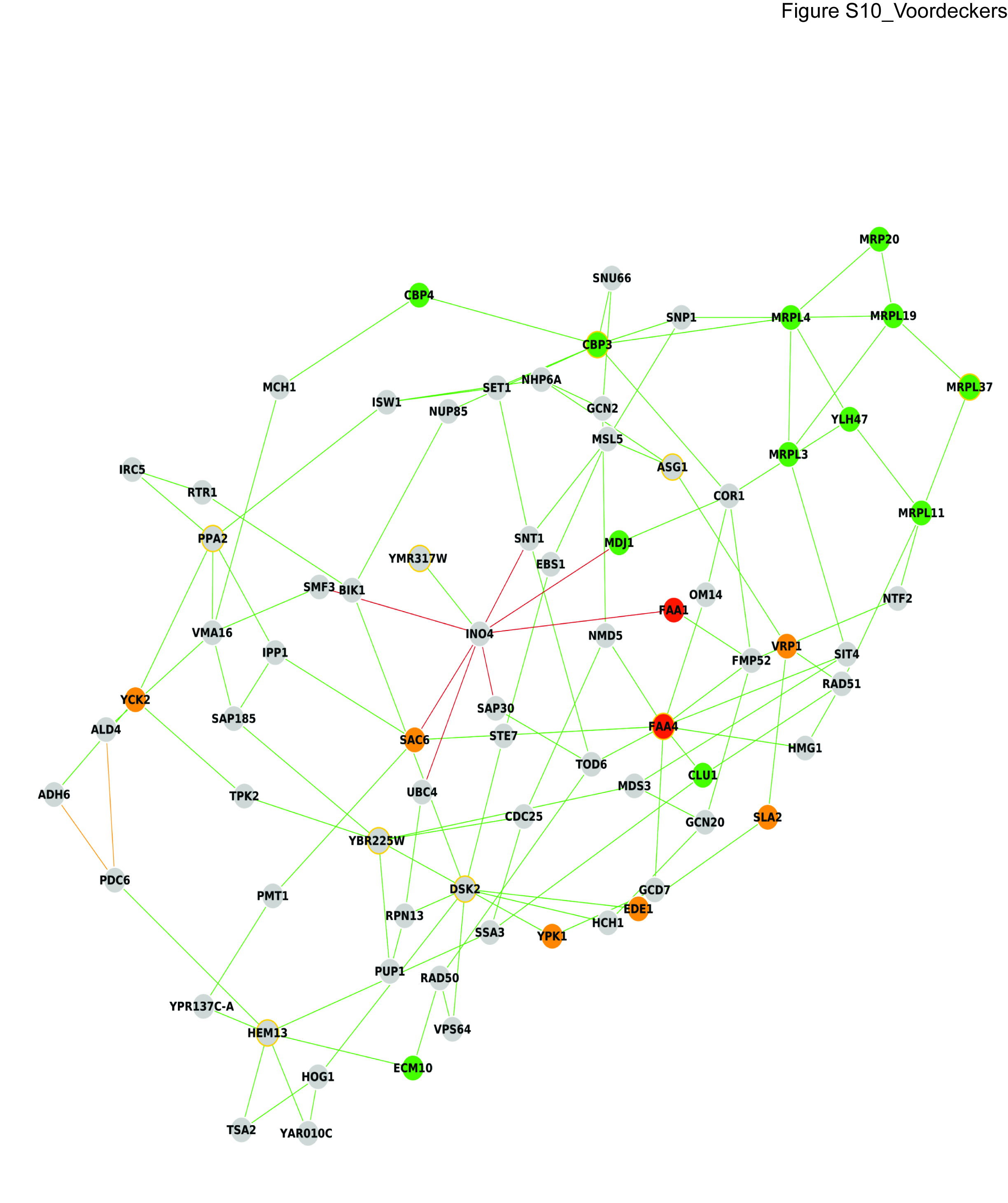

Supplement: S10 Fig — The nodes in the network correspond to the genes and/or their associated gene products. Nodes not belonging to any of the enriched terms are colored grey. Mutated genes are indicated by yellow node borders. The different colors for the edges indicate different interaction types. Orange lines represent metabolic interactions, green lines represent protein-protein interactions, and red lines represent protein-DNA interactions. Nodes are colored according to gene function, for each gene the most enriched term is visualized. Genes associated with endocytosis are orange, fatty acid transport red, and mitochondrion organization green. (TIF) [file pgen.1005635.s010.tif]

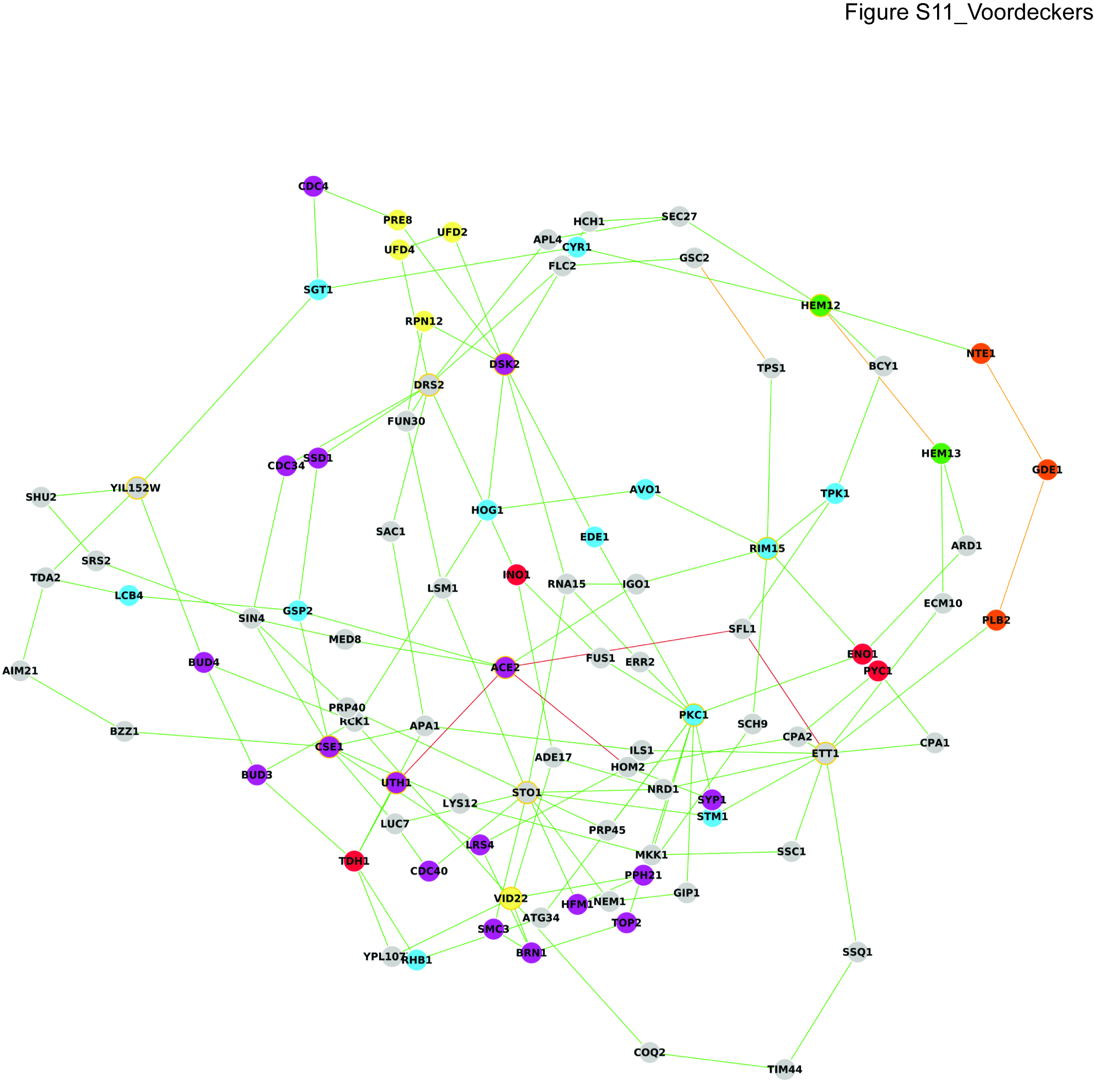

Supplement: S11 Fig — The nodes in the network correspond to the genes and/or their associated gene products. Nodes not belonging to any of the enriched terms are colored grey. Mutated genes are indicated by yellow node borders. The different colors for the edges indicate different interaction types. Orange lines represent metabolic interactions, green lines represent protein-protein interactions, and red lines represent protein-DNA interactions. Nodes are colored according to gene function, for each gene the most enriched term is visualized. Genes associated with alcohol metabolism are red, cell cycle purple, heme biosynthesis green, intracellular signaling cyan, phospholipid catabolism orange and protein catabolism yellow. (TIF) [file pgen.1005635.s011.tif]

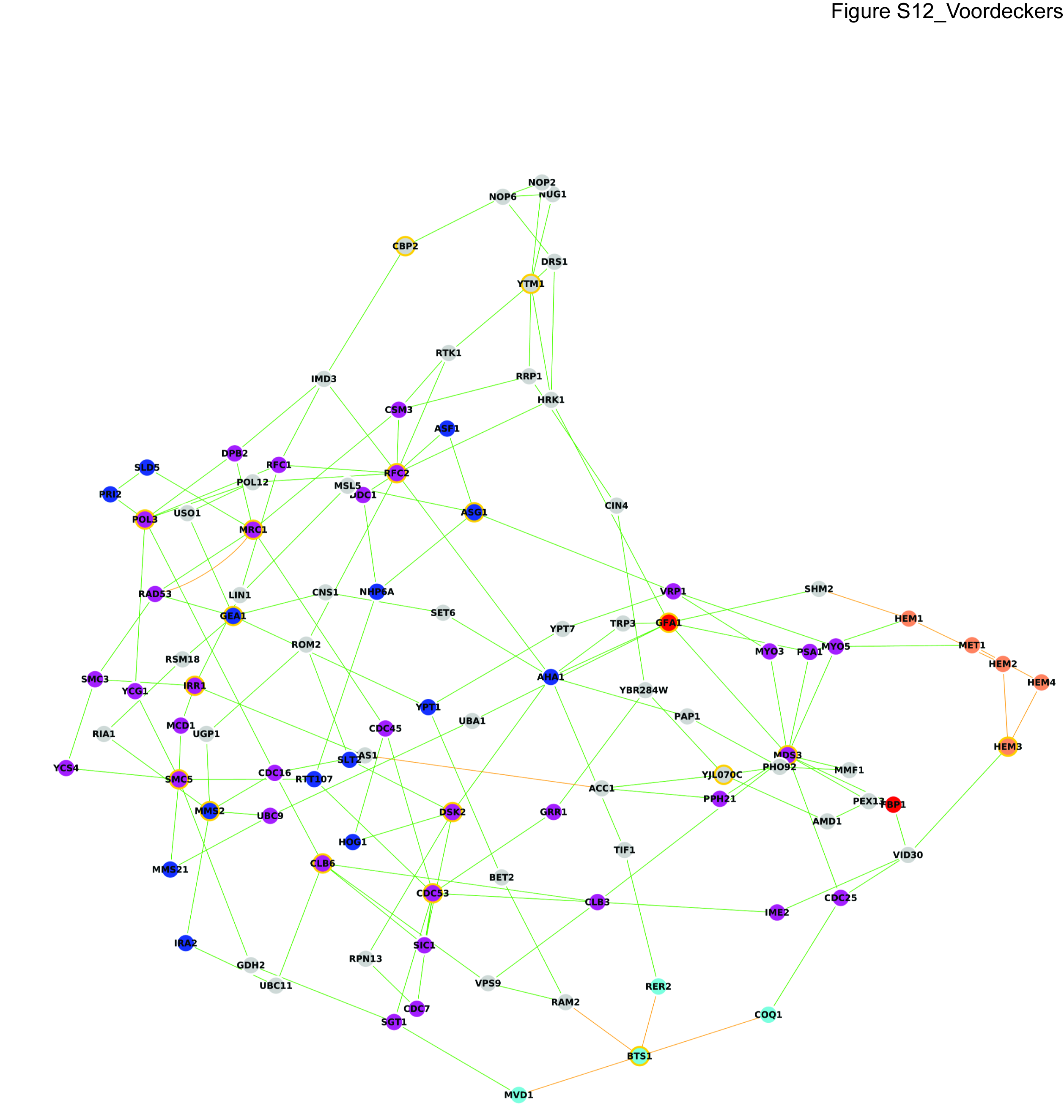

Supplement: S12 Fig — The nodes in the network correspond to the genes and/or their associated gene products. Nodes not belonging to any of the enriched terms are colored grey. Mutated genes are indicated by yellow node borders. The different colors for the edges indicate different interaction types. Orange lines represent metabolic interactions, green lines represent protein-protein interactions, and red lines represent protein-DNA interactions. Nodes are colored according to gene function, for each gene the most enriched term is visualized. Genes associated with alcohol biosynthesis are red, cell cycle purple, heme biosynthesis green, isoprenoid metabolism cyan and response to stress orange. (TIF) [file pgen.1005635.s012.tif]

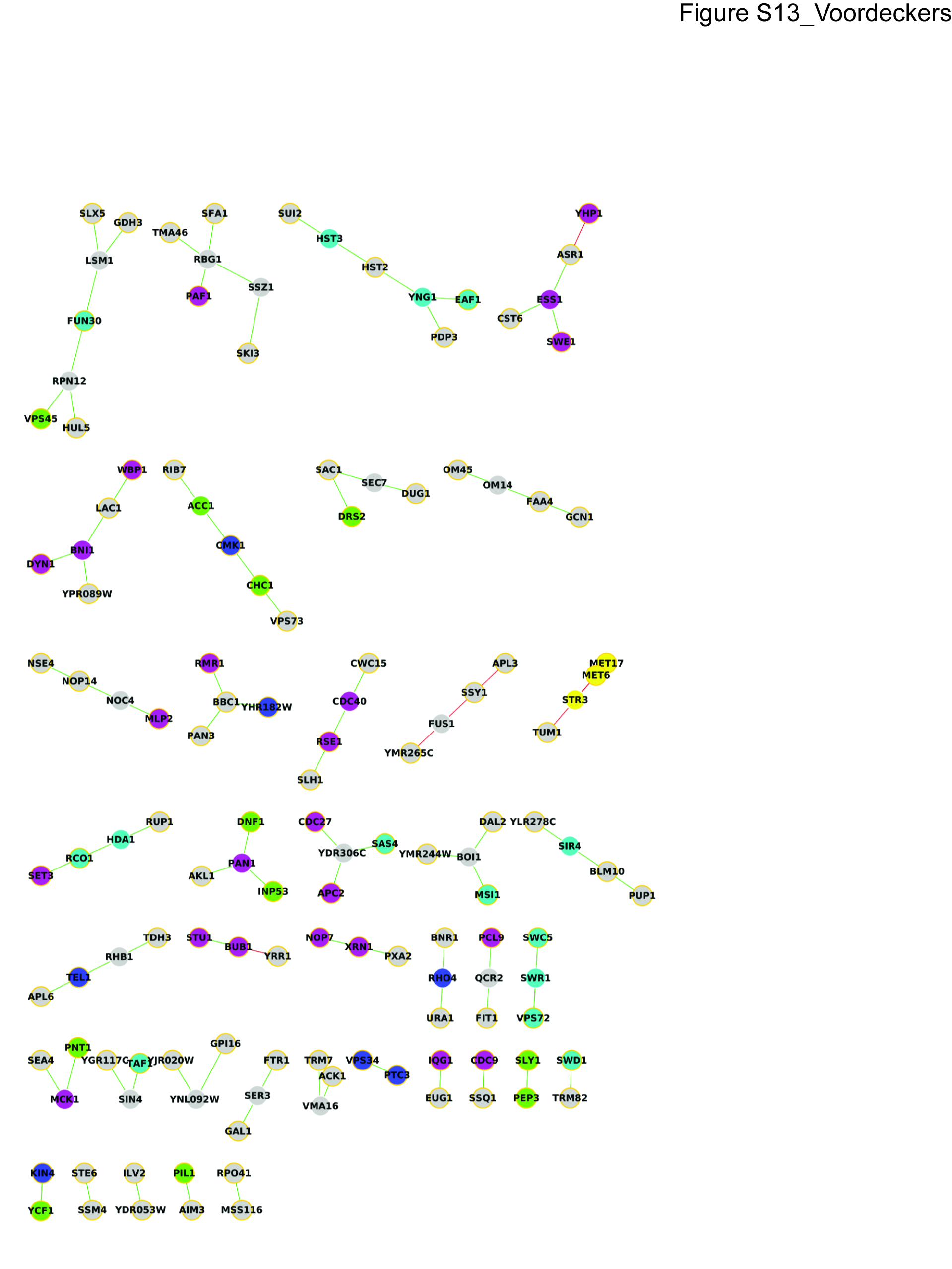

Supplement: S13 Fig — The nodes in the network correspond to the genes and/or their associated gene products. Nodes not belonging to any of the enriched terms are colored grey. Mutated genes are indicated by yellow node borders. The different colors for the edges indicate different interaction types. Orange lines represent metabolic interactions, green lines represent protein-protein interactions, and red lines represent protein-DNA interactions. Nodes are colored according to gene function, for each gene the most enriched term is visualized. Genes associated with cell cycle are purple, chromatin modification green, membrane organization orange and signal transduction cyan. (TIF) [file pgen.1005635.s013.tif]

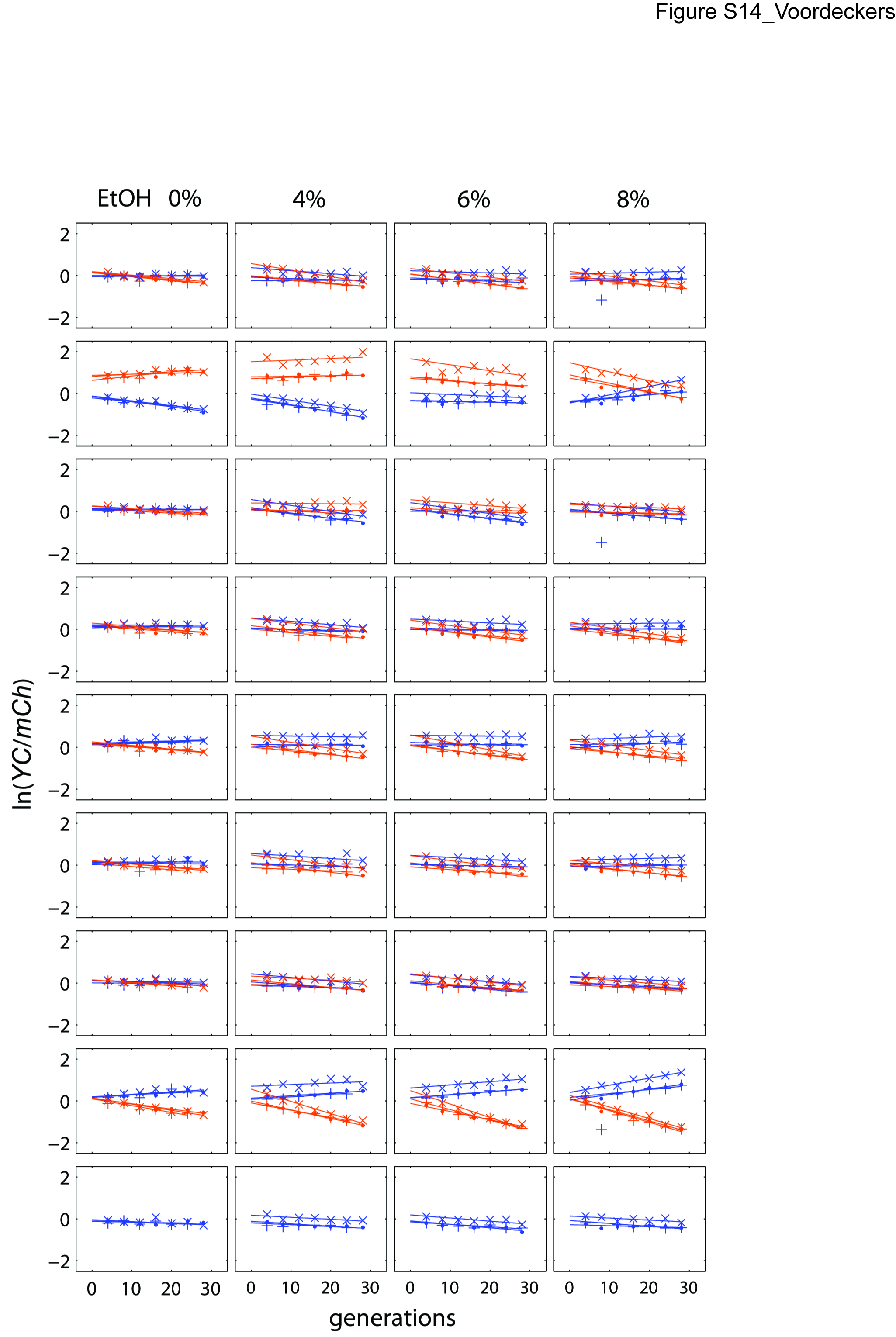

Supplement: S14 Fig — Plots show the relative fluorescence of different mutants at different concentrations of ethanol in the growth medium as a function of time (generations). Raw fluorescence measurements correspond to background-corrected YECitrine signal relative to mCherry signal, ln(YC/mCh). Dye-swap experiments were carried out by competing the YECitrine-tagged mutants with the mCherry-tagged parental strain (orange symbols) or vice versa (blue symbols) in three experimental replicates each. The linear least-squares fit of each experiment is shown (Matlab robustfit function). Mutant strains are, from top to bottom: pca1 C1583T, prt1 A1384G, ybl059w G479T, intergenic ChrIV A1489310T, hem13 G700C, intergenic ChrXII C747403T, hst4 G262C, vps70 C595A, and mex67 G456A. (TIF) [file pgen.1005635.s014.tif]

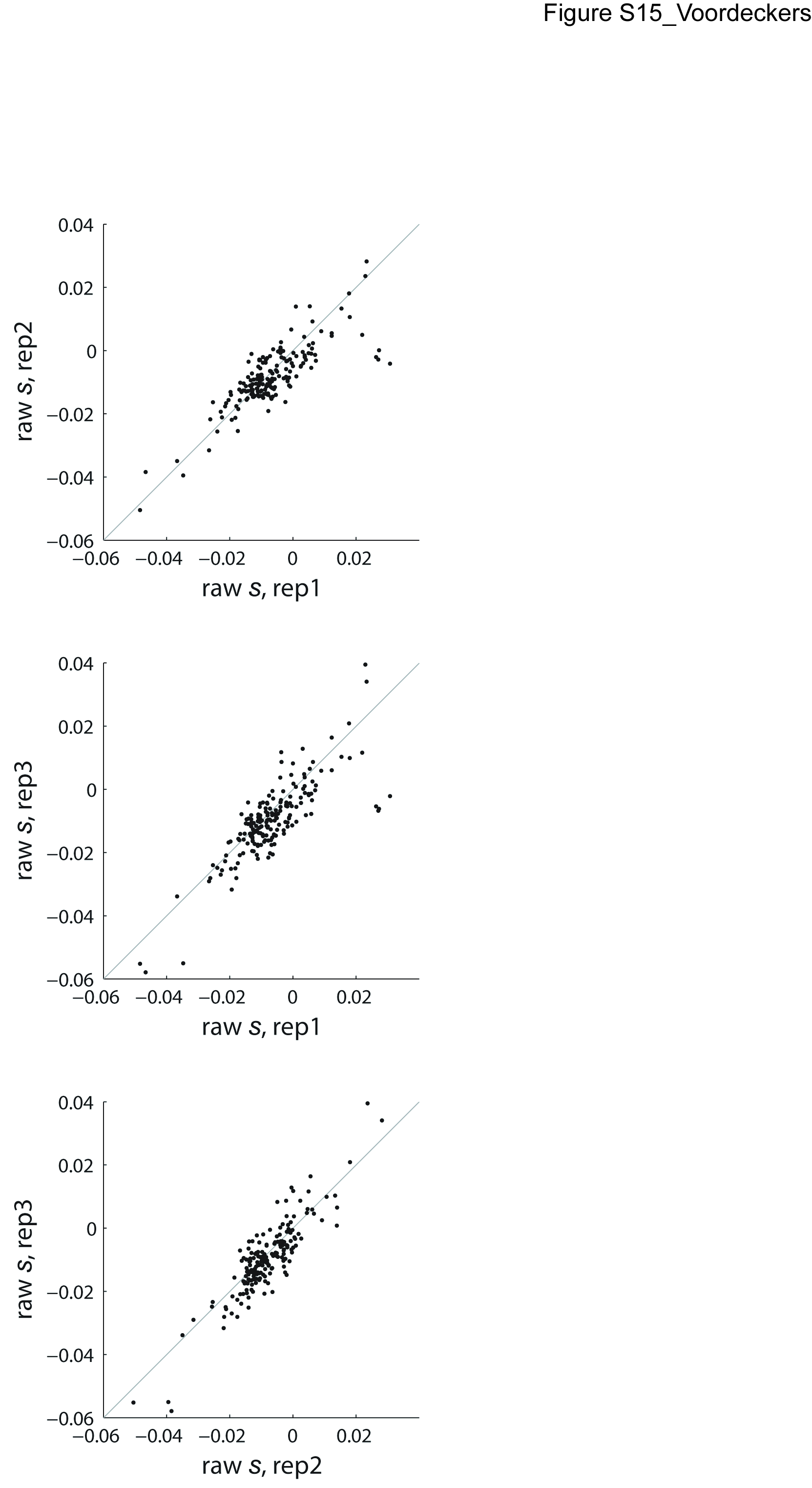

Supplement: S15 Fig — Scatter plots show the pair-wise correlation of raw slopes (raw s) of three experimental replicates (rep1, rep2, rep3) at different ethanol concentrations (0, 4, 6 and 8 (v/v) %). (TIF) [file pgen.1005635.s015.tif]

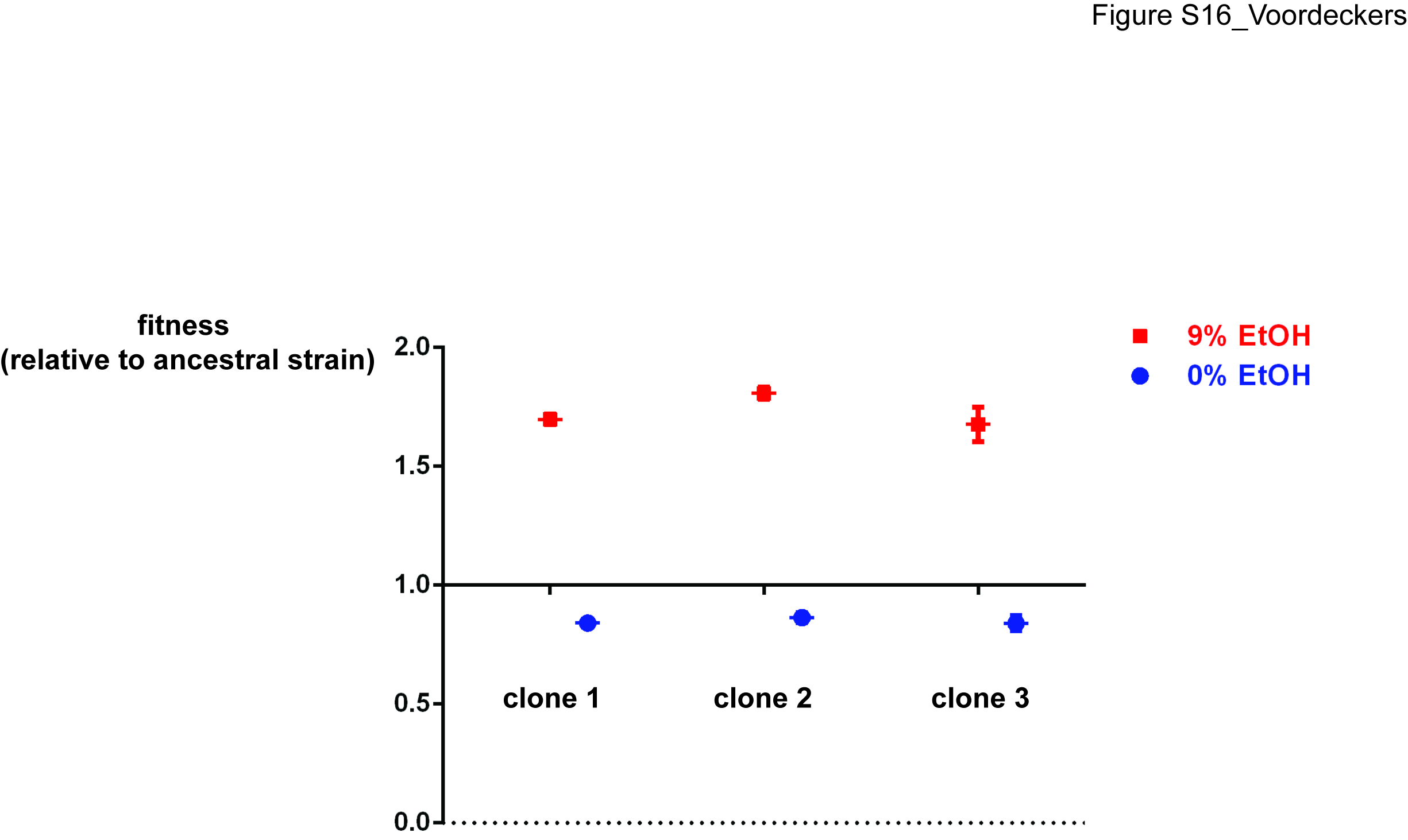

Supplement: S16 Fig — Evolved clones of reactor 2 isolated after 200 generations show increased fitness in EtOH, but decreased fitness in medium without ethanol. Data represent means of three biological replicates, error bars represent standard deviations. Fitness is expressed relative to the fitness of the haploid ancestral strain, measured under the same conditions. (TIF) [file pgen.1005635.s016.tif]

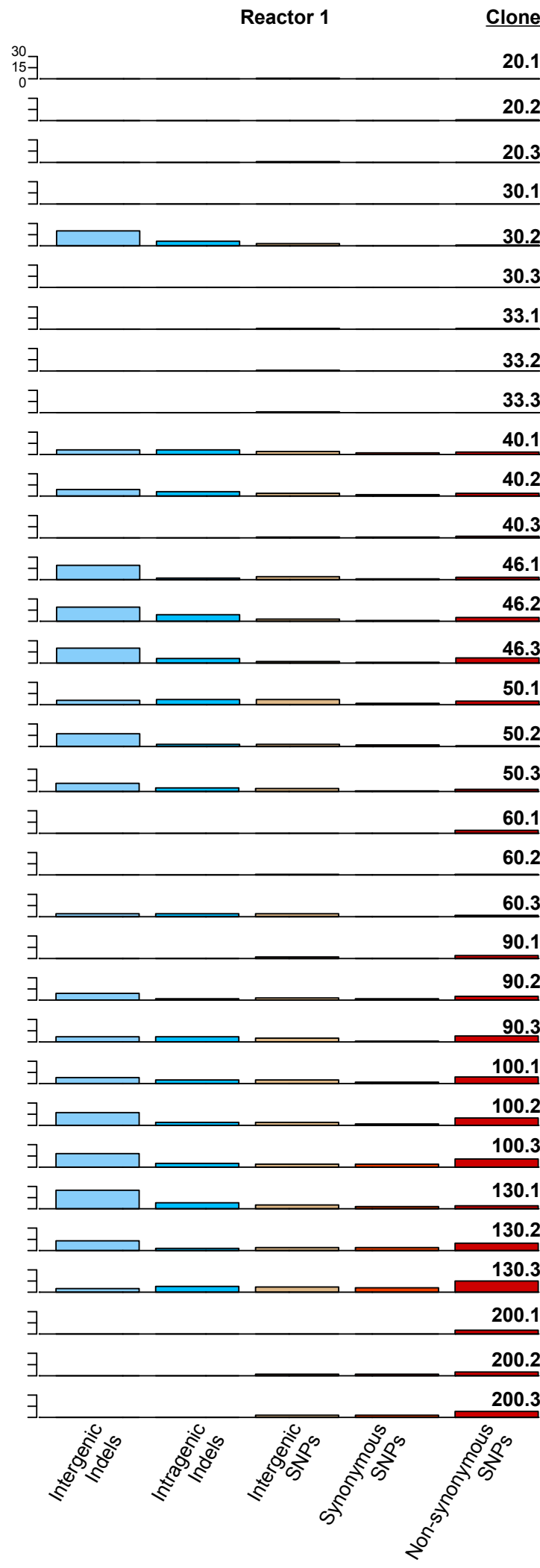

# Reactor 2

# Clones with mutator phenotype

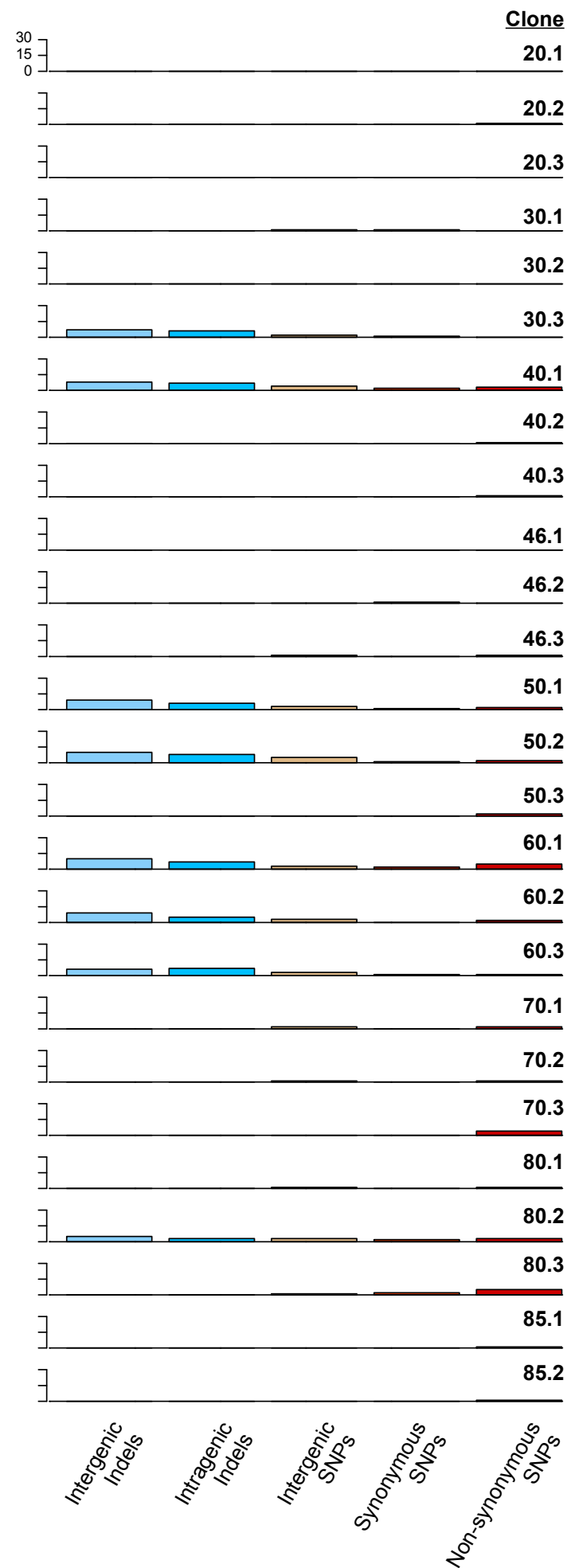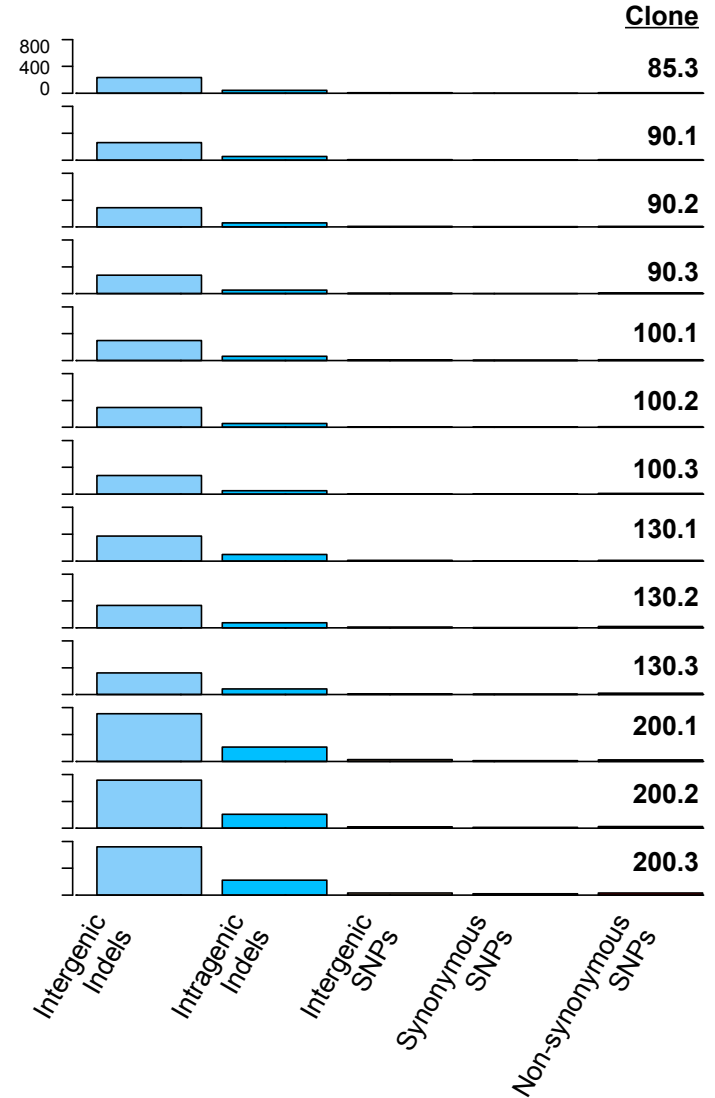

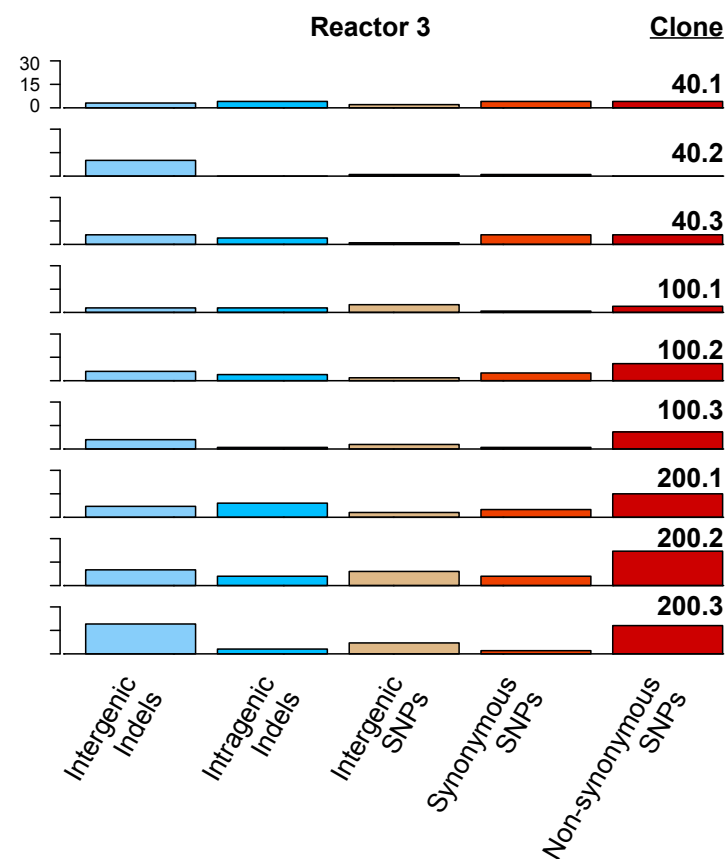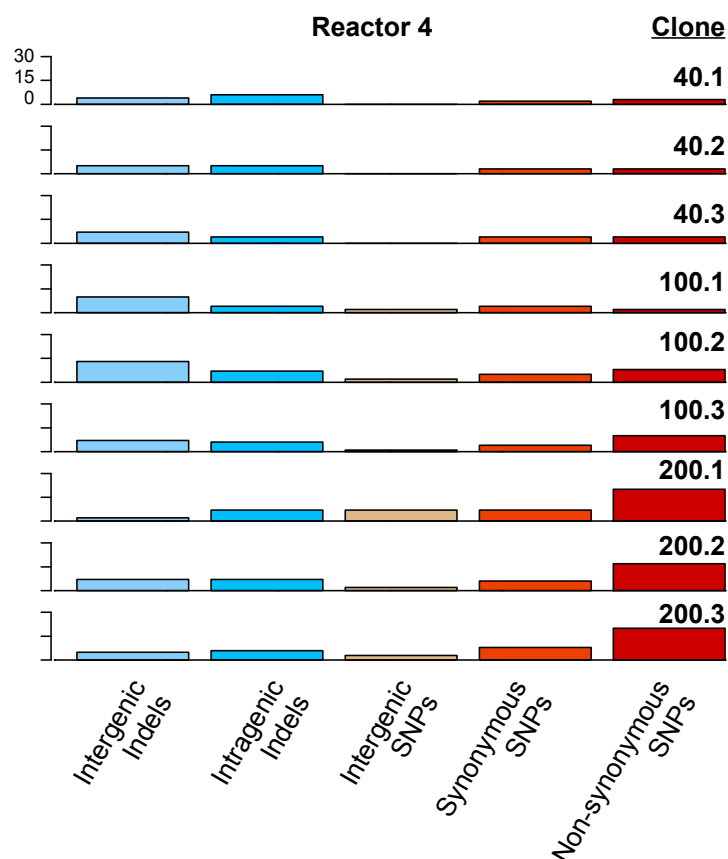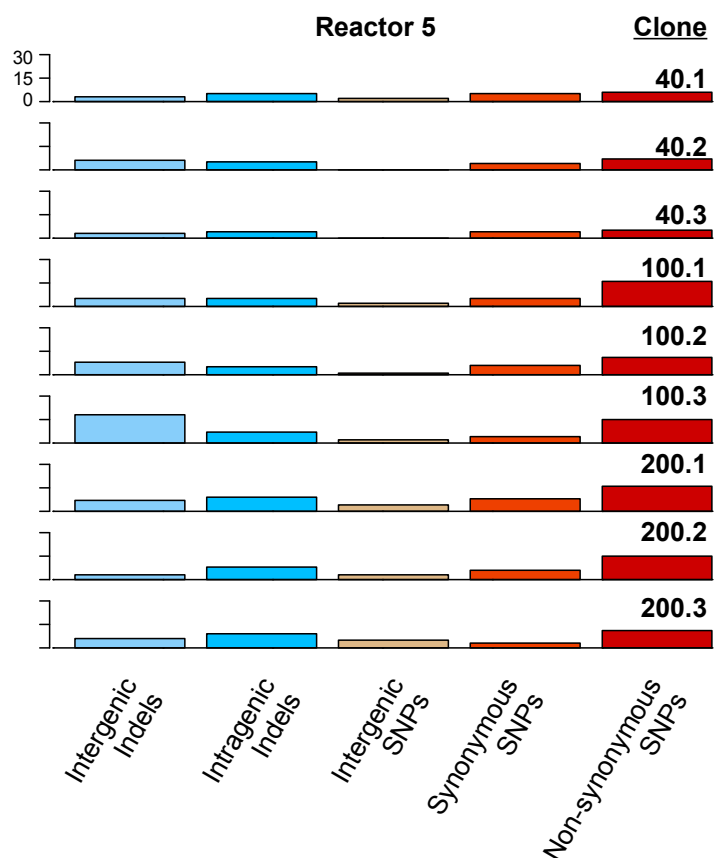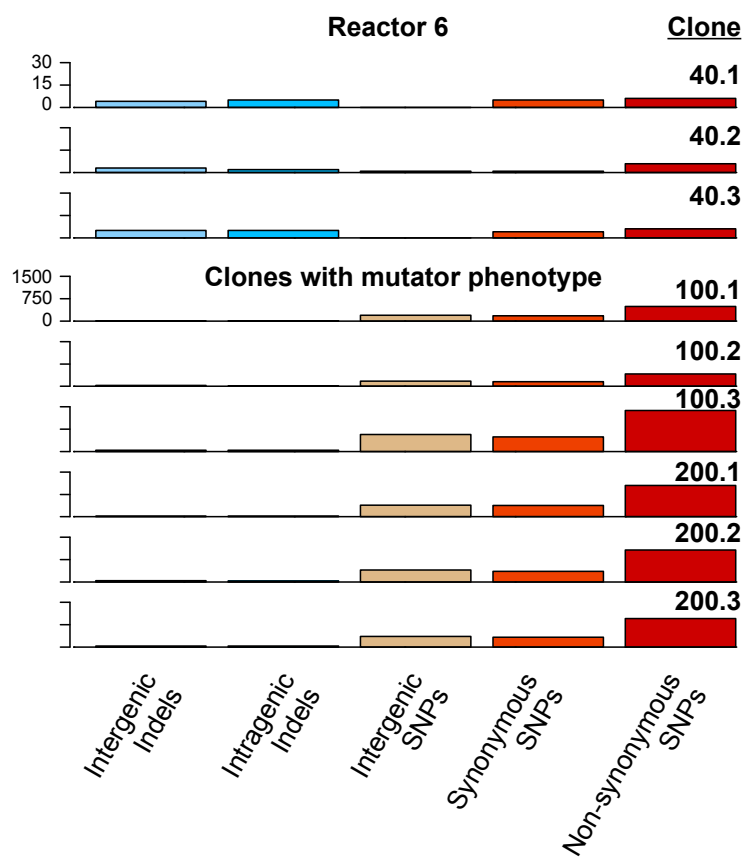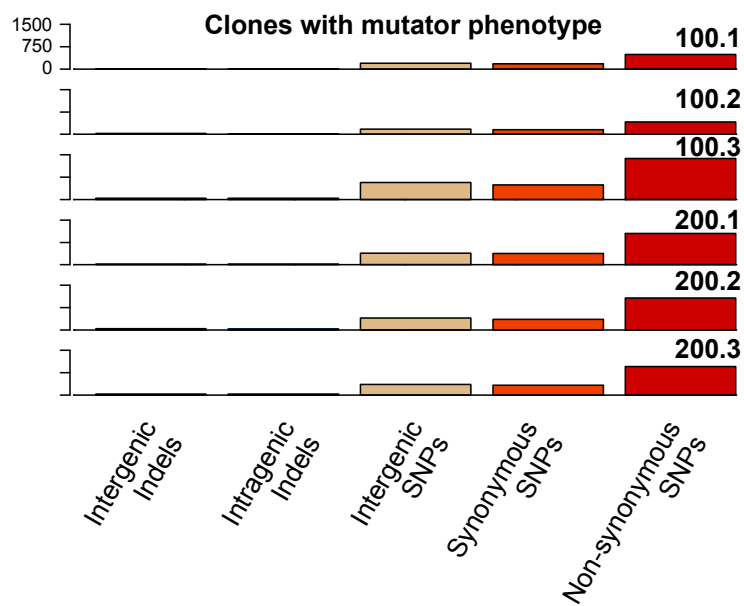

Supplement: S2 File — (PDF) [file pgen.1005635.s019.pdf]

Reactor 1

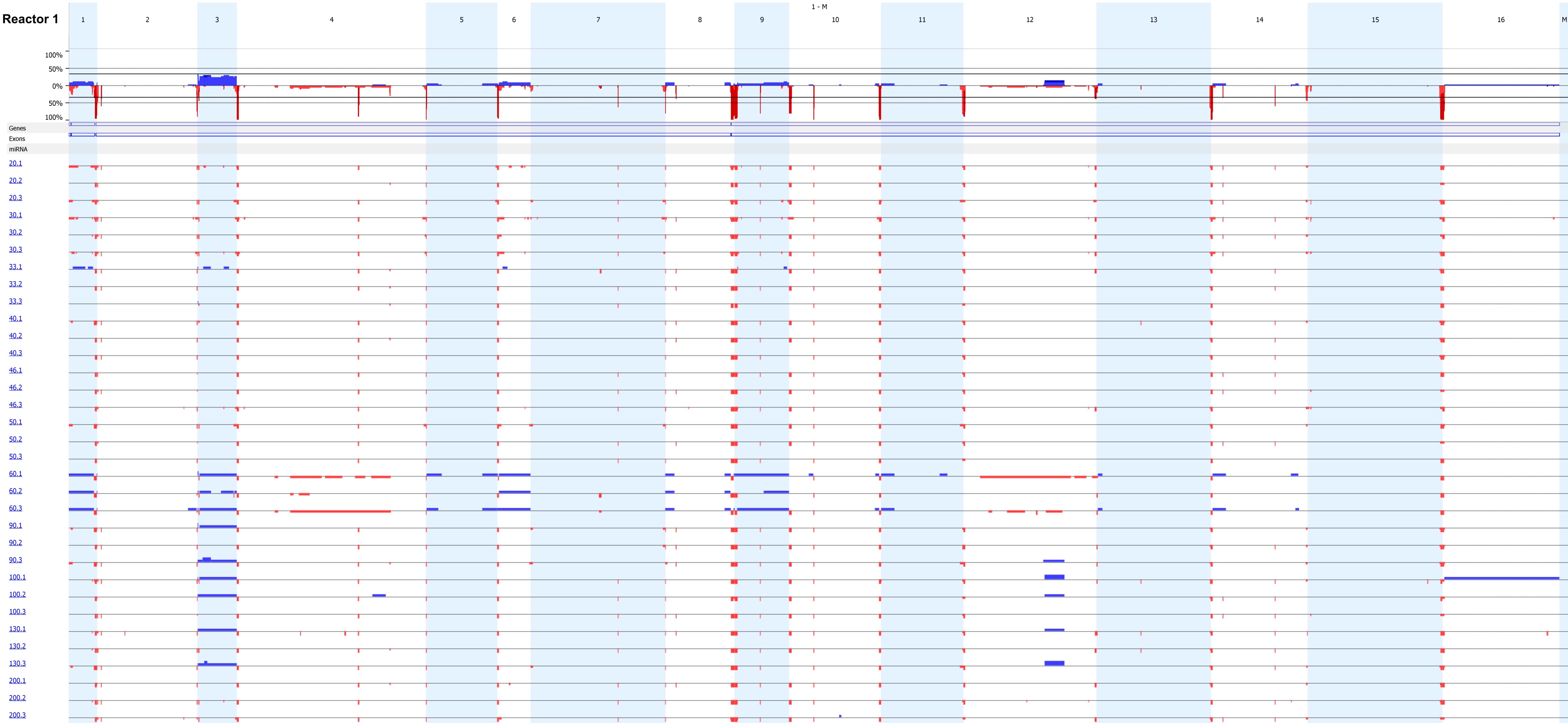

Reactor 2

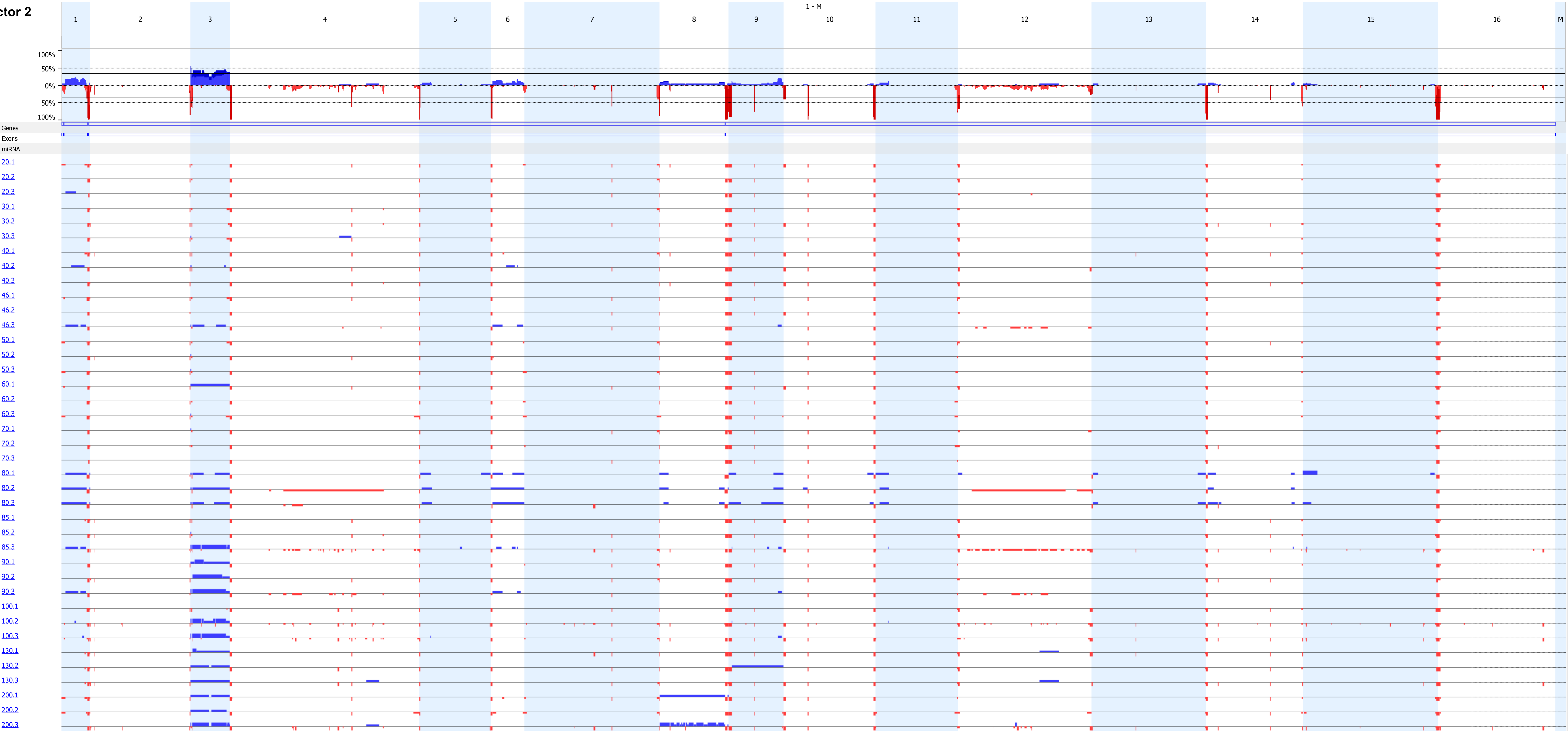

Reactor 3

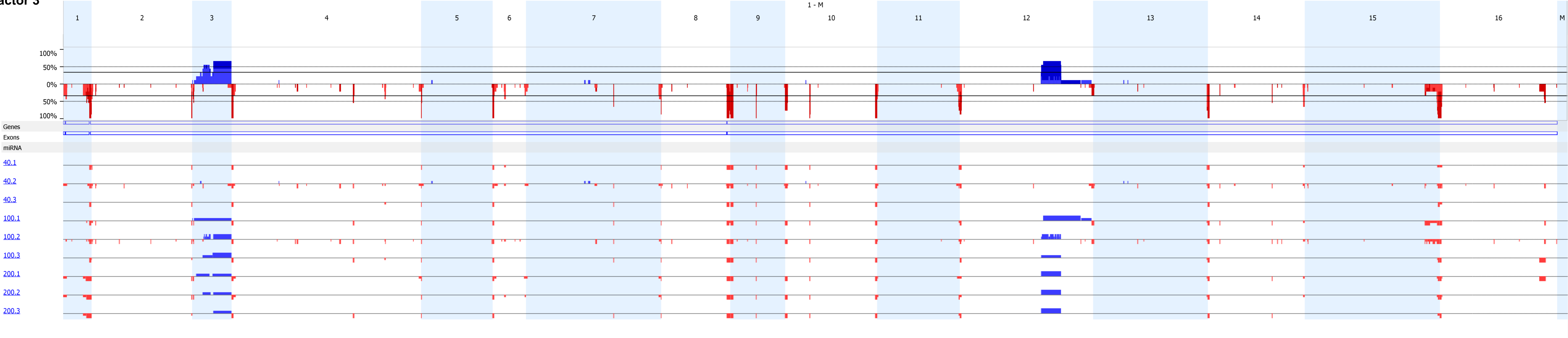

Reactor 4

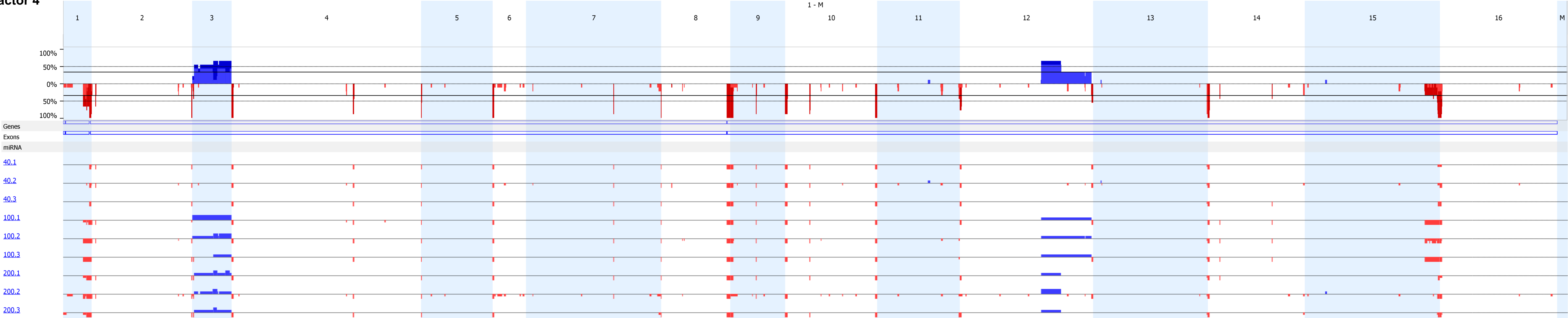

Reactor 5

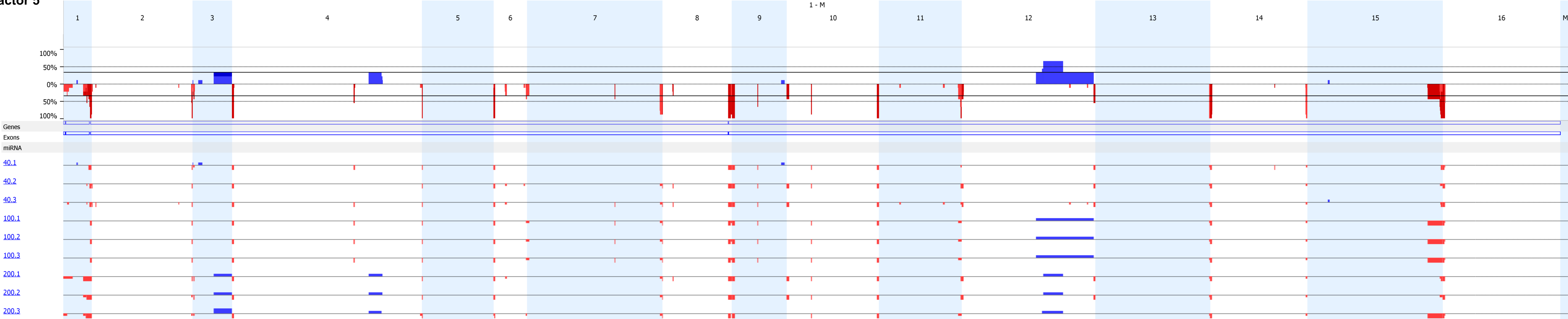

Reactor 6

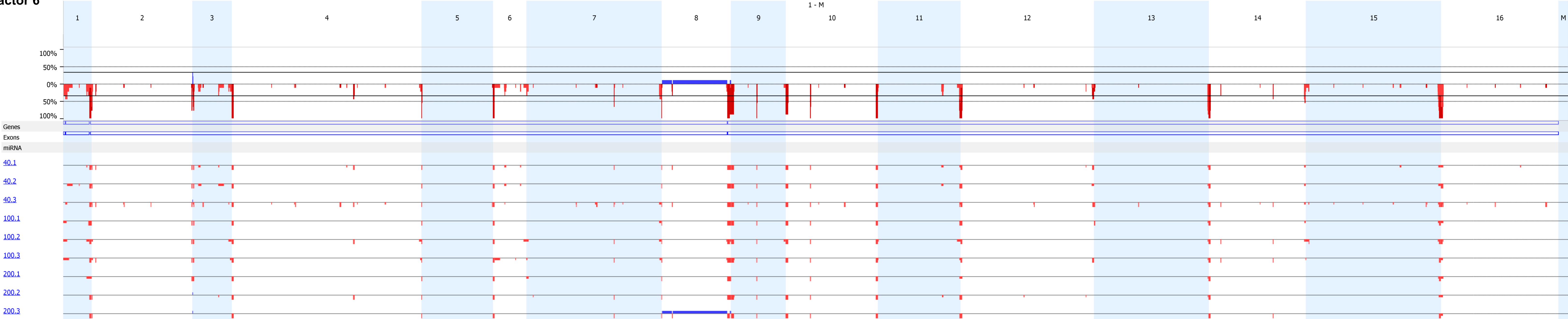

Supplement: S3 File — Copy number variations identified in evolved clones from different reactors, across the length of all yeast chromosomes (drawn to scale). Blue and red bars indicate genomic segments where we detected significant evidence of copy number gain or loss, respectively. Timepoints of experiment and isolate numbers are indicated on the left. Upper part of the plot indicates the frequency of specific CNVs in the indicated chromosomal regions, expressed as the percentage of isolated clones that harboured them. Bottom part shows the CNV patterns in all individual clones. (PDF) [file pgen.1005635.s020.pdf]
