# Supplementary material for: Adaptation to High Ethanol Reveals Complex Evolutionary Pathways
Source: PLoS Genet. 2015 Nov 6;11(11):e1005635. doi: 10.1371/journal.pgen.1005635 (PMC4636377; doi:10.1371/journal.pgen.1005635)
Supplement: S1 Text — (DOC) [file pgen.1005635.s017.doc]

**Supplementary Text**

**Phenetic analyses**

PheNetic was used to connect the mutated genes over the interaction network [1,2] with the following parameters: 100-best paths with a maximum path length of 4 were sampled between the different mutations in combination with a search tree cutoff of 0.01. As the size of the selected sub-network by PheNetic is dependent on both a cost parameter and the number of mutated genes in the input, different costs were used for the sub-network inference from different sizes of mutated gene lists. For the sub-network inference between all the mutated genes from the non-mutator reactors a cost of 0.25 was used, for the non-mutator reactors (1,3,4,5) a cost of 0.05 was used as they all have a similar amount of mutated genes, and for the mutator reactors (2 and 6) a cost of 0.5 was used.

For reactor 1, this yields a sub-network containing 73 genes connected through 113 interactions which connect 11 of the 12 mutations identified for reactor 1 that map to the interaction network. For reactor 3, the sub-network contains 76 nodes connected through 118 interactions which connect 9 of the 12 mutations. For reactor 4, the sub-network contains 93 nodes connected through 141 interactions which connect all 12 mutations. For reactor 5, the sub-network contains 100 genes connected through 157 interactions connecting 18 of 23 mutations. For the mutator phenotypes in reactors 2 and 6, we performed two analysis rounds: one with a cost of 0.05 as the others and one with 0.5 to obtain smaller networks. For reactor 2 for the large network we retrieved a sub-network that contains 301 nodes connected by 439 interactions. For the small sub-network the size is 104 nodes and 75 edges. For reactor 6 for the large network we retrieved a sub-network containing 358 nodes connected by 527 interactions. For the small network the size is 136 nodes and 97 edges.

**Interactome analyses**

For the pathway enrichment analysis, we focused on non-synonymous mutations and indels in protein coding genes present at a frequency of at least 20% in the population samples. Non-synonymous mutations and indels of different time-points of population data were analyzed together, whereas data from different reactors were kept separate. To assess the possible consequences of genetic mutations on biological pathways, we analyzed *in-silico* interactions between protein coding genes, in a reactor-wise manner using Cytoscape software. We used GeneMANIA to generate networks of interactions between input gene-sets in a genome-wise manner and also retrieve additional interacting genes with the gene-set from publicly available data [3,4]. Each set of mutated genes was subjected to interactome analysis and resulting diagrams were stored for further analysis. The false discovery rate (FDR) value was also calculated with the online version of GeneMANIA.

**Protein domain mutational analysis**

To analyze the effects of mutations on protein functional domains we investigated whether the mutation would result in an amino acid change inside a functional domain(s) of a respective protein. For this purpose, we translated the genetic mutations into the amino acid sequence. Yeast protein domain annotations were retrieved from the SMART database [5] that contains precomputed domain annotations (pfam and SMART domains). We used batch access (http://smart.embl.de/smart/batch.pl) to retrieve domain annotations for each of the mutated protein coding genes. Data was sorted using R software environment.

**Fluctuation assay**

Fluctuation assays to determine mutation rates were performed as follows: 200 µl cultures of URA3^+^cells were grown at 30°C in synthetic complete medium containing 2% glucose in 96-well plates for 48h. For each genotype, at least 40 replicates were grown. 36 cultures were then plated on FOA plates (1g/L) for mutant selection and incubated at 30°C for 2 days, after which colonies were counted. 4 cultures were diluted and plated on YPD to determine total cell counts. Mutation rates and their corresponding 95% confidence intervals were determined by using the Ma-Sandri-Sarkar maximum likelihood method [6] as implemented in the Fluctuation Analysis Calculator [7]. Mutation rates were obtained as mutation rate (mutations/generation) and were converted to mutation rate (mutations/bp/generations) based on the study performed by Lang and Murray [8].

**References**

1. De Maeyer D, Renkens J, Cloots L, De Raedt L, Marchal K (2013) PheNetic: network-based interpretation of unstructured gene lists in E. coli. Mol Biosyst 9: 1594-1603.

2. De Maeyer D, Weytjens B, Renkens J, De Raedt L, Marchal K (2015) PheNetic: network-based interpretation of molecular profiling data. Nucleic Acids Res.

3. Montojo J, Zuberi K, Rodriguez H, Kazi F, Wright G, et al. (2010) GeneMANIA Cytoscape plugin: fast gene function predictions on the desktop. Bioinformatics 26: 2927-2928.

4. Mostafavi S, Ray D, Warde-Farley D, Grouios C, Morris Q (2008) GeneMANIA: a real-time multiple association network integration algorithm for predicting gene function. Genome Biol 9 Suppl 1: S4.

5. Schultz J, Milpetz F, Bork P, Ponting CP (1998) SMART, a simple modular architecture research tool: identification of signaling domains. Proc Natl Acad Sci U S A 95: 5857-5864.

6. Sarkar S, Ma WT, Sandri GH (1992) On fluctuation analysis: a new, simple and efficient method for computing the expected number of mutants. Genetica 85: 173-179.

7. Hall BM, Ma CX, Liang P, Singh KK (2009) Fluctuation analysis CalculatOR: a web tool for the determination of mutation rate using Luria-Delbruck fluctuation analysis. Bioinformatics 25: 1564-1565.

8. Lang GI, Murray AW (2008) Estimating the per-base-pair mutation rate in the yeast Saccharomyces cerevisiae. Genetics 178: 67-82.
