# Supplementary material for: Adaptation to High Ethanol Reveals Complex Evolutionary Pathways
Source: PLoS Genet. 2015 Nov 6;11(11):e1005635. doi: 10.1371/journal.pgen.1005635 (PMC4636377; doi:10.1371/journal.pgen.1005635)
Supplement: S1 Table — This table lists all genes hit multiple times in different reactors and populations (genes hit at different positions; mutations identical at the nucleotide level are excluded from this analyses). (DOC) [file pgen.1005635.s025.doc]

**Table S1. Genes hit multiple times across reactors and populations**

| **Gene** | **Number of mutations** | **Reactors hit** | **Genomic positions of Mutations** | **Gene length** | **Proportion** | **P-value** | **Significant (0.05)** |
| --- | --- | --- | --- | --- | --- | --- | --- |
| *DSK2* | 6 | 5 | 819599_F1,819639_F1,819768_F2,819322_F3,819841_F4,818829_F5 | 1122 | 0.0001 | 0.0000 | yes |
| *FMP27* | 5 | 3 | 1048651_F1,1046662_F2,1045920_F6,1048159_F6,1051252_F6 | 7887 | 0.0009 | 0.0007 | yes |
| *ASG1* | 5 | 4 | 103160_F1,104223_F1,104223_F2,103484_F3,104223_F5 | 2895 | 0.0003 | 0.0000 | yes |
| *LAA1* | 4 | 2 | 47026_F2,47026_F2,41891_F6,43086_F6 | 6045 | 0.0007 | 0.0021 | yes |
| *HEM3* | 4 | 3 | 93443_F2,93443_F2,93355_F5,93084_F6 | 984 | 0.0001 | 0.0000 | yes |
| *ACE2* | 3 | 3 | 406519_F1,405266_F4,405008_F6 | 2313 | 0.0003 | 0.0012 | yes |
| *POL3* | 3 | 2 | 279361_F5,278116_F6,278707_F6 | 3294 | 0.0004 | 0.0032 | yes |
| *RAP1* | 3 | 3 | 243501_F3,243486_F5,242678_F6 | 2484 | 0.0003 | 0.0015 | yes |
| *PUF4* | 2 | 2 | 467449_F2,468175_F6 | 2667 | 0.0003 | 0.0226 | yes |
| *YJL070C* | 2 | 2 | 309562_F5,308493_F6 | 2667 | 0.0003 | 0.0226 | yes |
| *CSE1* | 2 | 2 | 51180_F4,51880_F6 | 2883 | 0.0003 | 0.0259 | yes |
| *RGC1* | 2 | 2 | 756890_F2,755613_F6 | 3252 | 0.0004 | 0.0319 | yes |
| *EPS1* | 2 | 2 | 347776_F2,346118_F6 | 2106 | 0.0002 | 0.0148 | yes |
| *GFA1* | 2 | 2 | 243930_F5,244220_F6 | 2154 | 0.0002 | 0.0155 | yes |
| *UTH1* | 2 | 2 | 520589_F1,519947_F4 | 1098 | 0.0001 | 0.0044 | yes |
| *GRR1* | 2 | 2 | 593831_F2,592481_F6 | 3456 | 0.0004 | 0.0354 | yes |
| *YER156C* | 2 | 2 | 484119_F2,483437_F6 | 1017 | 0.0001 | 0.0038 | yes |
| *JID1* | 2 | 2 | 676633_F2,676880_F6 | 906 | 0.0001 | 0.0031 | yes |
| *YJL182C* | 2 | 2 | 85527_F2,85721_F6 | 318 | 0.0000 | 0.0004 | yes |
| *RIM15* | 2 | 2 | 69216_F4,71799_F6 | 5313 | 0.0006 | 0.0708 | no |
| *IRA2* | 2 | 2 | 176606_F2,177311_F6 | 9240 | 0.0010 | 0.1505 | no |
| *ATG11* | 2 | 2 | 662461_F2,662396_F6 | 3537 | 0.0004 | 0.0368 | yes |
| *TRS130* | 2 | 2 | 706550_F2,705442_F6 | 3309 | 0.0004 | 0.0329 | yes |
| *HEM12* | 2 | 2 | 552937_F1,552937_F4 | 1098 | 0.0001 | 0.0044 | yes |
| *HRK1* | 2 | 2 | 822924_F2,823076_F6 | 2280 | 0.0003 | 0.0171 | yes |
| *DPB11* | 2 | 2 | 264037_F2,263060_F6 | 2295 | 0.0003 | 0.0173 | yes |
| *UBR1* | 2 | 2 | 864226_F2,864100_F6 | 5853 | 0.0006 | 0.0819 | no |
| *VPS74* | 2 | 2 | 1221649_F2,1221747_F6 | 1038 | 0.0001 | 0.0040 | yes |
