# Supplementary material for: Adaptation to High Ethanol Reveals Complex Evolutionary Pathways
Source: PLoS Genet. 2015 Nov 6;11(11):e1005635. doi: 10.1371/journal.pgen.1005635 (PMC4636377; doi:10.1371/journal.pgen.1005635)
Supplement: S2 Table — (DOCX) [file pgen.1005635.s026.docx]

**Table S2. Haplotype frequencies of reactor 1 and 2**

|  | **Reactor 1 haplotype** | **0** | **30** | **33** | **40** | **46** | **50** | **60** | **80** | **90** | **100** | **130** | **200** |
| --- | --- | --- | --- | --- | --- | --- | --- | --- | --- | --- | --- | --- | --- |
| 1 | ASG1, ACE2 | 0.00 | 0.00 | 0.00 | 0.00 | 0.00 | 0.00 | 0.00 | 0.81 | 0.50 | 0.00 | 0.00 | 0.00 |
| 2 | FMP27, VPS70, HST4, ASG1, MCM2, DSK2 | 0.00 | 0.00 | 0.00 | 0.00 | 0.00 | 0.00 | 0.00 | 0.00 | 0.00 | 0.00 | 0.74 | 0.88 |
| 3 | HEM12 | 0.00 | 0.00 | 0.00 | 0.00 | 0.00 | 0.00 | 0.00 | 0.00 | 0.17 | 0.83 | 0.00 | 0.00 |
| 4 | HYM1 | 0.00 | 0.00 | 0.17 | 0.14 | 0.10 | 0.16 | 0.08 | 0.03 | 0.23 | 0.09 | 0.00 | 0.00 |
| 5 | RPA190 | 0.00 | 0.00 | 0.00 | 0.00 | 0.00 | 0.00 | 0.00 | 0.13 | 0.51 | 0.00 | 0.00 | 0.00 |
|  |  |  |  |  |  |  |  |  |  |  |  |  |  |
|  | **Reactor 2 haplotype** | **0** | **30** | **40** | **50** | **60** | **70** | **80** | **85** | **90** | **100** | **130** | **200** |
| 1 | PDE2 | 0.00 | 0.17 | 0.27 | 0.28 | 0.15 | 0.27 | 0.50 | 0.10 | 0.00 | 0.00 | 0.00 | 0.00 |
| 2 | BNI1, PET123 | 0.00 | 0.00 | 0.00 | 0.00 | 0.00 | 0.00 | 0.00 | 0.00 | 0.05 | 0.07 | 0.14 | 0.96 |
| 3 | IRA2, KAR2, SDO1, DSF2, ARG7, RPG1, SPT7, | 0.00 | 0.00 | 0.00 | 0.00 | 0.00 | 0.00 | 0.00 | 0.45 | 0.73 | 0.79 | 0.96 | 0.96 |
|  | EXO84, GMH1, GRR1, ENP1, CRM1, MAG2 |  |  |  |  |  |  |  |  |  |  |  |  |
| 4 | CDC27 | 0.00 | 0.00 | 0.00 | 0.00 | 0.00 | 0.00 | 0.00 | 0.00 | 0.00 | 0.83 | 0.98 | 0.00 |
| 5 | RGC1, YBL059W, PCA1, WTM1, DSK2 | 0.00 | 0.00 | 0.00 | 0.00 | 0.00 | 0.01 | 0.05 | 0.66 | 0.84 | 0.83 | 0.99 | 0.99 |
| 6 | ASG1, MSH2 | 0.00 | 0.00 | 0.00 | 0.00 | 0.00 | 0.00 | 0.15 | 0.86 | 0.98 | 1.00 | 0.99 | 1.00 |
