# Supplementary material for: Adaptation to High Ethanol Reveals Complex Evolutionary Pathways
Source: PLoS Genet. 2015 Nov 6;11(11):e1005635. doi: 10.1371/journal.pgen.1005635 (PMC4636377; doi:10.1371/journal.pgen.1005635)
Supplement: S3 Table — Specific pathways hit in the different evolved populations, with FDR values. Mutated genes are the number of genes of this specific pathway mutated in the specific reactor, genes present in genome is the total number of genes in the genome for a particular pathway. As reactors 2 and 6 are mutators for Indels and SNPs respectively, we analyzed enrichments separately for SNPs and Indels to be able to compare between populations. (DOCX) [file pgen.1005635.s027.docx]

|  | **Population - SNP** |  |  |  |
| --- | --- | --- | --- | --- |
|  | Pathways | FDR | Mutated genes | Genes present in genome |
| Reactor 1 | Budding cell growth | 6.5e-5 | 5 | 21 |
|  | Cytokinetic cell separation | 6.5e-5 | 5 | 51 |
| Reactor 2 | Negative regulation of small  GTPase medicated signal  transduction | 3.7e-4 | 4 | 12 |
| Reactor 3 | Mitochondrial ribosome  Organelle ribosome | 1.0e-13  1.0e-13 | 13  13 | 84  84 |
| Reactor 4 | No significant enrichment found | - | - | - |
| Reactor 5 | DNA replication  DNA repair | 6.3e-4  1.7e-7 | 14  14 | 144  237 |
| Reactor 6 | No significant enrichment found | - | - | - |
|  | **InDels** |  |  |  |
| Reactor 1 | Multi-eIF complex  Translation initiation factor activity | 1.9e-12  1.9e-12 | 5  7 | 10  29 |
| Reactor 2 | No significant enrichment found | - | - | - |
| Reactor 3 | No significant enrichment found | - | - | - |
| Reactor 4 | U2-type spliceosomal complex  Spliceosomal complex | 6.66e-12  6.66e-12 | 10  10 | 54  60 |
| Reactor 5 | SCF ubiquitin ligase complex  Ubiquitin ligase complex | 2.4e-20  2.4e-20 | 11  11 | 19  19 |
| Reactor 6 | 90S pre-ribosome  snoRNA binding | 7.6e-5  7.6e-5 | 7  12 | 12  90 |

**Table S3.** **Interactome analyses**
