# Supplementary material for: Adaptation to High Ethanol Reveals Complex Evolutionary Pathways
Source: PLoS Genet. 2015 Nov 6;11(11):e1005635. doi: 10.1371/journal.pgen.1005635 (PMC4636377; doi:10.1371/journal.pgen.1005635)
Supplement: S4 Table — (DOC) [file pgen.1005635.s028.doc]

**Table S4. List of strains used in this study**

| **Name** | **Genotype** | **Reference** |
| --- | --- | --- |
| KV172 | prototrophic haploid S288c, mating type α | FY5 from [1] |
| KV1574 | wt-mCherry-HYG | [2] |
| KV1575 | wt-Citrine-HYG | [2] |
| VK111 | KV172 *flo1Δ flo10Δ flo11Δ* | This study, haploid ancestral strain |
| VK145 | a/α *flo1Δ flo10Δ flo11Δ* | This study, diploid ancestral strain |
| VK202 | aa/αα *flo1Δ flo10Δ flo11Δ* | This study, tetraploid ancestral strain |
| TV70 | VK111 *TDH3*p-YECitrine - HYG | This study |
| TV92 | VK111 *TDH3*p-mCherry -HYG | This study |
| VK2925 | VK111 *msh2*::HYG | This study |
| VK2992 | VK111 *msh2*:: *msh2inde*l-HYG | This study |
| MVP6 | VK111 *TDH3*p-mCherry | This study |
| MVP109 | VK111 *TDH3*p-YECitrine | This study |
| MVP197 | MVP6 *ybl059w^G479T^* loxP-*HYG*-loxP | This study |
| MVP242 | MVP6 chrIV:1489310^A>T -^loxP-*HYG*-loxP | This study |
| MVP252 | MVP109 chrIV:1489310^A>T-^ loxP-*HYG*-loxP | This study |
| MVP269 | MVP6 *hem13^G700C^* loxP-*HYG*-loxP | This study |
| MVP302 | MVP109 chrXII:747403^C>T^  loxP-*HYG*-loxP | This study |
| MVP303 | MVP109 *hem13^G700C^* loxP-HYG-loxP | This study |
| MVP314 | MVP109 *hst4^G262C^*  loxP-*HYG*-loxP | This study |
| MVP322 | MVP6 *prt1^A1384G^* loxP-*HYG*-loxP | This study |
| MVP323 | MVP109 *prt1^A1384G^* loxP-*HYG*-loxP | This study |
| MVP325 | MVP6 *vps70^C595A^* loxP-*HYG*-loxP | This study |
| MVP328 | MVP109 *vps70^C595A^* loxP-*HYG*-loxP | This study |
| MVP350 | MVP109 *mex67^G456A^* loxP-*HYG*-loxP | This study |
| MVP395 | MVP6 *hst4^G262C^*  loxP-*HYG*-loxP | This study |
| MVP442 | MVP109 *ybl059w^G479T^* loxP-*HYG*-loxP | This study |
| MVP495 | MVP6 chrXII:747403^C>T^ + loxP-*HYG*-loxP | This study |
| MVP573 | MVP6 *pca1^C1583T^* loxP-HYG-loxP | This study |
| MVP590 | MVP109 *pca1^C1583T^* loxP-HYG-loxP | This study |

**References**

1. Brachmann CB, Davies A, Cost GJ, Caputo E, Li J, et al. (1998) Designer deletion strains derived from Saccharomyces cerevisiae S288C: a useful set of strains and plasmids for PCR-mediated gene disruption and other applications. Yeast 14: 115-132.

2. Smukalla S, Caldara M, Pochet N, Beauvais A, Guadagnini S, et al. (2008) FLO1 is a variable green beard gene that drives biofilm-like cooperation in budding yeast. Cell 135: 726-737.
