# Supplementary material for: Adaptation to High Ethanol Reveals Complex Evolutionary Pathways
Source: PLoS Genet. 2015 Nov 6;11(11):e1005635. doi: 10.1371/journal.pgen.1005635 (PMC4636377; doi:10.1371/journal.pgen.1005635)
Supplement: S5 Table — (DOCX) [file pgen.1005635.s029.docx]

**Table S5. List of primers used**

| **N°** | **Name** | **Sequence** |
| --- | --- | --- |
| 1 | FLO1_KO_Fw | TTCCGGGTTCTTATTTTTAATTCTTGTCACCAGTAAACAGAACATCCAAACAGCTGAAGCTTCGTACGC |
| 2 | FLO1_KO_Rv | TAAGAAGCGCAAGAATTATCATTTAGTCAATTTGAATATTTGAAAGTATGGAGGGCATAGGCCACTAGTGGATCTG |
| 3 | FLO10_KO_Fw | TTACGTTGAAGATTTGTTTTAGGGTGCTTAATCAAAGAACAACAAATAAAAACAGCTGAAGTTCGTACGC |
| 4 | FLO10_KO_Rv | GACGAATCGTAGACGCAGAAGTATCAATCCAAAGGATATTTCTGCACCTAGCATAGGCCACTAGTGGATCTG |
| 5 | FLO11_KO_Fw | TTCTAATTAAAATATACTTTTGTAGGCCTCAAAAATCCATATACGCACACTCAGCTGAAGCTTCGTACGC |
| 6 | FLO11_KO_Rv | ATTTAAGAATGAAAACATCGTAATGAAGAAACGAACATGTTGGAATTGTATCAGCATAGGCCACTAGTGGATCTG |
| 7 | FLO1_UPSTR_Fw | ATAGGGAGGCATCATGGT |
| 8 | FLO10_UPSTR_Fw | GTTGTTGTGATCCGTCAC |
| 9 | FLO11_UPSTR_Fw | GATTCAAGGCATCATCGC |
| 3550 | FLO1_ck_Fw | ATAGGGAGGCATCATGGT |
| 3551 | FLO1_ck_Rv | TTTTGGTGCACTTTTACACC |
| 3554 | FLO10_ck_Fw | GTTGTTGTGATCCGTCAC |
| 3555 | FLO10_ck_Rv | CAGCTGTTGGTTGACTTTATG |
| 3558 | FLO11_ck_Fw | GATTCAAGGCATCATCGC |
| 3559 | FLO11_ck_Rv | TGTCGTTCTATCTCGCGAAT |
| 2821 | URA3_WT-ko(f) | GTGAAGGATAAGTTTTGACCATCAAAGAAGGTTAATGTGGCTGTGGTTTCAGGGTCCATA-CAGCTGAAGCTTCGTACGCTGCAGG |
| 2822 | URA3_WT-ko(r) | AAGCTTTTTCTTTCCAATTTTTTTTTTTTCGTCATTATAGAAATCATTACGACCGAGATT-CGCATAGGCCACTAGTGGATCTG |
| 3143 | CHK-URA3-F | CTAGGGAAGACAAGCAACG |
| 3340 | CHK-insURA-R | GGTTAATGTCGCTTTTGGCA |
| 69 | KanMx_Rv | GCACGTCAAGACTGTCAAGG |
| 86 | Mata_Fw | ACTCCACTTCAAGTAAGAGTTTG |
| 87 | Matalpha_Fw | GCACGGAATATGGGACTACTTCG |
| 88 | Matloc_Rv | AGTCACATCAAGATCGTTTATGG |
| 147 | HYG_Rv | TCGACAGACGTCGCGGTGAGTT |
| 1920 | YRO-CONT-FW | CCTACCCGGTTGCCTCCAAAGCCCTTCTTT |
| 1923 | YRO-CONT-RV | AACTAGGTTACATAACTGCCTATTGGCAAG |
| 3041 | check-YRO2-f | GGCAACCATTCTCCAACATT |
| 4597 | Yro2_fl_KanMx_Fw | TATTGACCACACCTCTACCGGCAGATCCGCTAGGGATAACAGGGTAATATAGATCTGTTTCAGCTGAAGCTTCGTACGC |
|  |  |  |
| 4598 | Yro2_fl_KanMx_Rv | GAGGCCGATGGCAGCAACCCTAGTCGACACTGGATGGCGGCGTTAGTATCGAATCGACAGGCATAGGCCACTAGTGGATCTG |
|  |  |  |
| JC9 | pCB1_downstrHYG_Fw | TGGTCGCTATACTGCTGTCG |
| JC26 | HYG_YRO2_RV | TTTGAAGACGACAAGGAAAGC |
| JC93 | pCB1_upstreamloxP_Fw | CAGCTGAAGCTTCGTACGC |
| JC101 | TDH3_Fw | CTTTTTAAGCTGGCATCCAG |
| JC110 | pCB1_loxP_Fw | ATCTGTTTAGCTTGCCTCGT |
| 4599 | PCA1insertmarker_Fw | GACGACTCTGTAATACTCTATTTATTATTTAGTTTAGTTTTCTATTTAATATGATGGAATCAGCTGAAGCTTCGTACGC |
| 4600 | PCA1insertmarker_Rv | TAATTTATCAGTTGATCTGCAATTACCTGGCATGGCTATGTGTATTTTTTTTTTTTATGAGCATAGGCCACTAGTGGATCTG |
| 4601 | ckinsertmarkerPCA1_Fw | GCCAAGCTAGCTGCTGATGT |
| 4602 | ckinsertmarkerPCA1_Rv | CAACATTAGGTAGAACTTTTATTGCTCAG |
|  | PCA1insertSNP_Fw | AATATCATTCGTATTTACTTTGACCCAAAGGTTATAGGTgtcAGAGATCTTGTCAATGAAGGATGGAGCGTGCCTGTTAGT |
|  | PCA1checkrecombSNP_Fw | GCTCAGGGATCTGTCAAGG |
| 4644 | IV1489310insertmarker_Fw | TCGTCAAGTACTATTATAACGACAGCGATAGGAAAAAAAACCAACATCGGACGAGAATAACAGCTGAAGCTTCGTACGC |
| 4645 | IV1489310insertmarker_Rv | ATCGCGGAAAATTTCATATCGAATTTTAAAGGAATGTCATTTTGAACGCTAAAGTATTATGCATAGGCCACTAGTGGATCTG |
| 4646 | IV1489310checkmarker_Fw | GCTACCTTAACCACTCTGC |
| 4647 | IV1489310checkmarker_Rv | GTCGTATTCTCGGCGAATTA |
| 4648 | IV1489310insertSNP_Rv | TTTATTTTTATTTCCTCTCTATAATTATTACCTGTATTAAaTCATTATCTAATATAAAATCTAATCTAATAATAGAATTTT |
| 4697 | IV1489310checkrecombSNP_Rv | CCGGTGTCGCTTTCTTATTC |
| 4649 | XII747403insertmarker_Fw | TACTTTTCTATTGCAACGGCCTAAAGGAATTCGATAAAAGAAAGAGTAGGCAAGATAAGCCAGCTGAAGCTTCGTACGC |
| 4650 | XII747403insertmarker_Rv | CAATGGAAGAACAAAGAAAATTTAGCGGAAGTAAAAATAACAGCCGAAAGCCAAATTCAGGCATAGGCCACTAGTGGATCTG |
| 4651 | XII747403checkmarker_Fw | GCACCTGATGCGCATAATAG |
| 4652 | XII747403checkmarker_Rv | CCCTGACGCATGTTTCTCAT |
| 4653 | XII747403insertSNP_Fw | AACTGAATAATTTTCTTGTCTGCGTTCCTTTTTTTGTGCAtCAATGGATGTAGTATGCGCACCTGATGCGCATAATAGATT |
| 4746 | XII747403checkrecombSNP_Fw | GTAGTGGAAAAGCAGGTGTG |
| 4654 | HEM13insertmarker_Fw | TCGACCACTTATTTATACATTTTTTTATCTATTTATTATACCTCCTTTACATAATTCTTCCAGCTGAAGCTTCGTACGC |
| 4655 | HEM13insertmarker_Rv | CATAAATAGGACGCAATAATTAATATTTTTAAGAATAGAGATCCTGTATCAATTGAATTTGCATAGGCCACTAGTGGATCTG |
| 4656 | HEM13checkmarker_Fw | CCAGGCTCTAGAGTTGAGTC |
| 4657 | HEM13checkmarker_Rv | GAGAATGAAGGCTGCTGCC |
| 4658 | MVP-HEM13insertSNP_Fw | CCCACAAGAAATATTGAAGATGGTTGAAGACTGTTTCGATcctTTCTTGCCATCCTACTTGACTATCGTCAAGAGAAGAAA |
| 4747 | HEM13checkrecombSNP_Fw | GGACAAGCACGACACTGC |
| 4659 | HST4insertmarker_Fw | GTTTTGCCATATTCTTCCTTGAACAAAATTGAAAAATTTTAAAAAACCTCTCCATTACCCCAGCTGAAGCTTCGTACGC |
| 4660 | HST4insertmarker_Rv | CTAAAGTTGCTGTTTTCCCCTTTTAGGAAACGTAATTGATTTTTTGCTCACGTGCTCTGCGCATAGGCCACTAGTGGATCTG |
| 4661 | HST4checkmarker_Fw | GGCCCAGAACTTAACAGCC |
| 4662 | MVP-HST4checkmarker_Rv | CCGTACTTGGTGGGGTGA |
| 4663 | HST4insertSNP_Rv | GCCCCGCTGACTACAACCATTCTTTTGCTATAATTCAGaggATAGCTAATAAAACCGGCGTCGCGGTCCATATGATGGCGT |
| 4748 | HST4checkrecombSNP_Fw | CCTAGAAGATTGCTACCGCA |
| 4679 | MEX67insertmarker_Fw | GCTACTGTTTTCGTCACGTTCCGAACAGCAGCATGCTTTGTATATTTTGTACTAATTCATCAGCTGAAGCTTCGTACGC |
| 4680 | MEX67insertmarker_Rv | CATATCTATATTTTGAAAAAATCTTCTATATAACGGTTGTATAGAAATAATTACTTATTTGCATAGGCCACTAGTGGATCTG |
| 4681 | MEX67checkmarker_Fw | GCATGAATGGCATCCCTAGA |
| 4682 | MEX67checkmarker_Rv | CCCATGCATTGGTACTCTGA |
| 4683 | MEX67insertSNP_Fw | TTAGTTCCATCTCCACCCAGTCTAAGATGTTTCCAGCAataATGAAACTGGCTTCTACAGAAAAAAGTTTGATAGTGGAAA |
| 4696 | MEX67checkrecombSNP_Fw | GGAACGGTGTCCGGTTTG |
| 4664 | PRT1insertmarker_Fw | CTTACGGGCTTGTATGTAAACTAACAACAATATCTAGTATAAGTATGAGAATTAGTCTAATCAGCTGAAGCTTCGTACGC |
| 4665 | PRT1insertmarker_Rv | CGAGGAGAGCGACCCCTGCAGATTACAAAGATAATAGAGCCTATTGTAGTATTTATGAATGCATAGGCCACTAGTGGATCTG |
| 4666 | PRT1checkmarker_Fw | CCAGAAGATGCCAGCGAT |
| 4667 | PRT1checkmarker_Rv | GTCCAATCTCTCACAGTGACT |
| 4668 | PRT1insertSNP_Fw | GTTCCTGTGCTTCAATGTCGAACGTCACACAAAGTCTGGTgagACTCAATTCAGTAATCTACAAATTTGTAGGTTGACTGA |
| 4694 | PRT1checkrecombSNP_Fw | CCATTCAGAAACGGTGACGA |
| 4669 | VPS70insertmarker_Fw | TATTTACAGAATTAAAGCTTAGAACTATCATAACTTTCATAATATACCACCAGGCTTCAACAGCTGAAGCTTCGTACGC |
| 4670 | VPS70insertmarker_Rv | GTCAAACCTTTTTACCCGGGCCATCAAACTCAAGCAAACTCCGAATGGAACAATGCCTTTGCATAGGCCACTAGTGGATCTG |
| 4671 | VPS70checkmarker_Fw | GGCGGCCGTCTCTATACT |
| 4672 | VPS70checkmarker_Rv | CGAGTGGTCTCGCCACTA |
| 4673 | VPS70insertSNP_Rv | ATCATGCTTGCTTCGTAGACCACTTTACCATTTTCTAAagtAGCTACGTTAGTGTCTACTGGTTCACCTATCCATGGGTAA |
| 4695 | VPS70checkrecombSNP_Rv | CCGATCGTACGATGTGGATT |
| 4674 | YBL059Winsertmarker_Fw | TAGTAGTAGTTTCAATAATATATTCCACTACTTATATGTGTTACCCGCATTAGAACTCTTCAGCTGAAGCTTCGTACGC |
| 4675 | YBL059Winsertmarker_Rv | ATTGGTGGCGAAAATCGATGGCAATAAAGAACGGAAGGGGTTTAATAGTTGTATGCTTAAGCATAGGCCACTAGTGGATCTG |
| 4676 | YBL059Wcheckprimer_Fw | GGATAAAAGAAGCCAGCAAGT |
| 4677 | YBL059Wcheckprimer_Rv | TATAAGAAACGTCGGTAGCAC |
| 4678 | YBL059WinsertSNP_Fw | TTAGCGGCAACGGGCATGACACTAGGTATATTTGGTATGGtCATCACAGGGACATGTTGGAGCTGGGATGTTTCATCATTT |
| 4698 | YBL059WcheckrecombSNP_Fw | CGGCCTCATACTCAAAGAAC |
| 4504 | Msh2plusHYG-Fw2 | TTAATATTACAACGACATCTTAAGTGAGAATCGATAGATAATATATAGATACAAATAGTACAGCTGAAGCTTCGTACGC |
| 4505 | Msh2plusHYG-Rv2 | CTGTACCTTGTCTACAAAGATTGTAAAAAATAAAAATTCTTTCCCAATGCATATTATATGGCATAGGCCACTAGTGGATCTG |
| 4524 | MSH2DEL_Fw | ATCAACTGTAAAAAATCTCTTTATCTGCTGACCTAACATCAAAATCCTCAGATTAAAAGTCAGCTGAAGCTTCGTACGC |
| 4525 | MSH2DEL_Rv | TACTATTTGTATCTATATATTATCTATCGATTCTCACTTAAGATGTCGTTGTAATATTAAGCATAGGCCACTAGTGGATCTG |
| 4552 | MSH2insertevolved_Fw | AAGTTGGAGAACCATCATCTGCATGGTTGGTGTATGAGGTTGACACGTAATGACGCCAAG**GAGTTACGTAATGACGCCAAG**GAGTTACGTAAACATAAGAAGTACATTGAG |
| 4489 | seqMsh2-p1 | GAAGAAATGGTTGAAACAACG |
| 4490 | seqMsh2-p2 | ATGAGCAAAAGAGGCAATCA |
