# Supplementary material for: Adaptation to High Ethanol Reveals Complex Evolutionary Pathways
Source: PLoS Genet. 2015 Nov 6;11(11):e1005635. doi: 10.1371/journal.pgen.1005635 (PMC4636377; doi:10.1371/journal.pgen.1005635)
Supplement: S6 Table — (DOC) [file pgen.1005635.s030.doc]

**Table S6.** **Competitive fitness of site-directed mutant strains ^a^**.

| **Ethanol (v/v)** | **0%** | | **4%** | | **6%** | | **8%** | |
| --- | --- | --- | --- | --- | --- | --- | --- | --- |
|  | *s* | ± | *s* | ± | *s* | ± | *s* | ± |
| *YECitrine* ^b^ |  |  |  |  |  |  |  |  |
| *mut1* | 0.006 | 0.001 | 0.009 | 0.003 | 0.006 | 0.001 | 0.010 | 0.002 |
| *mut2* | -0.023 | 0.005 | -0.024 | 0.003 | 0.002 | 0.001 | 0.025 | 0.001 |
| *mut3* | 0.001 | 0.001 | -0.013 | 0.002 | -0.010 | 0.002 | -0.006 | 0.002 |
| *mut4* | 0.003 | 0.001 | 0.005 | 0.001 | 0.006 | 0.003 | 0.013 | 0.002 |
| *mut5* | 0.003 | 0.002 | 0.009 | 0.002 | 0.011 | 0.004 | 0.011 | 0.003 |
| *mut6* | 0.003 | 0.001 | 0.004 | 0.004 | 0.003 | 0.002 | 0.007 | 0.001 |
| *mut7* | 0.001 | 0.001 | -0.002 | 0.002 | 0.001 | 0.002 | 0.001 | 0.002 |
| *mut8* | 0.016 | 0.002 | 0.031 | 0.006 | 0.035 | 0.006 | 0.041 | 0.002 |
| *mut9* | N/A |  | N/A |  | N/A |  | N/A |  |
| *mCherry* ^c^ |  |  |  |  |  |  |  |  |
| *mut1* | 0.011 | 0.001 | 0.010 | 0.002 | 0.015 | 0.003 | 0.015 | 0.000 |
| *mut2* | -0.012 | 0.002 | -0.017 | 0.001 | 0.014 | 0.001 | 0.039 | 0.005 |
| *mut3* | 0.011 | 0.003 | -0.012 | 0.000 | -0.010 | 0.001 | -0.002 | 0.002 |
| *mut4* | 0.011 | 0.002 | 0.006 | 0.004 | 0.012 | 0.002 | 0.016 | 0.004 |
| *mut5* | 0.017 | 0.002 | 0.014 | 0.002 | 0.015 | 0.002 | 0.019 | 0.003 |
| *mut6* | 0.012 | 0.003 | 0.007 | 0.003 | 0.010 | 0.001 | 0.014 | 0.002 |
| *mut7* | 0.008 | 0.002 | 0.001 | 0.003 | 0.002 | 0.002 | 0.002 | 0.001 |
| *mut8* | 0.023 | 0.003 | 0.026 | 0.001 | 0.032 | 0.001 | 0.039 | 0.003 |
| *mut9* | -0.008 | 0.002 | 0.005 | 0.003 | 0.006 | 0.002 | 0.008 | 0.001 |

a. Fitness is the selection coefficient s with its SEM, obtained from the estimated change in relative abundance compared to the parental reference strain as a function of generations.

b. YECitrine-tagged mutant strains were competed with the mCherry-tagged parental reference strain.

c. mCherry-tagged mutant strains were competed with the YECitrine-tagged parental reference strain.
