# Supplementary material for: Adaptation to High Ethanol Reveals Complex Evolutionary Pathways
Source: PLoS Genet. 2015 Nov 6;11(11):e1005635. doi: 10.1371/journal.pgen.1005635 (PMC4636377; doi:10.1371/journal.pgen.1005635)
Supplement: S7 Table — (DOC) [file pgen.1005635.s031.doc]

**Table S7. Statistical analysis of fitness values of site-directed** mutant strains

| Ethanol (v/v) | 4% | 6% | 8% |
| --- | --- | --- | --- |
|  | *p-val* ^a^ | *p-val* ^a^ | *p-val* ^a^ |
| *mut1* | 0.848 | 0.669 | 0.427 |
| *mut2* | 0.615 | 3.00 e^-4^ | <1.00 e^-4^ |
| *mut3* | 1.47 e^-2^ | 3.29 e^-2^ | 0.064 |
| *mut4* | 0.798 | 0.718 | 0.199 |
| *mut5* | 0.855 | 0.646 | 0.243 |
| *mut6* | 0.726 | 0.870 | 0.486 |
| *mut7* | 0.423 | 0.644 | 0.607 |
| *mut8* | 0.285 | 0.091 | 7.3 e^-3^ |
| *mut9* | 3.30 e^-2^ | 1.58 e^-2^ | 1.50 e^-3^ |

a. P-values are from the one-way ANOVA tests between differences in fitness of 4-8% ethanol treatments compared to no ethanol treatment. Tests were performed taking into account all repetitions from both dye-swaps.
